# A model reduction method for biochemical reaction networks

**DOI:** 10.1186/1752-0509-8-52

**Published:** 2014-05-03

**Authors:** Shodhan Rao, Arjan van der Schaft, Karen van Eunen, Barbara M Bakker, Bayu Jayawardhana

**Affiliations:** 1 Systems Biology Center for Energy Metabolism and Ageing, University of Groningen, ERIBA, Antonius Deusinglaan 1 9713 AV Groningen, Netherlands; 2 Johann Bernoulli Institute for Mathematics and Computer Science, University of Groningen, P.O. Box 407, 9700 AK Groningen, Netherlands; 3 Department of Pediatrics, Center for Liver, Digestive and Metabolic Diseases, University Medical Center Groningen, University of Groningen, Hanzeplein 1, 9713 GZ, Groningen, Netherlands; 4 Institute of Technology and Management, Nijenborgh 4, University of Groningen, 9747 AG Groningen, Netherlands

**Keywords:** Kinetic models, Enzyme kinetics, Complex graph, Weighted Laplacian, Yeast glycolysis, Rat liver beta oxidation

## Abstract

**Background:**

In this paper we propose a model reduction method for biochemical reaction networks governed by a variety of reversible and irreversible enzyme kinetic rate laws, including reversible Michaelis-Menten and Hill kinetics. The method proceeds by a stepwise reduction in the number of complexes, defined as the left and right-hand sides of the reactions in the network. It is based on the Kron reduction of the weighted Laplacian matrix, which describes the graph structure of the complexes and reactions in the network. It does not rely on prior knowledge of the dynamic behaviour of the network and hence can be automated, as we demonstrate. The reduced network has fewer complexes, reactions, variables and parameters as compared to the original network, and yet the behaviour of a preselected set of significant metabolites in the reduced network resembles that of the original network. Moreover the reduced network largely retains the structure and kinetics of the original model.

**Results:**

We apply our method to a yeast glycolysis model and a rat liver fatty acid beta-oxidation model. When the number of state variables in the yeast model is reduced from 12 to 7, the difference between metabolite concentrations in the reduced and the full model, averaged over time and species, is only 8%. Likewise, when the number of state variables in the rat-liver beta-oxidation model is reduced from 42 to 29, the difference between the reduced model and the full model is 7.5%.

**Conclusions:**

The method has improved our understanding of the dynamics of the two networks. We found that, contrary to the general disposition, the first few metabolites which were deleted from the network during our stepwise reduction approach, are not those with the shortest convergence times. It shows that our reduction approach performs differently from other approaches that are based on time-scale separation. The method can be used to facilitate fitting of the parameters or to embed a detailed model of interest in a more coarse-grained yet realistic environment.

## Background

A kinetic model of a biochemical reaction network consists of a set of ordinary differential equations describing the dynamics of the concentrations of all metabolites in the reaction network. Most biochemical reaction networks are complex and involve many enzyme-catalyzed processes with non-linear kinetics and intricate stoichiometric and regulatory interactions between the enzymes. Consequently, the mathematical models of such networks contain high-dimensional sets of coupled rational differential equations, which sometimes require huge computational effort to analyze. The current state-of-the-art numerical tools for stability analysis, for bifurcation study and for other types of dynamical analysis are known to suffer from a so-called *curse-of-dimensionality*. For example, the largest biological model that has numerically been analyzed for bifurcation in [1] consists of 25 metabolites and 37 parameters and the one in [2] has 22 metabolites. Moreover since complex models of biochemical reaction networks involve a huge number of parameters, the task of identifying these parameters (in addition to those parameters that have been identified in the literature) is enormous and requires large datasets. The complexity of this task is further compounded by the fact that often not all the metabolite concentrations can be measured. Thus, there is a need for techniques that can reduce a given kinetic model of a biochemical reaction network to a simplified version that mimics the behaviour of the original model satisfactorily, but contains less differential equations and parameters.

For biochemical reaction networks, a number of model reduction techniques are known. See [3] for a detailed review of some of the well known methods of model reduction. Here we list only a few of the known methods in the literature. The singular perturbation method, the time-scale separation technique [4-8], the rapid-equilibrium approximation, also known as the quasi-equilibrium approximation (see [9]) and the quasi steady-state approximation (QSSA) (see for e.g., [10]) are the most commonly used techniques. The reduced state vector obtained by any of these techniques contains a subset of the metabolite concentrations (state vector components) of the full model. Härdin [11] extends the QSSA approach by considering the higher-order approximation in the computation of the quasi steady-state. In [12], some approaches for reduction of complexity in biochemical reaction networks are considered for use in SYCAMORE, which is a computational research environment in systems biology. One of the approaches considered in [12] is the intrinsic low-dimensional manifold (ILDM) approach, which is an improved time-scale separation technique. More recently [13], a computational singular perturbation (CSP) algorithm was developed to analyze the time-scales of the NF- *κ*B signaling network which could be used in order to reduce its model. The application of singular perturbation, time-scale separation, quasi equilibrium and quasi steady state approaches to general enzyme-kinetic rate laws, such as Michaelis-Menten and ping-pong bi-bi is difficult and leads to complicated rate law expressions in the reduced models. Some of the time-scale separation techniques are either based on a priori experimental information or eigenvalues of the Jacobian corresponding to the model and hence are only locally effective. Another recent approach for model reduction uses tropical geometry (see e.g., [14]), wherein the polynomial occuring in every rate equation is replaced by a monomial which is equal to the largest, in absolute value among the monomials that constitute the polynomial.

One of the ways of reducing the complexity of a biochemical model is to reduce the number of parameters in it. This can be done by carrying out a parameter sensitivity analysis (see for e.g., [15]) and eliminating those parameters whose variations have least effect on the dynamics of a given network. In [8], a time-scale analysis is first done using experimental data to identify the fast and the slow metabolites and this information is then used to carry out an *a priori* parameter sensitivity analysis to obtain a reduced kinetic model of a biochemical reaction network. In [16], an implicit multiparametric variability analysis (MPVA) method is used to search the entire parameter space in order to determine the set of parameters that can be eliminated. In [17], the authors go one step further by identifying a region in the parameter space where some of the parameters are zero-valued, others have readjusted values and where nevertheless the outputs of the original model match those of the reduced model within a certain tolerance. They further show that parameter sensitivity analysis approach for model reduction may not be always successful. Another way of reducing the complexity of a biochemical reaction network model is to reduce the number of reactions. In [18-20], optimization techniques are used to determine which reactions can be eliminated from the original model without a substantial alteration of the model behaviour.

The method proposed in [21] simplifies a given chemical reaction network by linearly combining reactions in such a way that the resulting network has lesser number of species. However, the kinetics for the reduced set of reactions are determined by the rate limiting steps in the original network and this requires prior biological knowledge of the network. Danø *et al.*[22] propose reduction by identification and elimination of variables in such a way that the basic dynamic properties of the original model are preserved. In [23], model reduction is achieved by simplifying the rate equations of individual enzymes in the network. A limitation of this method is that it yields a reduced model that predicts measurement data only under specific experimental conditions.

In this paper, we describe a new model reduction method that reduces the number of reactions, metabolites and parameters, such that the dynamics of the metabolite concentrations of the reduced model are close to those of the original model. This method proceeds by a simple stepwise reduction in the number of ‘complexes’, which are defined as the left and right-hand sides of the reactions in the network. The effect of this stepwise reduction is monitored by an error integral, which quantifies how much the behaviour of the reduced model deviates from the original. The method is based on the reduction of the underlying weighted Laplacian (see [24] for a definition) describing the graph structure of the complexes and reactions in the chemical reaction network. A similar technique is also employed in the Kron reduction method for reduction of resistive electrical network models [25].

Our model reduction technique is easy to implement and can be used to reduce reaction networks governed by a vast majority of enzyme kinetic rate laws including Hill kinetics and reversible Michaelis-Menten kinetics. It does not rely on prior knowledge about the dynamic behaviour or biological function of the network. Consequently, it can be automated. Furthermore, the reduced model largely retains the kinetics and structure of the original model. This enables a direct biochemical interpretation and yields insight into which parts of the network have the highest influence on its behaviour. It also accelerates computations and facilitates parameter fitting, especially when we deal with models of huge biochemical reaction networks.

We show the application of our model reduction technique to a yeast glycolysis model [26] and a model of beta-oxidation in rat liver [27]. We have simulated the transient behaviour of the metabolites that are not eliminated during the model reduction procedure. In both the cases, a 30% reduction of the number of variables still retained about 92.5–96.5% agreement between the outputs of the full and the reduced networks.

## Methods

### Preliminaries

Notation: The space of *n* dimensional real vectors is denoted by $R^{n}$, and the space of *m*×*n* real matrices by $R^{m\times n}$. The space of *n* dimensional real vectors consisting of all strictly positive entries is denoted by $R_{+}^{n}$. *I*_*n*_ denotes an identity matrix of dimension *n*. The transpose of a matrix *A* is denoted by *A*^*T*^. Define the mapping $\text{Ln}:R_{+}^{m}\to R^{m},\;x\mapsto \text{Ln}(x),$ as the mapping for which the *i*-th component is given by (Ln(*x*))_*i*_:=ln(*x*_*i*_). Similarly, define the mapping $\text{Exp}:R^{m}\to R_{+}^{m},\;x\mapsto \text{Exp}(x)$, as the mapping for which the *i*-th component is given by (Exp(*x*))_*i*_:=exp(*x*_*i*_). $1_{m}$ denotes a vector of dimension *m* with all entries equal to 1 and dim(*V*) denotes the dimension of a set *V*.

#### Chemical reaction network structure

In this section, we introduce the concept of a complex graph which was first introduced in the work of Horn & Jackson and Feinberg [28-30]. This concept will be used first in deriving our general mathematical formulation of the dynamics of chemical reaction networks, and subsequently to explain our model reduction approach.

**Figure 1:**
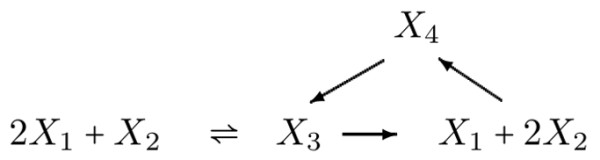
Example of a reaction network.

Let *m*, *c* and *r* denote the number of species (metabolites), complexes and reactions respectively of a given chemical reaction network. The set of complexes of a network is simply defined as the union of all the different left- and righthand sides (substrates and products) of the reactions in the network. Thus, the complexes corresponding to the network in Figure 1 are 2*X*_1_+*X*_2_, *X*_3_, *X*_1_+2*X*_2_ and *X*_4_.

The expression of the complexes in terms of the chemical species is formalized by an *m*×*c* matrix *Z*, whose *α*-th column captures the expression of the *α*-th complex in the *m* chemical species. For example, for the network depicted in Figure 1,

$$Z=\left[\begin{array}{cccc} 2 & 0 & 1 & 0\\ 1 & 0 & 2 & 0\\ 0 & 1 & 0 & 0\\ 0 & 0 & 0 & 1 \end{array}\right].$$

The matrix *Z* is called the *complex stoichiometric matrix* of the network. Note that by definition all elements of the matrix *Z* are non-negative integers.

Since complexes are the left- and righthand sides of reactions in the network, they can be naturally associated with the vertices of a *directed graph* with edges corresponding to the reactions. Formally, the reaction *α*→*β* between the *α*-th (reactant) and the *β*-th (product) complexes defines a directed edge with tail vertex being the *α*-th complex and head vertex being the *β*-th complex. The resulting graph will be called the *complex graph*.

Any graph is defined by its *incidence matrixB*[24]. This is a *c*×*r* matrix, where *c* denotes the number of vertices, *r* denotes the number of edges and the (*α*,*j*)-th element is equal to -1 if vertex *α* is the tail vertex of edge *j* and 1 if vertex *α* is the head vertex of edge *j*, while 0 otherwise.

The basic structure underlying the dynamics of the vector $x\in R_{+}^{m}$ of concentrations *x*_*i*_,*i*=1,⋯,*m*, of the species of a chemical reaction network is given by the *balance laws*$\dot{x}=\text{ZBv}(x)$; the elements of the vector $v\in R^{r}$ are commonly called the (reaction) *rates* or *fluxes*.

In many cases of interest, especially in biochemical reaction networks, chemical reaction networks are intrinsically *open*, in the sense that there is a continuous exchange with the environment (in particular, inflow and outflow of chemical species and connection to other reaction networks). In this paper, we are particularly interested in inflows to and outflows from the complexes of the network. This will be modeled by a vector $v_{b}(x)\in R^{c}$ consisting of *cboundary* (or, exchange) *fluxes*, leading to an extended model

(1) $$\dot{x}=\text{ZBv}(x)+{\text{Zv}}_{b}(x)$$

A *linkage class* of a chemical reaction network is a maximal set of complexes $\left\{C_{1},\ldots ,C_{k}\right\}$ such that $C_{i}$ is connected by reactions to $C_{j}$ for every *i*,*j*∈{1,…,*k*}, *i*≠*j*.

#### General kinetics

For a biochemical reaction network, the relation between the reaction rates and species concentrations depends on the mechanisms of the reactions involved in the network. In this section, we derive a framework for describing the dynamics of enzymatic reaction networks using the aforementioned notion of complex graphs in a reaction network. This framework will be useful in describing our model reduction method. We describe the forward and reverse rates of an enzyme with separate equations.

Let $Z_{S_{j}}$ denote the column of the complex stoichiometric matrix *Z* corresponding to the substrate complex $S_{j}$ of the *j*-th reaction of a chemical reaction network. Then the unidirectional, forward reaction rate of the *j*^th^ reaction of the chemical reaction network between the substrate complex $S_{j}$ and the product complex $P_{j}$ is given by

(2) $$v_{j}(x)=d_{j}(x)k_{j}\exp\left(\right)Z_{S_{j}}^{T}\text{Ln}(x))),$$

where for *j*=1,…,*r*, $d_{j}:R_{+}^{m}\to R_{+}$ is a rational function of its argument and *k*_*j*_ denotes a proportionality constant of the *j*^th^ reaction of the network. Note that if the governing law of the *j*^th^ reaction is *mass action kinetics*, then *d*_*j*_(*x*)=1.

As an example, consider the reaction

(3) $$X_{1}+X_{2}\to X_{3}+X_{4}$$

For *i*=1,…,4, let *x*_*i*_ denote the concentration of the species *X*_*i*_ and define *x*:=[*x*_1_*x*_2_*x*_3_*x*_4_]^*T*^. In this example, the substrate complex *S* is *X*_1_+*X*_2_ and the product complex *P* is *X*_3_+*X*_4_. The matrices *B* and *Z* for the reaction (3) are given by

$$\begin{array}{c} B=\left[\begin{array}{c} -1\\ 1 \end{array}\right]\;\;Z=\left[\begin{array}{cc} 1 & 0\\ 1 & 0\\ 0 & 1\\ 0 & 1 \end{array}\right]. \end{array}$$

Hence $Z_{S}$ is given by $Z_{S}={\left[1\;1\;0\;0\right]}^{T}$. Let *K*_1_, *K*_2_, *K*_3_ and *K*_4_ denote the “Michaelis” constants of the species *X*_1_, *X*_2_, *X*_3_ and *X*_4_ respectively. Let *V*_*f*_ denote the maximum rate of the forward reaction (3). The expression for the rate of the reaction (3) depends on the type and mechanism of the reaction, for instance the type of inhibition and other regulatory mechanisms involved in the reaction. One possibility is

(4) $$v(x)=\frac{\left(\frac{V_{f}}{K_{1}K_{2}}\right)x_{1}x_{2}}{\left(1+\frac{x_{1}}{K_{1}}+\frac{x_{3}}{K_{3}}\right)\left(1+\frac{x_{2}}{K_{2}}+\frac{x_{4}}{K_{4}}\right)}.$$

By defining $k:=\frac{V_{f}}{K_{1}K_{2}}$,

$$d(x):=\frac{1}{\left(1+\frac{x_{1}}{K_{1}}+\frac{x_{3}}{K_{3}}\right)\left(1+\frac{x_{2}}{K_{2}}+\frac{x_{4}}{K_{4}}\right)},$$

observe that $d:R_{+}^{4}\to R_{+}$ and equation (4) can be written as

$$v(x)=d(x){\text{kx}}_{1}x_{2}=d(x)k\exp\left(\right)Z_{S}^{T}\text{Ln}(x))).$$

In Additional file 1, we have provided a list of enzyme-kinetic rate laws that can be written in the form (2) with *d* satisfying $d:R_{+}^{m}\to R_{+}$. Note that the form (2) is retained with a *d* satisfying $d:R_{+}^{m}\to R_{+}$ even if there are competitive, non-competitive or uncompetitive modifiers in the reaction network. This is because the terms containing the concentration of the modifiers appear in the denominator of the rate expression in such a way that the denominator assumes positive values for positive values of concentrations of the substrates. For example, if we consider the reaction

(5) $$X_{1}\to X_{2}$$

governed by Michaelis-Menten kinetics, with Michaelis constant of *X*_1_ denoted by *K*_1_ and the maximum reaction rate denoted by *V*, then

(6) $$v(x)=\frac{\frac{{\text{Vx}}_{1}}{K_{1}}}{1+\frac{x_{1}}{K_{1}}}$$

is the expression for the rate of the reaction (5). It is easy to see that with $k:=\frac{V}{K_{1}}$ and $d(x):={\left(1+\frac{x_{1}}{K_{1}}\right)}^{-1}$, (6) has the same form as (2) and $d:R_{+}^{2}\to R_{+}$. Now assume that the reaction involves a competitive modifier *I* whose concentration is denoted by *i* and whose inhibition coefficient is denoted by *K*_*i*_. Then the rate of the reaction is given by

(7) $$v_{c}(x)=\frac{\frac{{\text{Vx}}_{1}}{K_{1}}}{1+\frac{x_{1}}{K_{1}}+\frac{i}{K_{i}}}$$

With *k* defined as earlier and $d(x):={\left(1+\frac{x_{1}}{K_{1}}+\frac{i}{K_{i}}\right)}^{-1}$ observe that (7) has the same form as (2) and $d:R_{+}^{2}\to R_{+}$. Similarly if *I* is a non-competitive modifier, then

$$v_{\text{nc}}(x)=\frac{\frac{{\text{Vx}}_{1}}{K_{1}}}{\left(1+\frac{x_{1}}{K_{1}}\right)\left(1+\frac{i}{K_{i}}\right)}$$

denotes the rate of the reaction which can again be written in the form (2) with a *d* satisfying $d:R_{+}^{2}\to R_{+}$. If *I* denotes an uncompetitive modifier, then

$$v_{\text{uc}}(x)=\frac{\frac{{\text{Vx}}_{1}}{K_{1}}}{1+\frac{x_{1}}{K_{1}}\left(1+\frac{i}{K_{i}}\right)}$$

denotes the rate of the reaction which can be written in the form (2) with a *d* satisfying $d:R_{+}^{2}\to R_{+}$.

Based on the formulation in (1) and (2), we can describe the complete reaction network dynamics as follows. For every *σ*,*π*∈{1,…,*c*}, define

$$C_{\pi \sigma }:=\left\{j\in \{1,\ldots ,r\}|(\sigma ,\pi )=(S_{j},P_{j})\right\}$$

and $a_{\pi \sigma }:=\underset{j\in C_{\pi \sigma }}{\sum }k_{j}d_{j}(x)$. Thus if there is no reaction *σ*→*π*, then *a*_*π* *σ*_=0. Define the *weighted adjacency matrixA* of the complex graph as the matrix with (*σ*,*π*)-th element *a*_*σ* *π*_, where *σ*,*π*∈{1,⋯,*c*}. Furthermore, define *L*(*x*):=*Δ*(*x*)-*A*(*x*), where *Δ* is the diagonal matrix whose (*ρ*,*ρ*)-th element is equal to the sum of the elements of the *ρ*-th column of *A*. Hereafter we refer to *L*(*x*) as the *weighted Laplacian* of the complex graph associated with the given network with species concentration vector *x*. It can be verified that the sum of the elements of each column of *L* is equal to zero and that the vector *Bv*(*x*) is equal to -*L*(*x*)Exp(*Z*^*T*^Ln(*x*)). From equation (1), it follows that the dynamics of enzymatic chemical reaction networks can be compactly written as

(8) $$\dot{x}=-\text{ZL}(x)\text{Exp}\left(Z^{T}\text{Ln}(x)\right)+{\text{Zv}}_{b}(x)$$

A similar expression of the dynamics corresponding to mass action kinetics, in less explicit form, was obtained in [31]. Note that although in equation (8), Exp(*Z*^*T*^Ln(*x*)) is not defined if some of the components of *x* are equal to zeros, the limit at such an *x* exists and is used in equation (8) whenever some of the components of *x* are equal to zero.

### Model reduction

For many purposes one may wish to reduce the number of dynamical equations of a chemical reaction network in such a way that the behaviour of a number of key metabolites is approximated in a satisfactory way. We propose a novel method for model reduction of chemical reaction networks governed by enzyme kinetics. Our method is inspired by the Kron reduction method for model reduction of resistive electrical networks described in [25]; see also [32].

#### Description of the method

Consider again a reaction network with boundary fluxes and with dynamics modelled by equation (8). Our model reduction method is based on *reduction of the complex graph* associated with the network. Let  denote the set of vertices of the complex graph. Reduction of the model is carried out by *deleting certain complexes in the complex graph*, resulting in a reduced complex graph. Deletion of a complex is equivalent to imposing the complex balancing condition on it, i.e., the condition that the net inflow into the complex is equal to the net outflow from it. Consider a subset $V_{o}\subset V$ of dimension c-ĉ that we wish to delete in order to reduce the model. Consider a partition of *L*(*x*) given by

$$L(x)=\left[\begin{array}{cc} L_{11}(x) & L_{12}(x)\\ L_{21}(x) & L_{22}(x) \end{array}\right]$$

where $L_{11}(x)\in R^{ĉ\times ĉ}$, $L_{12}(x)\in R^{ĉ\times (c-ĉ)}$, $L_{21}(x)\in R^{(c-ĉ)\times ĉ}$, $L_{22}(x)\in R^{\left(c-ĉ)\times (c-ĉ\right)}$ and $V_{o}$ corresponds to the last part of the indices (denoted by 2). Consider corresponding partitions of *Z* and *v*_*b*_ given by

$$Z=\left[\begin{array}{cc} Z_{1} & Z_{2} \end{array}\right];\;v_{b}(x)={\left[\begin{array}{cc} v_{b1}(x) & v_{b2}(x) \end{array}\right]}^{T},$$

in order to rewrite (8) as

$$\begin{array}{c} \dot{x}=Z\left[\begin{array}{c} v_{b1}(x)\\ v_{b2}(x) \end{array}-Z\right]\left[\begin{array}{cc} L_{11}(x) & L_{12}(x)\\ L_{21}(x) & L_{22}(x) \end{array}\right]\left[\begin{array}{c} \text{Exp}\left(\right)Z_{1}^{T}\text{Ln}(x)))\\ \text{Exp}\left(\right)Z_{2}^{T}\text{Ln}(x))) \end{array}\right] \end{array}$$

Define $P:=[I_{ĉ}\;-L_{12}L_{22}^{-1}]$ and let $\hat{L}(x)$ denote the Schur complement of *L*(*x*) with respect to the indices corresponding to $V_{o}$. Consider now the auxiliary dynamical system

$$\left[\begin{array}{c} {\dot{y}}_{1}\\ {\dot{y}}_{2} \end{array}\right]=\left[\begin{array}{c} v_{b1}(x)\\ v_{b2}(x) \end{array}\right]-\left[\begin{array}{cc} L_{11}(x) & L_{12}(x)\\ L_{21}(x) & L_{22}(x) \end{array}\right]\left[\begin{array}{c} w_{1}\\ w_{2} \end{array}\right].$$

Note that complex balancing condition on the complexes in $V_{o}$ can be imposed by setting the constraint ${\dot{y}}_{2}=0$. This results in the equation

$$w_{2}=-L_{22}{\left(x\right)}^{-1}(v_{b2}(x)-L_{21}(x)w_{1}),$$

leading to the reduced auxiliary dynamics

$${\dot{y}}_{1}={\text{Pv}}_{b}(x)-\hat{L}(x)w_{1}.$$

Substituting $w_{1}=\text{Exp}\left(\right)Z_{1}^{T}\text{Ln}(x)))$ in the above equation and making use of $\dot{x}=Z_{1}{\dot{y}}_{1}+Z_{2}{\dot{y}}_{2}=Z_{1}{\dot{y}}_{1}$, we then obtain the reduced model given by

(9) $$\dot{x}=Z_{1}\left({\text{Pv}}_{b}(x)-\hat{L}(x)\text{Exp}\left(\right)Z_{1}^{T}\text{Ln}(x)))\right).$$

Note that the reduced model is independent of the order of deletion of complexes. From the following Proposition, it follows that $\hat{L}(x)$ satisfies all the properties of a weighted Laplacian matrix of a reaction network corresponding to a complex graph with vertex set $V-V_{o}$

##### **Proposition1**.

Consider a chemical reaction network with weighted Laplacian matrix $L(x)\in R^{c\times c}$ corresponding to the concentration vector *x*. Let  denote the set of vertices of the complex graph associated with the network. Then for any subset of vertices $V_{r}\subset V$, the Schur complement $\hat{L}(x)$ of *L*(*x*) with respect to the indices corresponding to $V_{r}$ satisfies the following properties: 

1. All diagonal elements of $\hat{L}(x)$ are positive and off-diagonal elements are nonnegative for $x\in R_{+}^{m}$.

2. $1_{ĉ}^{T}\hat{L}(x)=0$, where $ĉ:=c-\text{dim}(V_{r})$.

##### *Proof*

(*1*.) Follows from the proof of ( [33], Theorem 3.11); see also [32] for the case of symmetric *L*. (*2*.) Without loss of generality, assume that the last c-ĉ rows and columns of *L*(*x*) correspond to the vertex set $V_{r}$. Consider a partition of *L*(*x*) given by

(10) $$L(x)=\left[\begin{array}{cc} L_{11}(x) & L_{12}(x)\\ L_{21}(x) & L_{22}(x) \end{array}\right]$$

where $L_{11}(x)\in R^{ĉ\times ĉ}$, $L_{12}(x)\in R^{ĉ\times (c-ĉ)}$, $L_{21}(x)\in R^{(c-ĉ)\times ĉ}$ and $L_{22}(x)\in R^{\left(c-ĉ)\times (c-ĉ\right)}$. It is easy to see that

$$\hat{L}(x)=L_{11}(x)-L_{12}(x)L_{22}{\left(x\right)}^{-1}L_{21}(x)$$

Since $1_{c}^{T}L(x)=0$, we obtain

$$1_{ĉ}^{T}L_{11}(x)+1_{c-ĉ}^{T}L_{21}(x)=1_{ĉ}^{T}L_{12}(x)+1_{c-ĉ}^{T}L_{22}(x)=0$$

Using the above equations, we get

$$\begin{array}{lll} 1_{ĉ}^{T}\hat{L}(x) & =1_{ĉ}^{T}\left(\right)L_{11}(x)-L_{12}(x)L_{22}{\left(x\right)}^{-1}L_{21}(x)))\; & \;\\ & =-1_{c-ĉ}^{T}L_{21}(x)+1_{c-ĉ}^{T}L_{22}(x)L_{22}{\left(x\right)}^{-1}L_{21}(x)=0\; & \; \end{array}$$

□

This proves that (9) describes the dynamics of a *chemical reaction network* governed by enzyme kinetics, with a reduced number of complexes and with, in general, a different set of boundary fluxes and reactions (edges of the complex graph). An appropriate choice of $V_{o}$ will ensure that some of the elements of *x* have derivative zero in (9) leading to a reduced number of state variables.

The principle behind our model reduction method is to couple the dynamics of certain complexes to the dynamics of the neighbouring complexes or the complexes with which they are connected by reactions. This is done by complex balancing or by equating the net rate of inflow into the complex to the net rate of outflow from it. Note that for a reasonably good model reduction, it is important to make the right choice of complexes to be deleted. In the next section, we describe a procedure for making the choice of complexes to be deleted. Below, we illustrate our complex balancing procedure for model reduction with the help of an example.

##### Example 1

We consider an example of a simple reversible reaction network and apply the model reduction procedure described in the paper. This reaction network is shown below:

(11) $$X_{1}+X_{2}⇌X_{3}+X_{4}⇌X_{5}+X_{6}$$

For *i*=1,…,6, let *x*_*i*_ denote the concentration of *X*_*i*_. For *i*=1,…,4, let $K_{m}^{I,i}$ denote the Michaelis constant of *X*_*i*_ for the first reversible reaction. For *i*=3,…,6, let $K_{m}^{\text{II},i}$ denote the Michaelis constant of *X*_*i*_ for the second reversible reaction. Let $k_{f}^{I}$ and $k_{r}^{I}$ denote the forward and reverse rate constants of the first reaction and let $k_{f}^{\text{II}}$ and $k_{r}^{\text{II}}$ denote the respective rate constants of the second reaction. Note that $k_{f}^{I}:=\frac{V_{f}^{I}}{K_{m}^{I,1}K_{m}^{I,2}}$ where $V_{f}^{I}$ denotes the maximum rate of the first reversible reaction in the forward direction and the other rate constants are similarly defined. Define *x*:=[*x*_1_*x*_2_ … *x*_6_]^*T*^,

$$\begin{array}{c} p_{1}(x):=\left(1+\frac{x_{1}}{K_{m}^{I,1}}+\frac{x_{3}}{K_{m}^{I,3}}\right)\left(1+\frac{x_{2}}{K_{m}^{I,2}}+\frac{x_{4}}{K_{m}^{I,4}}\right),\\ p_{2}(x):=\left(1+\frac{x_{3}}{K_{m}^{\text{II},3}}+\frac{x_{5}}{K_{m}^{\text{II},5}}\right)\left(1+\frac{x_{4}}{K_{m}^{\text{II},4}}+\frac{x_{6}}{K_{m}^{\text{II},6}}\right). \end{array}$$

As described for the example in the section “General kinetics”, possible rate equations for the network are given by $v_{1f}(x)=\frac{k_{f}^{I}x_{1}x_{2}}{p_{1}(x)}$, $v_{1r}(x)=\frac{k_{r}^{I}x_{3}x_{4}}{p_{1}(x)}$, $v_{2f}(x)=\frac{k_{f}^{\text{II}}x_{3}x_{4}}{p_{2}(x)}$ and $v_{2r}(x)=\frac{k_{r}^{\text{II}}x_{5}x_{6}}{p_{2}(x)}$, where *v*_1*f*_,*v*_1*r*_ denote the reaction rates in the forward and the reverse directions respectively of the first reversible reaction and *v*_2*f*_,*v*_2*r*_ similarly denote those of the second reversible reaction. If $K_{\text{eq}}^{I}$ and $K_{\text{eq}}^{\text{II}}$ denote the equilibrium constants of the first and the second reversible reactions respectively, then

$$K_{\text{eq}}^{I}=\frac{k_{f}^{I}}{k_{r}^{I}}\;K_{\text{eq}}^{\text{II}}=\frac{k_{f}^{\text{II}}}{k_{r}^{\text{II}}}$$

Now consider the reduced network

$$X_{1}+X_{2}⇌X_{5}+X_{6}$$

that is obtained by deleting the complex *X*_3_+*X*_4_ from the network (11). Applying the procedure described in this section, we first assume that the rate of inflow to the complex *X*_3_+*X*_4_ is equal to the rate of outflow from it, i.e. *v*_1*f*_-*v*_1*r*_=*v*_2*f*_-*v*_2*r*_. This yields

(12) $$x_{3}x_{4}=\frac{k_{f}^{I}p_{2}(x)x_{1}x_{2}+k_{r}^{\text{II}}p_{1}(x)x_{5}x_{6}}{k_{f}^{\text{II}}p_{1}(x)+k_{r}^{I}p_{2}(x)}$$

By substitution, we obtain the following expression for the overall rate *v* in the forward direction of the reduced network:

$$\begin{array}{c} v(x)\;=\;v_{1f}(x)-v_{1r}(x)=v_{2f}(x)-v_{2r}(x)=\frac{k_{f}^{I}k_{f}^{\text{II}}x_{1}x_{2}-k_{r}^{I}k_{r}^{\text{II}}x_{5}x_{6}}{k_{f}^{\text{II}}p_{1}(x)+k_{r}^{I}p_{2}(x)}. \end{array}$$

In the expression for *p*_1_ and *p*_2_ in the right hand side of the above equation, we fix *x*_3_ and *x*_4_ at their initial values to obtain the following expression for *v*.

(13) $$v(x)\;=\;\frac{k_{f}^{\text{red}}x_{1}x_{2}-k_{r}^{\text{red}}x_{5}x_{6}}{1\;+\;\frac{x_{1}}{K_{m}^{\text{red},1}}\;+\;\frac{x_{2}}{K_{m}^{\text{red},2}}\;+\;\frac{x_{5}}{K_{m}^{\text{red},5}}\;+\;\frac{x_{6}}{K_{m}^{\text{red},6}}\;+\;\frac{x_{1}x_{2}}{K_{m}^{\text{red},12}}\;+\;\frac{x_{5}x_{6}}{K_{m}^{\text{red},56}}}.$$

where $k_{f}^{\text{red}},k_{r}^{\text{red}},K_{m}^{\text{red},1},K_{m}^{\text{red},2},K_{m}^{\text{red},5},K_{m}^{\text{red},6},K_{m}^{\text{red},12},K_{m}^{\text{red},56}$ are positive constants that depend on the rate constants and Michaelis constants of the original network and the initial values of *x*_3_ and *x*_4_. We remark here that the resulting equation (13) has again the form of an enzyme kinetic rate equation and the rates in the forward and reverse directions can be written in the form of equation (2) with a *d* satisfying $d:R_{+}^{4}\to R_{+}$. If $K_{\text{eq}}^{\text{red}}$ denotes the equilibrium constant for the reduced network, then observe that

$$K_{\text{eq}}^{\text{red}}=\frac{k_{f}^{I}k_{f}^{\text{II}}}{k_{r}^{I}k_{r}^{\text{II}}}=K_{\text{eq}}^{I}K_{\text{eq}}^{\text{II}}$$

For this example, our procedure yields a reduced model with 8 parameters while the original model has 12 parameters. Moreover while the original model has 2 reactions and 6 state variables, the reduced model has 1 reaction and 4 state variables.

#### Error integral

We now describe an automated procedure for making the choice of complexes to be deleted in such a way that the dynamic behaviour of the reduced model is close to that of the original model. We quantify the difference between the dynamical behaviour of the original and the corresponding reduced model under the conditions of a specific dynamic event by an error integral, as defined in this section.

Assume that the given biochemical network is asymptotically stable around a steady state. Let ${ℳ}_{I}$ denote the set of species which we consider to be significant from the point of view of experimental design. This set is a subjective choice of the scientist and contains for instance species whose concentrations can be experimentally measured. Moreover complexes made of species in ${ℳ}_{I}$ are not considered for deletion in order to reduce the model. Let $n({ℳ}_{I})$ denote the number of elements in the set ${ℳ}_{I}$. Assume that [0,*T*] is the time interval over which we are interested to observe the difference between the behaviours of the full and the reduced model. We define the error integral (*I*) as

(14) $$I:=\underset{i\in {ℳ}_{I}}{\sum }\frac{1}{\text{Tn}({ℳ}_{I})}\int_{0}^{T}\left|1-\frac{x_{\text{ir}}(t)}{x_{\text{if}}(t)}\right|\text{dt},$$

where *x*_*i* *r*_(*t*) and *x*_*i* *f*_(*t*) denote the concentrations of the *i*^th^ metabolite of the reduced and the full model respectively at time *t*. Observe that *I* is dimensionless. The value of *I* indicates the deviation of the reduced model from the full model averaged over time and species.

In order to reduce a given model of a biochemical reaction network, we first determine the steady state concentration of each of the metabolite of the network. Then we rank the complexes to be deleted according to the error integrals corresponding to the reduced model with the respective complex deleted and with the initial values of concentrations of the species of the deleted complexes set at their steady state values. We then make the deletion that leads to the smallest value of *I*. With the reduced model, we then repeat this procedure. Thus we follow an iterative procedure with each iteration consisting of two steps: ranking of complexes to be deleted and deletion of the complex which leads to the smallest error integral. We stop the iteration when the error integral of the reduced model obtained at the end of an iteration exceeds a certain cut-off value. The reduced model obtained at the end of the previous iteration is then considered as the final reduced model.

For the two biochemical models considered in this paper, namely the yeast glycolysis model and the rat-liver fatty acid beta-oxidation model, the cut-off value of the error integral for stopping the iterative model reduction procedure has been set at 0.1. In general, this cut-off value can be set according to the desired closeness of the reduced model to the original.

#### Difference with QSSA

The basic premise of our model reduction approach is similar to the one of quasi steady state approximation (QSSA). In our method, we assume that some of the complexes attain rapid steady states and hence we equate the rates of inflows to and outflows from such complexes. On the other hand, in the case of QSSA, it is rather some of the individual metabolites that are assumed to attain rapid steady states and hence the rates of inflows to and outflows from such metabolites are equated. There are some other subtle differences between our approach and QSSA even for the case when the complexes are given by the species, i.e., *Z* is an identity matrix, as shown in the following example. Consider the reaction network

$$X_{1}⇌X_{2}⇌X_{3}$$

with reactions governed by reversible Michaelis-Menten kinetics. As earlier, for *i*=1,2,3, let *x*_*i*_ denote the concentration of *X*_*i*_. For *i*=1,2, let $K_{m}^{I,i}$ denote the Michaelis constant of *X*_*i*_ for the first reversible reaction. For *i*=2,3, let $K_{m}^{\text{II},i}$ denote the Michaelis constant of *X*_*i*_ for the second reversible reaction. Let $k_{f}^{I}$ and $k_{r}^{I}$ denote the forward and reverse rate constants of the first reaction and let $k_{f}^{\text{II}}$ and $k_{r}^{\text{II}}$ denote the respective rate constants of the second reaction. Note that $k_{f}^{I}:=\frac{V_{f}^{I}}{K_{m}^{I,1}}$ where $V_{f}^{I}$ denotes the maximum rate of the first reversible reaction in the forward direction and the other rate constants are similarly defined. Define

$$\begin{array}{c} p_{1}(x_{1},x_{2}):=\left(1+\frac{x_{1}}{K_{m}^{I,1}}+\frac{x_{2}}{K_{m}^{I,2}}\right)\\ p_{2}(x_{2},x_{3}):=\left(1+\frac{x_{2}}{K_{m}^{\text{II},2}}+\frac{x_{3}}{K_{m}^{\text{II},3}}\right). \end{array}$$

Now assume that *X*_2_ attains rapid steady state which, as in the previous example, implies that

$$x_{2}=\frac{k_{f}^{I}p_{2}(x_{2},x_{3})x_{1}+k_{r}^{\text{II}}p_{1}(x_{1},x_{2})x_{3}}{k_{f}^{\text{II}}p_{1}(x_{1},x_{2})+k_{r}^{I}p_{2}(x_{2},x_{3})}.$$

If we use QSSA method, we first need to solve for *x*_2_ from the resulting quadratic equation given by

$$\begin{array}{l} \begin{array}{c} x_{2}^{2}\left(\frac{k_{f}^{\text{II}}}{K_{m}^{I,2}}+\frac{k_{r}^{I}}{K_{m}^{\text{II},2}}\right)+x_{2}\left(k_{f}^{\text{II}}+k_{r}^{I}+\frac{k_{f}^{\text{II}}x_{1}}{K_{m}^{I,1}}-\frac{k_{f}^{I}x_{1}}{K_{m}^{\text{II},2}}+\frac{k_{r}^{I}x_{3}}{K_{m}^{\text{II},3}}-\frac{k_{r}^{\text{II}}x_{3}}{K_{m}^{I,2}}\right)\\ \;\;\;\;-k_{f}^{I}x_{1}\left(1+\frac{x_{3}}{K_{m}^{\text{II},3}}\right)-k_{r}^{\text{II}}x_{3}\left(1+\frac{x_{1}}{K_{m}^{I,1}}\right)=0. \end{array} \end{array}$$

Let *f* be a function of two variables such that *x*_2_=*f*(*x*_1_,*x*_3_) defines an admissible (real and positive) solution of the above equation. Then the resulting reaction rate in the forward direction (*v*) of the reduced network

(15) $$X_{1}⇌X_{3}$$

is given by

$$v(x)=\frac{k_{f}^{I}k_{f}^{\text{II}}x_{1}-k_{r}^{I}k_{r}^{\text{II}}x_{3}}{k_{f}^{\text{II}}p_{1}(x_{1},f(x_{1},x_{3}))+k_{r}^{I}p_{2}(f(x_{1},x_{3}),x_{3})}.$$

The kinetics of the reduced network obtained by application of QSSA in this case is no longer enzyme kinetics. In contrast, our model reduction method yields the simple expression

$$v(x)=\frac{k_{f}^{\text{red}}x_{1}-k_{r}^{\text{red}}x_{3}}{1+\frac{x_{1}}{K_{m}^{\text{red},1}}+\frac{x_{3}}{K_{m}^{\text{red},3}}}$$

as the overall reaction rate in the forward direction of the reduced network (15).

If we now consider the following reaction network

$$X_{1}+X_{2}⇌2X_{3}⇌X_{4}+X_{5}$$

governed by the same kind of enzyme-kinetic rate laws as in Example 1 and assume that *X*_3_ attains rapid equilibrium, application of QSSA is more complicated as compared to the previous case as it involves solution of a quartic equation. On the other hand, our method leads to a simple expression for the overall reaction rate in the forward direction (*v*) of the reduced network

$$\begin{array}{c} v(x)=\frac{k_{f}^{\text{red}}x_{1}x_{2}-k_{r}^{\text{red}}x_{4}x_{5}}{1+\frac{x_{1}}{K_{m}^{\text{red},1}}+\frac{x_{2}}{K_{m}^{\text{red},2}}+\frac{x_{4}}{K_{m}^{\text{red},4}}+\frac{x_{5}}{K_{m}^{\text{red},5}}+\frac{x_{1}x_{2}}{K_{m}^{\text{red},12}}+\frac{x_{4}x_{5}}{K_{m}^{\text{red},45}}}. \end{array}$$

When the complex to be deleted in a reaction network is made up of more than one species, QSSA cannot be applied in some cases. Example 1 is one such case. With reference to this example, using equation (12), one needs to solve for *x*_3_ and *x*_4_ in order to apply QSSA. However, this is not possible as there is one equation and two unknowns *x*_3_ and *x*_4_ to be solved for. Thus our model reduction method is more effective in dealing with deletion of complexes made up of more than one species.

#### Effect of model reduction

In this section, we show the graph restructuring of our reduced model in terms of its corresponding full model for three particular types of linkage classes of biochemical reaction networks. Note that the deletion of a set of complexes in one linkage class does not affect the mathematical models of the reactions of the other linkage classes of the network.

Type 1 linkage class:

(16) $$\text{Full Network:}\;C_{1}⇌C_{2}⇌C_{3}⇌\cdots \cdots ⇌C_{n}$$

(17) $$\text{Reduced Network:}\;C_{1}⇌C_{3}⇌\cdots \cdots ⇌C_{n}$$

Consider a linkage class with reversible reactions occuring between consecutive elements of the set of distinct complexes $\left\{C_{1},C_{2},\ldots ,C_{n}\right\}$ as in (16). The reduced network obtained by deleting the complex $C_{2}$ is given by (17), where the two reversible reactions, $C_{1}⇌C_{2}$ and $C_{2}⇌C_{3}$ of the full network are replaced by a reversible reaction $C_{1}⇌C_{3}$ in the reduced network. If the kinetics of the reactions $C_{1}⇌C_{2}$ and $C_{2}⇌C_{3}$ are of the same type, the reaction $C_{1}⇌C_{3}$ of the reduced network has the same kinetics. This has been shown for a particular type of reactions in Example 1. The rate equations of all the remaining reactions of the reduced network are the same as those of the full network.

A special case of linkage class (16) is the following:

(18) $$C_{1}⇌C_{2}$$

Deletion of the complex $C_{2}$ in this case is equivalent to deletion of the linkage class from the network. Such a deletion provides a close approximation to the original network if the reaction (18) has very little effect on the dynamics of the network.

Type 2 linkage class:

(19) $$\text{Full Network 1:}\;\cdots \;C_{1}⇌C_{2}\to C_{3}\;\cdots$$

(20) $$\text{Reduced Network 1:}\;\cdots \;C_{1}\to C_{3}\;\cdots$$

(21) $$\text{Full Network 2:}\;\cdots \;C_{1}\to C_{2}⇌C_{3}\;\cdots$$

(22) $$\text{Reduced Network 2:}\;\cdots \;C_{1}\to C_{3}\;\cdots$$

In this type of a linkage class, we have one complex $C_{2}$ involved in both a reversible reaction and an irreversible reaction as shown in (19) and (21). The reversible and the irreversible reactions in which $C_{2}$ is involved in the full network are replaced with a single irreversible reaction in the reduced network as in (20) and (22). The deletion of $C_{2}$ does not affect the mathematical description of the remaining reactions of the linkage class. As with the previous type of linkage class, if the kinetics of the reversible and irreversible reactions of the full network are of the same type, the reaction $C_{1}\to C_{3}$ of the reduced network has the same kinetics.

**Type 3 linkage class:**

In this type of linkage classes, we have a complex that is involved in more than two reactions as the one shown in (23). In this example, if we delete the complex $C_{2}$, we arrive at the reduced network shown in (24). This deletion has no effect on the mathematical description of the remaining part of the network. Similar to the previous type of linkage classes, the reduced model of this type of a linkage class inherits the kinetics of the full model.

Notice that though the number of complexes in the reduced network is less than that of the original network, the number of reactions is the same in both. The reaction constants of reaction 4 of the reduced network depends on the reaction constants of reactions 1 and 2 of the full network; those of reaction 5 depend on those of reactions 1 and 3 and those of reaction 6 depend on those of reactions 2 and 3. Furthermore, deletion of the complex $C_{2}$ in this case does not lead to a reduction in the number of parameters.

In the next section, we show the application of our model reduction method on a yeast glycolysis model and a beta oxidation model in rat liver. In both these cases, we encounter linkage classes belonging to one of the three types mentioned above.

## Results and discussion

### Yeast Glycolysis model

**Figure 2:**
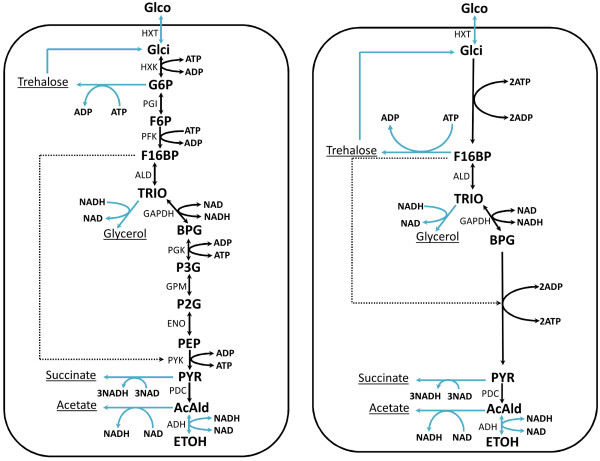
**Schematic of the original and reduced yeast-glycolysis networks.** The left-hand panel is a schematic representation of the yeast glycolysis model used for model reduction. The full model description and an explanation of all the abbreviations is found in [26]. The right-hand panel represents the reduced model after deleting 5 complexes (F6P, P2G, P3G, G6P and PEP). The blue arrows in the two panels indicate external fluxes.

We have applied our model reduction procedure on a detailed kinetic model of yeast glycolysis [26]. A schematic representation of the model is shown in the left-hand panel of Figure 2. The corresponding detailed mathematical model can be found in [26]. This network consists of type 1 and type 2 linkage classes.

For the modelling, we have considered the following as external fluxes as indicated in Figure 2: 

1. uptake of extracellular glucose (Glco) into the cell;

2. conversion of trehalose into intracellular glucose (Glci);

3. production of trehalose from glucose 6-phosphate (G6P);

4. production of glycerol from TRIO (TRIO is a pool summing up dihydroxyacetone phosphate and glyceraldehyde 3-phosphate);

5. production of succinate from pyruvate (PYR);

6. production of acetate from acetaldehyde (AcAld);

7. production of ethanol from AcAld.

**Figure 3:**
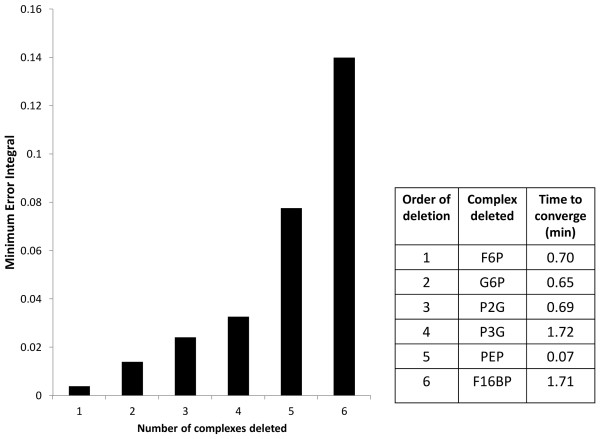
**Reduction of the yeast glycolysis model.** Left-hand panel: minimum error integral versus number of deleted complexes (we have taken *T*=1.5 min for the computation of the error integral). Right-hand panel: identity of the deleted complexes and their convergence times.

The model reduction procedure is applied to a glucose upshift as described in [26] and the corresponding parameter set was chosen [26]. Under these conditions, the imposed concentrations of Glco are 0.2 mM and 5 mM for *t*<0 and *t*≥0 respectively, and as observed experimentally the corresponding concentrations of ATP are equal to 5 mM and 2.5 mM. It is assumed that the network is at steady state for *t*<0. It is found that the model is asymptotically stable. The reactions of the network are governed by enzyme kinetics and we can write the equations of the model in the same form as equation (8). The set of significant species $\left({ℳ}_{I}\right)$ for the model consists of Glci, TRIO, BPG, PYR, AcAld and NADH. Figure 3 depicts the minimum error integrals as a function of the number of deleted complexes. Since deletion of a sixth complex leads to the error integral exceeding 0.1 in value, we stop the model reduction process after deletion of five complexes. The order of deletion is F6P, G6P, 2-phosphoglycerate (P2G), 3-phosphoglycerate (P3G) and phosphoenoylpyruvate (PEP). Deletion of P3G and P2G produces the same effect as the deletion of complex $C_{2}$ from a type 1 linkage class as described in the section on model reduction. Deletion of each of the remaining complexes produces the same effect as the deletion of complex $C_{2}$ from a type 2 linkage class. Each deletion results in the shortening of the chain length of the linkage class to which the complex belongs (see Figure 2, right-hand panel).

It is observed that there is a good agreement between the transient behaviours of most of the metabolites when comparing the original model to the reduced model with five complexes deleted. The main reason for this is that the reactions of the original model that are missing in the reduced one (see Figure 2) are the close-to-equilibrium reactions of the network. The concentration of F16BP has a strong effect on enzyme rates, both locally on the reactions in which it is involved, and distantly on the rate of pyruvate kinase (PYK). Together, this provides a clear explanation why F16BP ends up last in the order of complexes to be deleted (see Figure 3). The table of Figure 3 gives the convergence times of the six metabolites (complexes) that were considered for deletion. These are the times that it takes for the metabolite concentrations to achieve 95% of their concentration change going from one steady state to the other. Observe that the order of deletion of the complexes is not the same as the increasing order of their convergence times.

**Figure 4:**
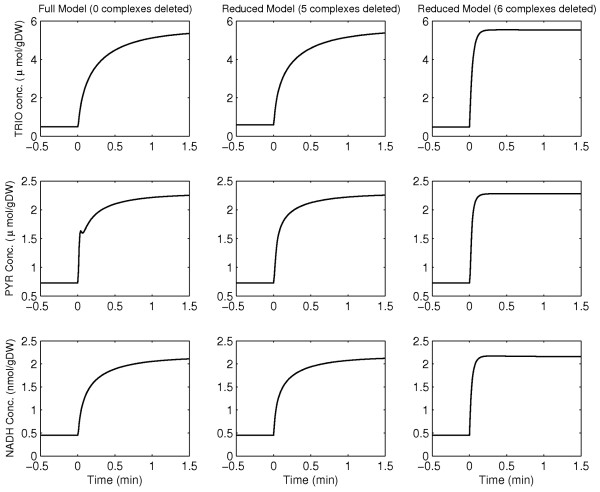
Comparison of concentration profiles of yeast-glycolysis metabolites between the full and reduced models.

While the original model has 12 variables, 88 parameters and 12 reactions, the reduced model has 7 variables, 50 parameters and 7 reactions. We have provided the Matlab files corresponding to the original model, the reduced model and automation of the model reduction procedure for the yeast glycolysis model, as additional files (see Additional files 2, 3 and 4 respectively). Additional file 4 gives as output the order of deletion of complexes and the error integral at each step of deletion. Additional files 5, 6, 7 and 8 are Matlab files that are required to run Additional file 4. We have submitted to Biomodels two SBML files corresponding to the original and the reduced yeast glycolysis models (submission identifiers MODEL1403250001 and MODEL1403250002 respectively). The evolution of the concentrations of Glci, PYR, TRIO, ACALD, BPG and NADH is compared between the different stages of model reduction in Figure 4.

### Fatty acid beta-oxidation model

**Figure 5:**
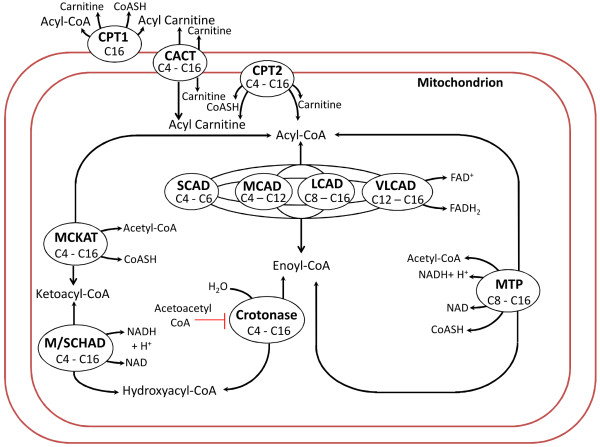
**Schematic of a model of beta-oxidation in rat liver.** The ellipses within, on and outside the boundary of the mitochondrion represent the enzymes responsible for the beta-oxidation. The full model description and explanation of all the abbreviation can be found in [27].

Subsequently, we have applied our model reduction method to a very different type of biochemical network, i.e., a model of fatty-acid beta-oxidation in rat liver [27]. This network mostly consists of type 1 and type 3 linkage classes. The model is shown schematically in Figure 5. Acyl carnitines of even chain lengths are broken down in the mitochondria to produce acetyl CoA. The acyl carnitines are first converted to acyl CoA’s of the same chain length within the mitochondria by the enzyme carnitine palmitoyl transferase II. Through a series of enzymatic reactions involving the enzymes acyl CoA dehydrogenase, crotonase (CROT), hydroxyacyl CoA dehydrogenase (M/SCHAD), *β*-ketothiolase (MCKAT) and mitochondrial trifunctional protein (MTP), the carbon chain length of each molecule of acyl CoA is shortened by 2 at the same time producing one molecule of acetyl CoA.

The carbon chain-lengths of the compounds processed by each of these enzymes is indicated within the ellipses. The full model has 42 state variables, 160 parameters and 56 reactions. The mathematical model is first written in the form (8). It is found that the model is asymptotically stable. At steady state, each enzyme has a constant reaction flux (rate).

By making use of the biochemical knowledge of the beta oxidation model, an initial reduction of the beta oxidation model has been performed as described below. This procedure holds only for this particular model and is not obtained by an automated deletion of complexes. It can be seen from Figure 5 that certain acyl CoAs having the same carbon chain lengths can be dehydrogenated by multiple enzymes, for example C8 acyl CoA can be dehydrogenated by three enzymes MCAD, LCAD or VLCAD. By checking the steady state fluxes of the same reaction through these different enzymes, we can reduce the model. For example, we found that the flux of dehydrogenation of C6 acyl CoA by MCAD is 99 times that by SCAD. Thus the model can be reduced by assuming that C6 acyl CoA is dehydrogenated by MCAD alone and not by SCAD. Similarly, we found that the model can be further reduced by assuming that C4 acyl CoA is dehydrogenated by SCAD alone and C8 acyl CoA is dehydrogenated by MCAD alone as the dehydrogenation fluxes of these compounds via other enzymes is comparatively very low.

Subsequently, we performed a further reduction of the beta oxidation model by deletion of certain complexes according to the procedure described earlier. It can be seen from Figure 5 that the shortening of chain length of acyl CoAs of chain lengths 16, 14, 12, 10 and 8 can happen via two routes, i.e. either via the enzyme MTP or via the sequence of enzymes crotonase, M/SCHAD and MCKAT. Deletion of the complexes hydroxyacyl and ketoacyl CoAs of even chain lengths between 8 and 16 is equivalent to having all acyl CoAs of chain lengths 8 till 16 reduced by only the first route. It is found that such a deletion produces a reduced model which has a very similar transient behaviour as the full model. The observation that the steady state fluxes through the first route is about a hundred times larger than the steady state fluxes through the second route explains the fact that removal of the second route does not have much effect on the dynamics of the rest of the system.

Note that the shortening of chain length of acyl CoAs of chain lengths 6 and 4 happens only via the sequence of enzymes crotonase, M/SCHAD and MCKAT. It is found that the combined effect of enzymes crotonase and M/SCHAD can be produced by a single fictitious enzyme which we have termed as CRMS. This is done by deleting the complexes C4 hydroxyacyl CoA and C6 hydroxyacyl CoA. Again such a deletion has a negligible effect on the dynamics of the rest of the system.

Each of the 12 deletions of complexes mentioned above produces the same effect as the deletion of the complex $C_{2}$ from a type 1 linkage class as described in the section on effect of model reduction. Deletion of hydroxyacyl and ketoacyl CoAs of even chain lengths between 16 and 8 is equivalent to removal of the linkage classes to which they belong. Deletion of C4 and C6 hydroxyacyl CoA is equivalent to reducing the number of reactions in the linkage class to which they belong, which in turn is equivalent to replacing the enzymes crotonase and M/SCHAD with a single enzyme CRMS.

**Figure 6:**
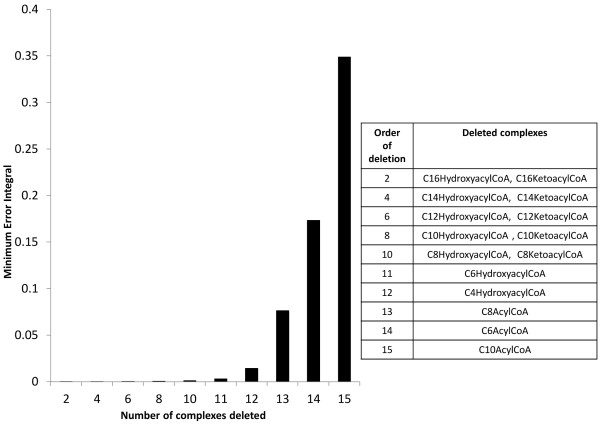
**Minimum error integral vs number of complexes deleted from the beta-oxidation model.** We have taken *T*=25 min for the computation of the error integral.

The set of significant species $\left({ℳ}_{I}\right)$ for the model consists of all the acyl carnitines in the cytoplasm and the mitochondria, since these have been measured for the original model validation [27]. Figure 6 depicts the values of minimum error integrals vs the number of complexes deleted in order to obtain a reduced model of beta oxidation. It can be seen that when the fourteenth complex is deleted from the model, the minimum error integral is more than 0.1. Therefore as discussed earlier, we stop our model reduction procedure after deletion of 13 complexes. The first 13 complexes that are deleted are the ones that were mentioned in the previous paragraphs in addition to C8 acyl CoA. Deletion of C8 acyl CoA produces the same effect as the deletion of the complex $C_{2}$ from a type 3 linkage class as described earlier. This deletion does not lead to a reduction in the number of reactions or parameters; only the number of complexes is reduced. In the following we show the local effect of deletion of C8 acyl CoA schematically. 

**Figure 7:**
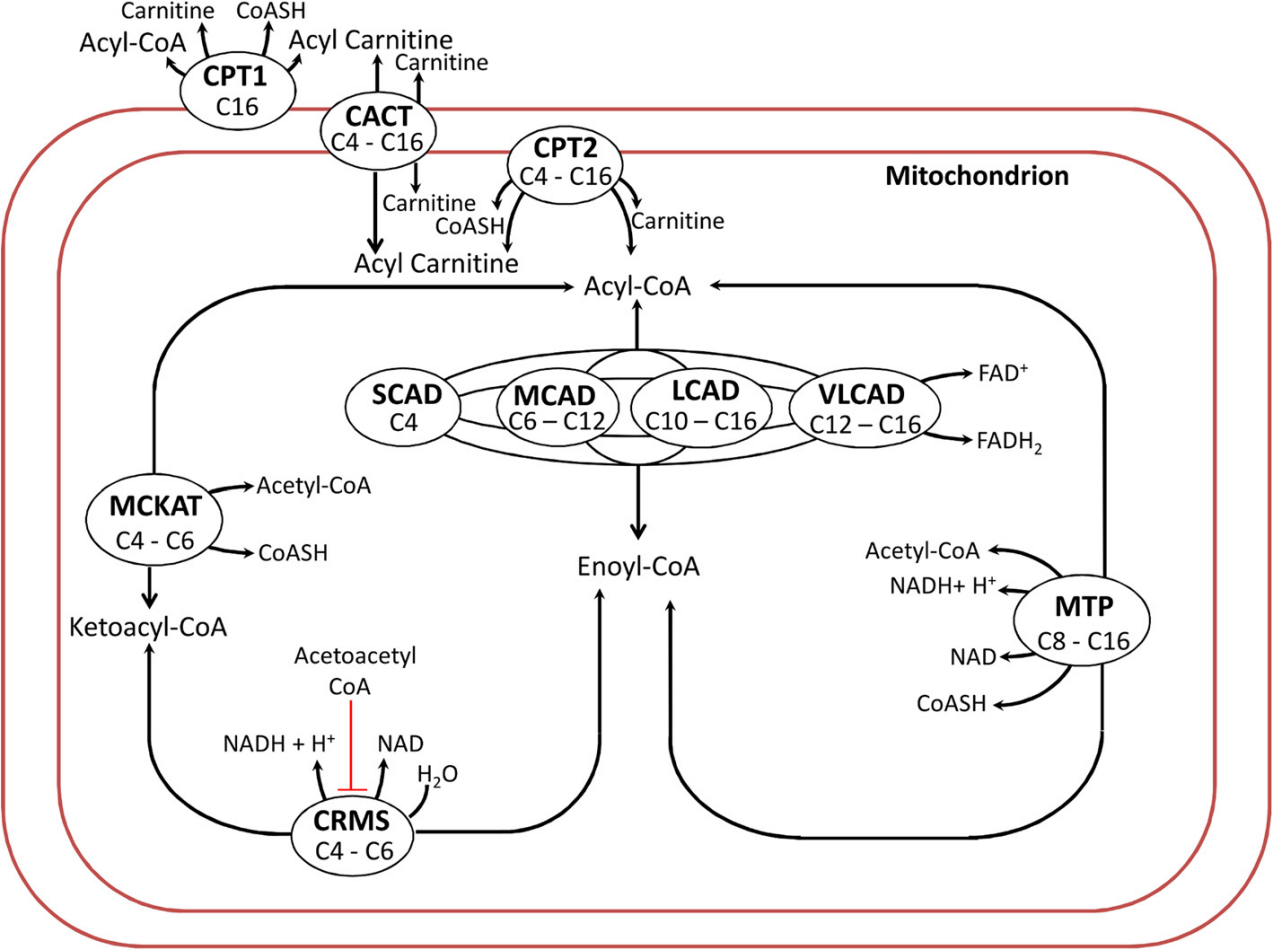
**Schematic of the reduced model (with 12 complexes deleted) of beta-oxidation in rat liver.** CRMS is a fictitious enzyme as described in the section “Fatty acid beta-oxidation model”.

**Figure 8:**
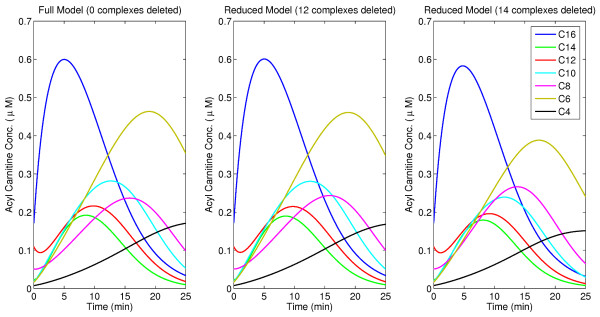
Comparison of concentration profiles of acyl carnitines between the full and reduced beta-oxidation models.

The reduced model obtained by incorporating all the deletions mentioned above except the deletion of the complex C8 acyl CoA is depicted in Figure 7. It has 31 state variables, 118 parameters and 37 reactions while the full model has 43 state variables, 160 parameters and 56 reactions. It is found that the transient behaviour of all the state variables of the reduced model with 12 complexes deleted are in good agreement with those obtained using the full model. Both the original and the reduced model have been provided as additional files (see Additional files 9 and 10 respectively). An SBML file corresponding to the reduced model has been submitted to Biomodels (submission identifier MODEL1403250000). Figure 8 depicts the comparison of the transient behaviours of the concentrations of all the acyl carnitines in the cytoplasm.

### Discussion

Our model reduction method involves rewriting of the complex graph corresponding to a given network. In the literature, there are other model reduction methods that involve graph rewriting like QSSA and quasi-equilibrium procedures, the method of [21] and the graph rewriting of monomolecular reaction networks described in [3]. The difference between these methods and ours is that these involve rewriting of the species-reactions graph unlike ours where the complex graph is rewritten.

In order to come up with a reasonably good reduced model, we follow an iterative procedure as described in the subsection titled “Error integral”. This iterative procedure involves computation of error integrals of a number of reduced models which are obtained by deletion of different combinations of complexes. In the beta-oxidation example, where the original model consists of 43 species and the reduced one has 30 species, the required number of simulations is (43-*M*)+(42-*M*)+(41-*M*)+⋯+(31-*M*) where *M* is the number of species that must still be present in the reduced model (and hence, are not considered in the subset of complexes for deletion). In our example, *M*=14 (corresponding to all Acyl-carnitines in the cytosol and mitochondria) and the total simulations using our procedure are 299. More precisely, if there are *N* number of species, *M* number of important species and if *L* is the dimension of the reduced species, then the total number of simulations is $\overset{N}{\underset{i=L}{\sum }}(i-M)=\left(\frac{L+N}{2}-M\right)(N-L+1)$. Although our procedure is time consuming, it is simple and systematic, ensures that the reduced model closely mimics the transients of the original model and is scalable to a large model. We are currently investigating the computation of error bounds based on the algebraic property of the Laplacian matrices and we foresee that this knowledge might allow us to directly obtain the optimal set of complexes to be deleted.

The cut-off value of the error integral at which we stop our iterative model reduction procedure can be set according to the desired closeness to the original model. For the two models discussed in this paper, this value has been set at 0.1 and it has been found that if this value is set at 0.2, then the resulting comparison plots of the transient behaviours of the original vs the reduced models reveal significant visual differences. Another method of coming up with a cut-off value for the error integral is to look for a sudden, steep increase in the error integral histogram. However in the case where the error-integral histogram shows uniform increase with an increase in the number of complexes deleted, this method cannot be used.

Note that in principle, complexes can be deleted to reduce any biochemical model, whether asymptotically stable or not. However, for the computation of error integral as described in the paper, it is necessary that the original network is asymptotically stable around a steady state, since the computation makes use of the steady state concentrations of some species of the network.

In some cases, deletion of certain complexes can drastically change the dynamics of the system. An example is the following. Assume that *X*_1_,*X*_2_,…,*X*_10_ are distinct species of the network. Consider the following reaction network consisting of three reversible reactions:

$$\begin{array}{lll} & X_{1}+X_{2}⇌X_{3}+X_{4}\; & \;\\ & X_{4}+X_{5}⇌X_{6}+X_{7}\; & \;\\ & X_{7}+X_{8}⇌X_{9}+X_{10}\; & \; \end{array}$$

Now consider deletion of the complex *X*_4_+*X*_5_ from the above reaction network. This deletion leads to the deletion of the second reaction of the network. Since the first and the third reaction of the network do not have common species, deletion of the second reaction leads to the first and the third reactions occuring independently of each other. This will lead to the reduced model exhibiting a behaviour which is not close to the behaviour of the original model. Although one can quantify the difference between the behaviours using error integrals, avoiding such deletions will reduce the computational effort in making the choice of complexes to be deleted. Examples of such deletions are deletion of complexes TRIO or BPG in the yeast glycolysis model and deletion of complex C16 Acyl Carnitine in the beta oxidation model.

We remark here that one should update a reduced model when major changes are made to the original model. For example, if we consider the rat liver beta-oxidation model of a MCAD-deficient animal, this model will not have the enzyme MCAD. In this case, the dynamic behaviour of the reduced model of Figure 7 may be far from that of the original model of Figure 5 without the enzyme MCAD.

## Conclusions

In this paper, we have outlined a method for model reduction of biochemical reaction networks that are asymptotically stable around a steady state. The principle behind our model reduction method is to couple the dynamics of certain complexes to the dynamics of the neighbouring complexes in such a way that the net rate of inflow into the complex is equal to the net rate of outflow from it. We provide an algorithm for choosing the complexes to be deleted in such a way that the transient behaviour of the significant species of the reduced model is close to that of the original model. Apart from a reduction in the number of state variables, our model reduction method also leads to a reduction in the number of reactions and parameters of the model. This in turn leads to an improved computational effort required in order to analyze the model. Our model reduction procedure ensures that the reduced model mimics the full model well under the conditions of a specific dynamic event. A different reduced model with a different set of deleted complexes may be produced by our procedure under the conditions of a different dynamic event.

In Additional file 1, we give a list of some well-known enzyme kinetic rate laws for which our model reduction method is applicable. Some of the rate laws provided in this list are rate laws for irreversible reactions. However our method is also applicable for the reversible version of these rate laws. This is because a reversible reaction can be split into its forward and reverse reaction components and if our method is applicable to each of these components then it is applicable to the given reversible reaction. For example, since our method is applicable to reactions governed by irreversible mass action kinetics, it is also applicable for reactions governed by reversible mass action kinetics. The list provided in Additional file 1 is not an exhaustive list of all enzyme kinetic rate laws for which our method is applicable. We have checked that our method is applicable to all the rate laws that are included in the COPASI software [34].

We have applied our method on a yeast glycolysis model and a beta-oxidation model in rat liver and have observed a good agreement between the transient behaviours of the original and the reduced models. In these cases, our model reduction method has also helped us significantly in improving our understanding of the dynamics of the networks, for example by identification of the close-to-equilibrium reactions of the yeast glycolysis model and of the reaction pathways with comparatively low fluxes in the rat liver beta-oxidation model. In the case of the yeast glycolysis model, we found that the order of deletion of complexes (metabolites) is not the same as the order of their convergence times. This shows that in this case, our model reduction approach will perform differently from a time-scale separation technique where metabolites with faster convergence times are assumed to attain rapid steady state. As observed in the two examples of metabolic reaction networks, our model reduction method helps in removing redundancies in the given network (in the yeast glycolysis network, redundant reactions were coupled and in the rat liver beta-oxidation model, redundant pathways were removed).

Recently, there has been an interest in kinetic modelling of large genome-scale networks. For example, [35] gives a method for building a parameterized genome-scale kinetic model of a network consisting of 956 reactions and 820 metabolites. We envisage that our model reduction method will be particularly useful to reduce the complexity of such large genome-scale kinetic models in the future. Likewise, it can also be useful in reducing multiscale models of biochemical reaction networks which are computationally burdensome to analyze. In a large-scale kinetic model of a biochemical reaction network, our model reduction method can be used to reduce all pathways except the ones of interest into which we would like to zoom-in and analyze.

Currently, we are using our reduced model of the rat liver beta-oxidation model that we explained in an earlier section in order to check for possible bifurcations. In this respect, the reduced model provides an advantage over the full model as it requires lesser computational effort while checking for bifurcation.

## Ethics

The authors declare that no experiments have been performed as part of the research for this manuscript.

## Competing interests

The authors declare that they have no competing interests.

## Authors’ contributions

AJvdS, SR and BJ conceived the idea of model reduction. AJvdS prepared a first draft of some of the subsections under the “Methods” section. BMB conceived the idea of stepwise automated reduction procedure. KvE provided the original models of yeast glycolysis and rat liver beta oxidation. SR and BJ carried out reduction of these models. SR prepared the manuscript with the help of BMB and BJ. All authors read and approved the final manuscript.

## Supplementary Material

Additional file 1 Enzyme kinetic rate laws for which our model reduction method is applicable. Click here for file

Additional file 2 Original yeast glycolysis model. Click here for file

Additional file 3 Reduced yeast glycolysis model. Click here for file

Additional file 4 Automation of our model reduction procedure for the yeast glycolysis model. Click here for file

Additional file 5 Reduced yeast glycolysis model with a prespecified number of deleted complexes. Click here for file

Additional file 6 **A Matlab file required to run Additional file **4**.** Click here for file

Additional file 7 A Matlab function for computing the final steady state with given initial conditions. Click here for file

Additional file 8 A program to compute the error integral between the original and a given reduced model of the yeast glycolysis. Click here for file

Additional file 9 Original model of rat liver beta oxidation. Click here for file

Additional file 10 Reduced model of rat liver beta oxidation. Click here for file
