# Supplementary material for: A model reduction method for biochemical reaction networks
Source: BMC Syst Biol. 2014 May 3;8:52. doi: 10.1186/1752-0509-8-52 (PMC4041147; doi:10.1186/1752-0509-8-52)
Supplement: Additional file 3 — Reduced yeast glycolysis model. [file 1752-0509-8-52-S3.pdf]

Automation of our model reduction procedure for the yeast glycolysis model

```
indexing = [2,3,4,9,10,11]; %indexing of complexes considered for deletion
indexingmet = [2,3,4,7,8,9]; %indexing of corresponding species
x = [0.1, 1.2, 0.2, 0.25, 1, 0.00001, 0.54, 0.06, 0.5, 0.14, 0.04, 0.29,
1.3]; %random initial values to compute the initial steady state
[t,y] = ode15s(@FullmodelGF,-100:0.1:-1,x);
x1 = y(end,:)'; %initial steady state
clear t;
[t,y1] = ode15s(@FullmodelGF,-1:0.1:100,x1); %full model trajectory
x2 = y1(end,:)';
[xf,Ssq,CNT] = LMFsolve(@FullmodelGF1,x2); %xf is the final steady state,
FullmodelGF1 gives the fluxes as outputs and takes state vector as the
input for any time t>0
global red; %red is the set of complexes in the reduced model
global del; %del is the set of missing complexes in the reduced model
I = 0;
l = 6;
delfull = [];
x3 = x1;
EI = zeros(6,1);
[t,y1] = ode15s(@FullmodelGF,-0.015:0.0015:1.5,x1); %full model trajectory
with interval of interest [-0.015,1.5] minutes.

while I<0.1 %0.1 is the maximum error integral
    q = 1;
    E = zeros(l,1);
    for p = 1:l
        h = indexing(p);
        w = indexingmet(p);
        del = [delfull,h];
        red = 1:13;
        red(del) = [];
        x4 = x3;
        x4(w) = xf(w); %the initial values of the deleted species is set at
its final steady state value computed using the original model
        [t,y2] = ode15s(@newmodelGF,-0.015:0.0015:1.5,x4);
        E(p) = error_integral(y1,y2);
        if E(p) < q % to find the minimum error integral and the
corresponding behaviour (ygood) among those of reduced models with pth
complex deleted
            I = E(p); % minimum error integral at this step
            m = p; % index of corresponding complex
            q = I; % error integral for comparison in the next step
            ygood = y2;
        end
    end

    if I<0.1
        EI(l) = I; % minimum error integral with 6-l+1 complexes deleted
        l = l-1;
        x3(indexingmet(m)) = xf(indexingmet(m)); % initial value of the
species corresponding to the deleted complex set at its final steady state
value computed using the original mosel
        delfull = [delfull,indexing(m)];
        indexing(m)=[];
        indexingmet(m) = [];
```

```
        end
    end
```
