# Supplementary material for: A model reduction method for biochemical reaction networks
Source: BMC Syst Biol. 2014 May 3;8:52. doi: 10.1186/1752-0509-8-52 (PMC4041147; doi:10.1186/1752-0509-8-52)
Supplement: Additional file 5 — Reduced yeast glycolysis model with a prespecified number of deleted complexes. [file 1752-0509-8-52-S5.pdf]

A Matlab file required to run Additional file 3

```
function [output] = FullmodelGF1(input)
    statevector = input;

%%%%%%%%%%%%%%%%%%%%%%%%%%%%%%%%%%%%%%%%%%%%%%%
% STATES
%%%%%%%%%%%%%%%%%%%%%%%%%%%%%%%%%%%%%%%%%%%%%%%
Glci = statevector(1);
G6P = statevector(2);
F6P = statevector(3);
F16P = statevector(4);
TRIO = statevector(5);
BPG = statevector(6);
P3G = statevector(7);
P2G = statevector(8);
PEP = statevector(9);
PYR = statevector(10);
ACALD = statevector(11);
NADH = statevector(12);
NAD = statevector(13);

%%%%%%%%%%%%%%%%%%%%%%%%%%%%%%%%%%%%%%%%%%%%%%%
% PARAMETERS
%%%%%%%%%%%%%%%%%%%%%%%%%%%%%%%%%%%%%%%%%%%%%%%
Glco = 5;
ATP = 1.5;
ETOH = 25;
NADt = 1.59;

Keq_glt = 1;

Km_hk_glci = 0.08;
Km_hk_g6p = 30;
Km_hk_atp = 0.15;
Km_hk_adp = 0.23;
Ki_hk_t6p = 0.04;
Keq_hk = 3800;

Km_pgi_g6p = 1.4;
Km_pgi_f6p = 0.3;
Keq_pgi = 0.314;

gR_pfk = 5.12;
L0_pfk = 0.66;
Km_pfk_f6p = 0.1;
Km_pfk_atp = 0.71;
C_atp = 3;
Ci_pfk_atp = 100;
Ci_pfk_amp = 0.0845;
Ci_pfk_f16p = 0.397;
Ci_pfk_f26p = 0.0174;
Kpfk_atp = 0.65;
```

```
Kpfk_amp = 0.0995;
Kpfk_f16p = 0.111;
Kpfk_f26p = 0.000682;

Km_ald_f16p = 0.3;
Km_ald_dhap = 2.4;
Km_ald_gap = 10;
Keq_ald = 0.069;
Keq_tpi = 0.045;

Km_pgk_bpg = 0.003;
Km_pgk_p3g = 0.53;
Km_pgk_adp = 0.2;
Km_pgk_atp = 0.3;
Keq_pgk = 3200;

Km_gpm_p3g = 1.2;
Km_gpm_p2g = 0.08;
Keq_gpm = 0.19;

Km_eno_p2g = 0.04;
Km_eno_pep = 0.5;
Keq_eno = 6.7;

Km_pyk_pep = 0.19;
Km_pyk_adp = 0.3;
Km_pyk_atp = 9.3;
n_pyk = 4;
L0_pyk = 60000;
Km_pyk_f16p = 0.2;

Km_pdc_pyr = 6.36;
NH_pdc = 1.9;

Km_adh_acald = 1.11;
Km_adh_etoh = 17;
Km_adh_nadh = 0.11;
Km_adh_nad = 0.17;
Ki_adh_acald = 1.1;
Ki_adh_etoh = 90;
Ki_adh_nadh = 0.031;
Ki_adh_nad = 0.92;
Keq_adh = 6.9e-5;


Vmax_glt = 160;
Km_glt_glco = 1;
Km_glt_glci = 1;
Ki = 0.75;

Vmax_hk = 213;
Vmax_pgi = 787;
Vmax_pfk = 213;
Vmax_ald = 310;
```

```
Vmax_gapdh_f = 1487;
Vmax_gapdh_r = 853;
Km_gapdh_gap = 0.39;
Km_gapdh_nad = 2.85;
Km_gapdh_nadh = 0.007;
Km_gapdh_bpg = 0.51;

Vmax_pgk_r = 2512;
Vmax_gpm = 856;
Vmax_eno = 357;
Vmax_pyk = 820;
Vmax_pdc = 395;
Vmax_adh_r = 932;

K_tre1 = 0;
K_tre2 = 0;
K_gly = 17.5;
K_suc = 0.9;
K_ace = 0.5;

ADP = 1;
AMP = 0.3;
T6P = 0.2;
F26P = 0.014;

NAD = NADt - NADH;

Zmatrix = [1 0 0 0 0 0 0 0 0 0 0 0 0;... %From complex GLci to species GLci
           0 1 0 0 0 0 0 0 0 0 0 0 0;... %From complex G6P to species G6P
           0 0 1 0 0 0 0 0 0 0 0 0 0;... %From complex F6P to species F6P
           0 0 0 1 0 0 0 0 0 0 0 0 0;... %From complex F16P to species F16P
           0 0 0 0 2 1 0 0 0 0 0 0 0;... %From complex 2TRIO and TRIO+NAD to
species TRIO
           0 0 0 0 0 0 1 1 0 0 0 0 0;... %From complex BPG+NADH and BPG to
species BPG
           0 0 0 0 0 0 0 0 1 0 0 0 0;... %From complex P3G to species P3G
           0 0 0 0 0 0 0 0 0 1 0 0 0;... %From complex P2G to species P2G
           0 0 0 0 0 0 0 0 0 0 1 0 0;... %From complex PEP to species PEP
           0 0 0 0 0 0 0 0 0 0 0 1 0;... %From complex PYR to species PYR
           0 0 0 0 0 0 0 0 0 0 0 0 1;... % From complex ACALD to species
ACALD
           0 0 0 0 0 0 1 0 0 0 0 0 0;... %From complex BPG+NADH to species
NADH
           0 0 0 0 0 1 0 0 0 0 0 0 0]; %From complex TRIO+NAD to species NAD

V_glt_massaction = (Glco/Km_glt_glco - Glci/(Km_glt_glco*Keq_glt));
V_glt_common =
Vmax_glt/(1+Glco/Km_glt_glco+Glci/Km_glt_glci+Ki*Glco*Glci/(Km_glt_glco*Km_gl
t_glci));

V_hk_common =
Vmax_hk/(Km_hk_glci*Km_hk_atp*(1+Glci/Km_hk_glci+G6P/Km_hk_g6p+T6P/Ki_hk_t6p)
*(1+ATP/Km_hk_atp+ADP/Km_hk_adp));

V_pgi_common = Vmax_pgi/(Km_pgi_g6p*(1+G6P/Km_pgi_g6p+F6P/Km_pgi_f6p));
```

```
L_pfk =
L0_pfk*((1+Ci_pfk_atp*ATP/Kpfk_atp)/(1+ATP/Kpfk_atp))^2*((1+Ci_pfk_amp*AMP/Kp
fk_amp)/(1+AMP/Kpfk_amp))^2*((1+Ci_pfk_f26p*F26P/Kpfk_f26p+Ci_pfk_f16p*F16P/K
pfk_f16p)/(1+F26P/Kpfk_f26p+F16P/Kpfk_f16p))^2;
V_pfk_common =
Vmax_pfk*gR_pfk*(1+F6P*ATP/(Km_pfk_f6p*Km_pfk_atp)+gR_pfk*F6P*ATP/(Km_pfk_f6p
*Km_pfk_atp))/(Km_pfk_f6p*Km_pfk_atp*((1+F6P*ATP/(Km_pfk_f6p*Km_pfk_atp)+gR_p
fk*F6P*ATP/(Km_pfk_f6p*Km_pfk_atp))^2+L_pfk*(1+C_atp*ATP/Km_pfk_atp)^2));

V_ald_common =
Vmax_ald/(Km_ald_f16p*(1+F16P/Km_ald_f16p+(Keq_tpi*TRIO/(1+Keq_tpi))/Km_ald_g
ap+TRIO/((1+Keq_tpi)*Km_ald_dhap)+(Keq_tpi*(TRIO/(1+Keq_tpi))^2)/(Km_ald_gap*
Km_ald_dhap)+F16P*(Keq_tpi*(TRIO/(1+Keq_tpi)))/(Km_ald_gap*Km_ald_f16p)));

V_gapdh_common =
1/((1+Keq_tpi*TRIO/((1+Keq_tpi)*Km_gapdh_gap)+BPG/Km_gapdh_bpg)*(1+NAD/Km_gap
dh_nad+NADH/Km_gapdh_nadh));

V_pgk_common =
Vmax_pgk_r/(Km_pgk_p3g*Km_pgk_atp*(1+BPG/Km_pgk_bpg+P3G/Km_pgk_p3g)*(1+ATP/Km
_pgk_atp+ADP/Km_pgk_adp));

V_gpm_common = Vmax_gpm/(Km_gpm_p3g*(1+P3G/Km_gpm_p3g+P2G/Km_gpm_p2g));

V_eno_common = Vmax_eno/(Km_eno_p2g*(1+P2G/Km_eno_p2g+PEP/Km_eno_pep));

V_pyk_common = Vmax_pyk*(PEP/Km_pyk_pep+1)^(n_pyk-
1)/(L0_pyk*((ATP/Km_pyk_atp+1)/(F16P/Km_pyk_f16p+1))^n_pyk+(1+PEP/Km_pyk_pep)
^n_pyk)*ADP/(ADP+Km_pyk_adp);

V_pdc_common = Vmax_pdc*PYR^(NH_pdc-1)/((1+PYR^NH_pdc/Km_pdc_pyr^NH_pdc));

%%%%%%%%%%%%%%%%%%%%%%%%%%%%%%%%%%%%%%%%%%%%%%%
% Weighted Laplacian
%%%%%%%%%%%%%%%%%%%%%%%%%%%%%%%%%%%%%%%%%%%%%%%
L = zeros(13,13);
L(2,1) = -V_hk_common*ATP; %HK F
L(1,1) = -L(2,1);
L(1,2) = -V_hk_common*ADP/Keq_hk;  %HK R
L(3,2) = -V_pgi_common;  %PGI F
L(2,2) = -L(1,2)-L(3,2);
L(2,3) = -V_pgi_common/Keq_pgi; % PGI R
L(4,3) = -V_pfk_common*ATP;   %PFK
L(3,3) = -L(2,3)-L(4,3);
L(5,4) = -V_ald_common;   % ALD F
L(4,4) = -L(5,4);
L(4,5) = -V_ald_common*Keq_tpi/(Keq_ald*((1+Keq_tpi)^2));   %ALD R
L(5,5) = -L(4,5);
L(7,6) = -
V_gapdh_common*Vmax_gapdh_f*Keq_tpi/(1+Keq_tpi)/(Km_gapdh_gap*Km_gapdh_nad);
%GAPDH F
L(6,6) = -L(7,6);
L(6,7) = -V_gapdh_common*Vmax_gapdh_r/(Km_gapdh_bpg*Km_gapdh_nadh);    %
GAPDH R
```

```
L(7,7) = -L(6,7);
L(9,8) = -V_pgk_common*Keq_pgk*ADP;   % PGK F
L(8,8) = -L(9,8);
L(8,9) = -V_pgk_common*ATP;    % PGK R
L(10,9) = -V_gpm_common;    % GPM F
L(9,9) = -L(8,9)-L(10,9);
L(9,10) = -V_gpm_common/Keq_gpm;   %  GPM R
L(11,10) = -V_eno_common;    % ENO F
L(10,10) = -L(9,10)-L(11,10);
L(10,11) = -V_eno_common/Keq_eno;   %  ENO R
L(12,11) = -V_pyk_common/Km_pyk_pep;  % PYK
L(11,11) = -L(10,11)-L(12,11);
L(13,12) = -V_pdc_common/Km_pdc_pyr;  % PDC
L(12,12) = -L(13,12);

V_suc = K_suc;
V_ace = K_ace*ACALD;
V_tre1 = K_tre1;
V_tre2 = K_tre2;
V_gly = K_gly;

V_adh_massaction = (NAD*ETOH/(Ki_adh_nad*Km_adh_etoh)-
NADH*ACALD/(Ki_adh_nad*Km_adh_etoh*Keq_adh));
V_adh_common =
Vmax_adh_r/(1+NAD/Ki_adh_nad+Km_adh_nad*ETOH/(Ki_adh_nad*Km_adh_etoh)+Km_adh_
nadh*ACALD/(Ki_adh_nad*Km_adh_acald)+NADH/Ki_adh_nadh+NAD*ETOH/(Ki_adh_nad*Km
_adh_etoh)+Km_adh_nadh*NAD*ACALD/(Ki_adh_nad*Ki_adh_nadh*Km_adh_acald)+Km_adh
_nad*NADH*ETOH/(Ki_adh_nad*Ki_adh_nadh*Km_adh_etoh)+NADH*ACALD/(Ki_adh_nadh*K
m_adh_acald)+NAD*ETOH*ACALD/(Ki_adh_nad*Km_adh_etoh*Ki_adh_acald)+NADH*ETOH*A
CALD/(Ki_adh_etoh*Ki_adh_nadh*Km_adh_acald));
V_adh = V_adh_massaction*V_adh_common;

ExternalReactionrate = [V_glt_massaction*V_glt_common+2*V_tre1;-
2*V_tre2;0;0;-V_gly;0;0;0;0;-2*V_suc;-V_ace+V_adh;-
V_gly+V_adh+3*V_suc+V_ace;V_gly-V_adh-3*V_suc-V_ace;];
Reactionrate = -1*Zmatrix*L*exp(Zmatrix'*log(statevector));

Reactionrate = Reactionrate + ExternalReactionrate;

output = Reactionrate;
return
```
