# Supplementary material for: A model reduction method for biochemical reaction networks
Source: BMC Syst Biol. 2014 May 3;8:52. doi: 10.1186/1752-0509-8-52 (PMC4041147; doi:10.1186/1752-0509-8-52)
Supplement: Additional file 6 — A Matlab file required to run Additional file 4. [file 1752-0509-8-52-S6.pdf]

# A Matlab function for computing the final steady state with given initial conditions

```
function [xf, S, cnt] = LMFsolve(varargin)
% LMFSOLVE  Solve a Set of Nonlinear Equations in Least-Squares Sense.
%%%%%%%%%%%%%%%%%%%%%%%%%%%%%%%%%%%%%%%%%%%%%%%%%%%%%%%%%%%%%%%%%%%%%%%%%
% A solution is obtained by a shortened Fletcher version of the
% Levenberg-Maquardt algoritm for minimization of a sum of squares
%  of equation residuals.
%
% [Xf, Ssq, CNT] = LMFsolve(FUN,Xo,Options)
% FUN     is a function handle or a function M-file name that evaluates
%         m-vector of equation residuals,
% Xo      is n-vector of initial guesses of solution,
% Options is an optional set of Name/Value pairs of control parameters
%         of the algorithm. It may be also preset by calling:
%         Options = LMFsolve('default'), or by a set of Name/Value pairs:
%         Options = LMFsolve('Name',Value, ... ), or updating the Options
%                   set by calling
%         Options = LMFsolve(Options,'Name',Value, ...).
%
%    Name   Values {default}         Description
% 'Display'     integer     Display iteration information
%                            {0}  no display
%                             k   display initial and every k-th iteration;
% 'FunTol'      {1e-7}      norm(FUN(x),1) stopping tolerance;
% 'XTol'        {1e-7}      norm(x-xold,1) stopping tolerance;
% 'MaxIter'     {100}       Maximum number of iterations;
% 'ScaleD'                  Scale control:
%               value        D = eye(m)*value;
%               vector       D = diag(vector);
%                {[]}        D(k,k) = JJ(k,k) for JJ(k,k)>0, or
%                                   = 1 otherwise,
%                                     where JJ = J.'*J
% Not defined fields of the Options structure are filled by default values.
%
% Output Arguments:
% Xf        final solution approximation
% Ssq       sum of squares of residuals
% Cnt       >0          count of iterations
%           -MaxIter,   did not converge in MaxIter iterations

% Example:  Rosenbrock valey inside circle with unit diameter
%   R  = @(x) sqrt(x'*x)-.5;    %   A distance from the radius r=0.5
%   ros= @(x) [ 10*(x(2)-x(1)^2); 1-x(1); (R(x)>0)*R(x)*1000];
%   [x,ssq,cnt]=LMFsolve(ros,[-1.2,1],'Display',1,'MaxIter',50)
% returns   x = [0.4556; 0.2059],  ssq = 0.2966,  cnt = 18.
%
% Note:   Users with old MATLAB versions (<7), which have no anonymous
% functions implemented, should call LMFsolve with named function for
% residuals. For above example it is
%   [x,ssq,cnt]=LMFsolve('rosen',[-1.2,1]);
% where the function rosen.m is of the form
```

```matlab
%   function r = rosen(x)
%%   Rosenbrock valey with a constraint
%   R = sqrt(x(1)^2+x(2)^2)-.5;
%%   Residuals:
%   r = [ 10*(x(2)-x(1)^2)  %   first part
%         1-x(1)            %   second part
%         (R>0)*R*1000.     %   penalty
%       ];

% Reference:
% Fletcher, R., (1971): A Modified Marquardt Subroutine for Nonlinear Least
% Squares. Rpt. AERE-R 6799, Harwell

% Miroslav Balda,
% balda AT cdm DOT cas DOT cz
% 2007-07-02    v 1.0
% 2008-12-22    v 1.1 * Changed name of the function in LMFsolv
%                     * Removed part with wrong code for use of analytical
%                       form for assembling of Jacobian matrix
% 2009-01-08    v 1.2 * Changed subfunction printit.m for better one, and
%                       modified its calling from inside LMFsolve.
%                     * Repaired a bug, which caused an inclination to
%                       istability, in charge of slower convergence.
%%%%%%%%%%%%%%%%%%%%%%%%%%%%%%%%%%%%%%%%%%%%%%%%%%%%%%%%%%%%%%%%%%%%%%%%%%%%%%%%%%%%%%%%%%%%%%%%%%%%%%%%%%%%%%%%%%%%%%

%   OPTIONS
    %%%%%%%
%               Default Options
if nargin==1 && strcmpi('default',varargin(1))
   xf.Display  = 0;         %   no print of iterations
   xf.MaxIter  = 100;       %   maximum number of iterations allowed
   xf.ScaleD   = [];        %   automatic scaling by D = diag(diag(J'*J))
   xf.FunTol   = 1e-7;      %   tolerance for final function value
   xf.XTol     = 1e-4;      %   tolerance on difference of x-solutions
   return

%               Updating Options
elseif isstruct(varargin{1}) % Options=LMFsolve(Options,'Name','Value',...)
    if ~isfield(varargin{1},'Display')
        error('Options Structure not correct for LMFsolve.')
    end
    xf=varargin{1};          %   Options
    for i=2:2:nargin-1
        name=varargin{i};    %   Option to be updated
        if ~ischar(name)
            error('Parameter Names Must be Strings.')
        end
        name=lower(name(isletter(name)));
        value=varargin{i+1}; %   value of the option
        if strncmp(name,'d',1), xf.Display  = value;
        elseif strncmp(name,'f',1), xf.FunTol   = value(1);
        elseif strncmp(name,'x',1), xf.XTol     = value(1);
        elseif strncmp(name,'m',1), xf.MaxIter  = value(1);
        elseif strncmp(name,'s',1), xf.ScaleD   = value;
        else   disp(['Unknown Parameter Name --> ' name])
        end
```

```
    end
    return

%               Pairs of Options
elseif ischar(varargin{1})   % check for Options=LMFSOLVE('Name',Value,...)
   Pnames=char('display','funtol','xtol','maxiter','scaled');
   if strncmpi(varargin{1},Pnames,length(varargin{1}))
      xf=LMFsolve('default'); % get default values
      xf=LMFsolve(xf,varargin{:});
      return
   end
end

%   LMFSOLVE(FUN,Xo,Options)
    %%%%%%%%%%%%%%%%%%%%%%%%%%%

FUN=varargin{1};            %   function handle
if ~(isvarname(FUN) || isa(FUN,'function_handle'))
   error('FUN Must be a Function Handle or M-file Name.')
end

xc=varargin{2};             %   Xo

if nargin>2                 %   OPTIONS
    if isstruct(varargin{3})
        options=varargin{3};
    else
        if ~exist('options','var')
            options = LMFsolve('default');
        end
        for i=3:2:size(varargin,2)-1
            options=LMFsolve(options, varargin{i},varargin{i+1});
        end
    end
else
    if ~exist('options','var')
        options = LMFsolve('default');
    end
end

x  = xc(:);
lx = length(x);

r  = feval(FUN,x);             % Residuals at starting point
%~~~~~~~~~~~~~~~~~
S  = r'*r;
epsx = options.XTol(:);
epsf = options.FunTol(:);
if length(epsx)<lx, epsx=epsx*ones(lx,1); end
J = finjac(FUN,r,x,epsx);
%~~~~~~~~~~~~~~~~~~~~~~~
nfJ = 2;
A = J.'*J;                    % System matrix
v = J.'*r;
```

```
D = options.ScaleD;
if isempty(D)
    D = diag(diag(A));        % automatic scaling
    for i = 1:lx
        if D(i,i)==0, D(i,i)=1; end
    end
else
    if numel(D)>1
        D = diag(sqrt(abs(D(1:lx)))); % vector of individual scaling
    else
        D = sqrt(abs(D))*eye(lx);     % scalar of unique scaling
    end
end

Rlo = 0.25;
Rhi = 0.75;
l=1;      lc=.75;     is=0;
cnt = 0;
ipr = options.Display;
printit(ipr,-1);                %   Table header
d = options.XTol;               %   vector for the first cycle
maxit = options.MaxIter;        %   maximum permitted number of iterations

while cnt<maxit && ...          %   MAIN ITERATION CYCLE
    any(abs(d) >= epsx) && ...      %%%%%%%%%%%%%%%%%%%%%%%%%%%
    any(abs(r) >= epsf)
    d  = (A+l*D)\v;             %   negative solution increment
    xd = x-d;
    rd = feval(FUN,xd);
%   ~~~~~~~~~~~~~~~~~~~
    nfJ = nfJ+1;
    Sd = rd.'*rd;
    dS = d.'*(2*v-A*d);         %   predicted reduction

    R  = (S-Sd)/dS;
    if R>Rhi                    %   halve lambda if R too high
        l = l/2;
        if l<lc, l=0; end
    elseif R<Rlo                %   find new nu if R too low
        nu = (Sd-S)/(d.'*v)+2;
        if nu<2
            nu = 2;
        elseif nu>10
            nu = 10;
        end
        if l==0
            lc = 1/max(abs(diag(inv(A))));
            l  = lc;
            nu = nu/2;
        end
        l = nu*l;
    end

    cnt = cnt+1;
    if ipr~=0 && (rem(cnt,ipr)==0 || cnt==1) %   print iteration?
        printit(ipr,cnt,nfJ,S,x,d,l,lc)
```

```
    end

    if Sd<S
        S = Sd;
        x = xd;
        r = rd;
        J = finjac(FUN,r,x,epsx);
%       ~~~~~~~~~~~~~~~~~~~~~~~~~
        nfJ = nfJ+1;
        A = J'*J;
        v = J'*r;
    end
end %   while

xf = x;                         %   final solution
if cnt==maxit
    cnt = -cnt;
end                             %   maxit reached
rd = feval(FUN,xf);
nfJ = nfJ+1;
Sd = rd.'*rd;
if ipr, disp(' '), end
printit(ipr,cnt,nfJ,Sd,xf,d,l,lc)

%%%%%%%%%%%%%%%%%%%%%%%%%%%%%%%%%%%%%%%%%%%%%%%%%%%%%%%%%%%%%%%%%%%%%%%
%   FINJAC       numerical approximation to Jacobi matrix
%   %%%%%%
function J = finjac(FUN,r,x,epsx)
%~~~~~~~~~~~~~~~~~~~~~~~~~~~~~~~~
lx=length(x);
J=zeros(length(r),lx);
for k=1:lx
    dx=.25*epsx(k);
    xd=x;
    xd(k)=xd(k)+dx;
    rd=feval(FUN,xd);
%   ~~~~~~~~~~~~~~~~
    J(:,k)=((rd-r)/dx);
end
%%%%%%%%%%%%%%%%%%%%%%%%%%%%%%%%%%%%%%%%%%%%%%%%%%%%%%%%%%%%%%%%%%%%%%%

function printit(ipr,cnt,res,SS,x,dx,l,lc)
%        ~~~~~~~~~~~~~~~~~~~~~~~~~~~~~~~~~  Printing of intermediate results
%  ipr <  0  do not print lambda columns
%      =  0  do not print at all
%      >  0  print every (ipr)th iteration
%  cnt = -1  print out the header
%         0  print out second row of results
%        >0  print out first row of results
if ipr~=0
   if cnt<0                 %   table header
      disp('')
      disp(char('*'*ones(1,75)))
      fprintf('  itr  nfJ   SUM(r^2)        x           dx');
      if ipr>0
          fprintf('           l           lc');
```

```
      end
      fprintf('\n');
      disp(char('*'*ones(1,75)))
      disp('')
   else                          %   iteration output
      if rem(cnt,ipr)==0
          f='%12.4e ';
          if ipr>0
             fprintf(['%4.0f %4.0f ' f f f f f '\n'],...
                 cnt,res,SS, x(1),dx(1),l,lc);
          else
             fprintf(['%4.0f %4.0f ' f f f '\n'],...
                 cnt,res,SS, x(1),dx(1));
          end
          for k=2:length(x)
             fprintf([blanks(23) f f '\n'],x(k),dx(k));
          end
      end
   end
end
```
