# Supplementary material for: A model reduction method for biochemical reaction networks
Source: BMC Syst Biol. 2014 May 3;8:52. doi: 10.1186/1752-0509-8-52 (PMC4041147; doi:10.1186/1752-0509-8-52)
Supplement: Additional file 7 — A Matlab function for computing the final steady state with given initial conditions. [file 1752-0509-8-52-S7.pdf]

A program to compute the error integral between the original and a given reduced model of the yeast glycolysis.

```
function [output] = error_integral(varargin) %Simpson's rule is used to
compute integral
y1 = varargin{1};
y2 = varargin{2};
I5 = abs(y2(11,1)/y1(11,1)-1)+ abs(y2(11,5)/y1(11,5)-1)+
abs(y2(11,6)/y1(11,6)-1)+ abs(y2(11,10)/y1(11,10)-1)+
abs(y2(11,11)/y1(11,11)-1)+ abs(y2(11,12)/y1(11,12)-1)+
abs(y2(1010,1)/y1(1010,1)-1)+ abs(y2(1010,5)/y1(1010,5)-1)+
abs(y2(1010,6)/y1(1010,6)-1)+ abs(y2(1010,10)/y1(1010,10)-1)+
abs(y2(1010,11)/y1(1010,11)-1)+ abs(y2(1010,12)/y1(1010,12)-1);% note: index
of complexes corresponding to significant species [1,5,6,10,11,12]
for i = 1:499
    j = 2*i+10;
    k = 2*i+11;
    I5 = I5 + 4*(abs(y2(j,1)/y1(j,1)-1)+ abs(y2(j,5)/y1(j,5)-1)+
abs(y2(j,6)/y1(j,6)-1)+ abs(y2(j,10)/y1(j,10)-1)+ abs(y2(j,11)/y1(j,11)-1)+
abs(y2(j,12)/y1(j,12)-1)) + 2*(abs(y2(k,1)/y1(k,1)-1)+ abs(y2(k,5)/y1(k,5)-
1)+ abs(y2(k,6)/y1(k,6)-1)+ abs(y2(k,10)/y1(k,10)-1)+ abs(y2(k,11)/y1(k,11)-
1)+ abs(y2(k,12)/y1(k,12)-1));
end
I5 = 0.001*I5/18;
output = I5;
return
```
