# Supplementary material for: A model reduction method for biochemical reaction networks
Source: BMC Syst Biol. 2014 May 3;8:52. doi: 10.1186/1752-0509-8-52 (PMC4041147; doi:10.1186/1752-0509-8-52)
Supplement: Additional file 8 — A program to compute the error integral between the original and a given reduced model of the yeast glycolysis. [file 1752-0509-8-52-S8.pdf]

Original model of rat liver beta oxidation

```
function [output] = liver_Karen_fulldata(varargin)
time = varargin{1};
statevector = varargin{2};

%%%%%%%%%%%%%%%%%%%%%%%%%%%%%%%%%%%%%%%%%%%%%%%
% STATES
%%%%%%%%%%%%%%%%%%%%%%%%%%%%%%%%%%%%%%%%%%%%%%%
C16AcylCarCYT = statevector(1);
C16AcylCarMAT = statevector(2);
C16AcylCoAMAT = statevector(3);
C16EnoylCoAMAT = statevector(4);
C16HydroxyacylCoAMAT = statevector(5);
C16KetoacylCoAMAT = statevector(6);
C14AcylCarCYT = statevector(7);
C14AcylCarMAT = statevector(8);
C14AcylCoAMAT = statevector(9);
C14EnoylCoAMAT = statevector(10);
C14HydroxyacylCoAMAT = statevector(11);
C14KetoacylCoAMAT = statevector(12);
C12AcylCarCYT = statevector(13);
C12AcylCarMAT = statevector(14);
C12AcylCoAMAT = statevector(15);
C12EnoylCoAMAT = statevector(16);
C12HydroxyacylCoAMAT = statevector(17);
C12KetoacylCoAMAT = statevector(18);
C10AcylCarCYT = statevector(19);
C10AcylCarMAT = statevector(20);
C10AcylCoAMAT = statevector(21);
C10EnoylCoAMAT = statevector(22);
C10HydroxyacylCoAMAT = statevector(23);
C10KetoacylCoAMAT = statevector(24);
C8AcylCarCYT = statevector(25);
C8AcylCarMAT = statevector(26);
C8AcylCoAMAT = statevector(27);
C8EnoylCoAMAT = statevector(28);
C8HydroxyacylCoAMAT = statevector(29);
C8KetoacylCoAMAT = statevector(30);
C6AcylCarCYT = statevector(31);
C6AcylCarMAT = statevector(32);
C6AcylCoAMAT = statevector(33);
C6EnoylCoAMAT = statevector(34);
C6HydroxyacylCoAMAT = statevector(35);
C6KetoacylCoAMAT = statevector(36);
C4AcylCarCYT = statevector(37);
C4AcylCarMAT = statevector(38);
C4AcylCoAMAT = statevector(39);
C4EnoylCoAMAT = statevector(40);
C4HydroxyacylCoAMAT = statevector(41);
C4AcetoacylCoAMAT = statevector(42);
AcetylCoAMAT = statevector(43);
FADHMAT = statevector(44);
NADHMAT = statevector(45);
```

```
%%%%%%%%%%%%%%%%%%%%%%%%%%%%%%%%%%%%%%%%%%%%%%%
% PARAMETERS
%%%%%%%%%%%%%%%%%%%%%%%%%%%%%%%%%%%%%%%%%%%%%%%
C16AcylCoACYT = 26.8*exp(-0.18*time);
sfcpt1C16 = 1;
Vcpt1 = 0.012;
Kmcpt1C16AcylCoACYT = 13.8;
Kmcpt1CarCYT = 125;
Kmcpt1C16AcylCarCYT = 136;
Kmcpt1CoACYT = 40.7;
Kicpt1MalCoACYT = 9.1;
Keqcpt1 = 0.45;
ncpt1 = 2.4799;
Vfcact = 0.42;
Vrcact = 0.42;
KmcactC16AcylCarCYT = 15;
KmcactC14AcylCarCYT = 15;
KmcactC12AcylCarCYT = 15;
KmcactC10AcylCarCYT = 15;
KmcactC8AcylCarCYT = 15;
KmcactC6AcylCarCYT = 15;
KmcactC4AcylCarCYT = 15;
KmcactCarMAT =130;
KmcactC16AcylCarMAT = 15;
KmcactC14AcylCarMAT = 15;
KmcactC12AcylCarMAT = 15;
KmcactC10AcylCarMAT = 15;
KmcactC8AcylCarMAT = 15;
KmcactC6AcylCarMAT = 15;
KmcactC4AcylCarMAT = 15;
KmcactCarCYT = 130;
KicactC16AcylCarCYT = 56;
KicactC14AcylCarCYT = 56;
KicactC12AcylCarCYT = 56;
KicactC10AcylCarCYT = 56;
KicactC8AcylCarCYT = 56;
KicactC6AcylCarCYT = 56;
KicactC4AcylCarCYT = 56;
KicactCarCYT = 200;
Keqcact = 1;
sfcpt2C16 = 0.85;
sfcpt2C14 = 1;
sfcpt2C12 = 0.95;
sfcpt2C10 = 0.95;
sfcpt2C8 = 0.35;
sfcpt2C6 = 0.15;
sfcpt2C4 = 0.01;
Vcpt2 = 0.391;
Kmcpt2C16AcylCarMAT = 51;
Kmcpt2C14AcylCarMAT = 51;
Kmcpt2C12AcylCarMAT = 51;
Kmcpt2C10AcylCarMAT = 51;
Kmcpt2C8AcylCarMAT = 51;
Kmcpt2C6AcylCarMAT = 51;
Kmcpt2C4AcylCarMAT = 51;
Kmcpt2CoAMAT = 30;
```

```
Kmcpt2C16AcylCoAMAT = 38;
Kmcpt2C14AcylCoAMAT = 38;
Kmcpt2C12AcylCoAMAT = 38;
Kmcpt2C10AcylCoAMAT = 38;
Kmcpt2C8AcylCoAMAT = 38;
Kmcpt2C6AcylCoAMAT = 1000;
Kmcpt2C4AcylCoAMAT = 1000000;
Kmcpt2CarMAT = 350;
Keqcpt2 = 2.22;
sfvlcadC16 = 1;
sfvlcadC14 = 0.42;
sfvlcadC12 = 0.11;
Vvlcad = 0.008;
KmvlcadC16AcylCoAMAT = 6.5;
KmvlcadC14AcylCoAMAT = 4;
KmvlcadC12AcylCoAMAT = 2.7;
KmvlcadFAD = 0.12;
KmvlcadC16EnoylCoAMAT = 1.08;
KmvlcadC14EnoylCoAMAT = 1.08;
KmvlcadC12EnoylCoAMAT = 1.08;
KmvlcadFADH = 24.2;
Keqvlcad = 6;
sflcadC16 = 0.9;
sflcadC14 = 1;
sflcadC12 = 0.9;
sflcadC10 = 0.75;
sflcadC8 = 0.4;
Vlcad = 0.01;
KmlcadC16AcylCoAMAT = 2.5;
KmlcadC14AcylCoAMAT = 7.4;
KmlcadC12AcylCoAMAT = 9;
KmlcadC10AcylCoAMAT = 24.3;
KmlcadC8AcylCoAMAT = 123;
KmlcadFAD =0.12;
KmlcadC16EnoylCoAMAT = 1.08;
KmlcadC14EnoylCoAMAT = 1.08;
KmlcadC12EnoylCoAMAT = 1.08;
KmlcadC10EnoylCoAMAT = 1.08;
KmlcadC8EnoylCoAMAT = 1.08;
KmlcadFADH = 24.2;
Keqlcad = 6;
sfmcadC12 = 0.38;
sfmcadC10 = 0.8;
sfmcadC8 = 0.87;
sfmcadC6 = 1;
sfmcadC4 = 0.12;
Vmcad = 0.081;
KmmcadC12AcylCoAMAT = 5.7;
KmmcadC10AcylCoAMAT = 5.4;
KmmcadC8AcylCoAMAT = 4;
KmmcadC6AcylCoAMAT = 9.4;
KmmcadC4AcylCoAMAT = 135;
KmmcadFAD = 0.12;
KmmcadC12EnoylCoAMAT = 1.08;
KmmcadC10EnoylCoAMAT = 1.08;
KmmcadC8EnoylCoAMAT = 1.08;
KmmcadC6EnoylCoAMAT = 1.08;
```

```
KmmcadC4EnoylCoAMAT = 1.08;
KmmcadFADH = 24.2;
Keqmcad = 6;
sfscadC6 = 0.3;
sfscadC4 = 1;
Vscad = 0.081;
KmscadC6AcylCoAMAT = 285;
KmscadC4AcylCoAMAT = 10.7;
KmscadFAD = 0.12;
KmscadC6EnoylCoAMAT = 1.08;
KmscadC4EnoylCoAMAT = 1.08;
KmscadFADH = 24.2;
Keqscad = 6;
sfcrotC16 = 0.13;
sfcrotC14 = 0.2;
sfcrotC12 = 0.25;
sfcrotC10 = 0.33;
sfcrotC8 = 0.58;
sfcrotC6 = 0.83;
sfcrotC4 = 1;
Vcrot = 3.6;
KmcrotC16EnoylCoAMAT = 150;
KmcrotC14EnoylCoAMAT = 100;
KmcrotC12EnoylCoAMAT = 25;
KmcrotC10EnoylCoAMAT = 25;
KmcrotC8EnoylCoAMAT = 25;
KmcrotC6EnoylCoAMAT = 25;
KmcrotC4EnoylCoAMAT = 40;
KmcrotC16HydroxyacylCoAMAT = 45;
KmcrotC14HydroxyacylCoAMAT = 45;
KmcrotC12HydroxyacylCoAMAT = 45;
KmcrotC10HydroxyacylCoAMAT = 45;
KmcrotC8HydroxyacylCoAMAT = 45;
KmcrotC6HydroxyacylCoAMAT = 45;
KmcrotC4HydroxyacylCoAMAT = 45;
KicrotC4AcetoacylCoA = 1.6;
Keqcrot = 3.13;
sfmschadC16 = 0.6;
sfmschadC14 = 0.5;
sfmschadC12 = 0.43;
sfmschadC10 = 0.64;
sfmschadC8 = 0.89;
sfmschadC6 = 1;
sfmschadC4 = 0.67;
Vmschad = 1;
KmmschadC16HydroxyacylCoAMAT = 1.5;
KmmschadC14HydroxyacylCoAMAT = 1.8;
KmmschadC12HydroxyacylCoAMAT = 3.7;
KmmschadC10HydroxyacylCoAMAT = 8.8;
KmmschadC8HydroxyacylCoAMAT = 16.3;
KmmschadC6HydroxyacylCoAMAT = 28.6;
KmmschadC4HydroxyacylCoAMAT = 69.9;
KmmschadNADMAT = 58.5;
KmmschadC16KetoacylCoAMAT = 1.4;
KmmschadC14KetoacylCoAMAT = 1.4;
KmmschadC12KetoacylCoAMAT = 1.6;
KmmschadC10KetoacylCoAMAT = 2.3;
```

```
KmmschadC8KetoacylCoAMAT = 4.1;
KmmschadC6KetoacylCoAMAT = 5.8;
KmmschadC4AcetoacylCoAMAT = 16.9;
KmmschadNADHMAT = 5.4;
Keqmschad = 2.17/10^4;
sfmckatC16 = 0.197;
sfmckatC14 = 0.2;
sfmckatC12 = 0.38;
sfmckatC10 = 0.65;
sfmckatC8 = 0.81;
sfmckatC6 = 1;
sfmckatC4 = 0.49;
Vmckat = 0.377;
KmmckatC16KetoacylCoAMAT = 1.1;
KmmckatC14KetoacylCoAMAT = 1.2;
KmmckatC12KetoacylCoAMAT = 1.3;
KmmckatC10KetoacylCoAMAT = 2.1;
KmmckatC8KetoacylCoAMAT = 3.2;
KmmckatC6KetoacylCoAMAT = 6.7;
KmmckatC4AcetoacylCoAMAT = 12.4;
KmmckatCoAMAT = 26.6;
KmmckatC14AcylCoAMAT = 13.83;
KmmckatC16AcylCoAMAT = 13.83;
KmmckatC12AcylCoAMAT = 13.83;
KmmckatC10AcylCoAMAT = 13.83;
KmmckatC8AcylCoAMAT = 13.83;
KmmckatC6AcylCoAMAT = 13.83;
KmmckatC4AcylCoAMAT = 13.83;
KmmckatAcetylCoAMAT = 30;
Keqmckat = 1051;
sfmtpC16 = 1;
sfmtpC14 = 0.9;
sfmtpC12 = 0.81;
sfmtpC10 = 0.73;
sfmtpC8 = 0.34;
Vmtp = 2.84;
KmmtpC16EnoylCoAMAT = 25;
KmmtpC14EnoylCoAMAT = 25;
KmmtpC12EnoylCoAMAT = 25;
KmmtpC10EnoylCoAMAT = 25;
KmmtpC8EnoylCoAMAT = 25;
KmmtpNADMAT = 60;
KmmtpCoAMAT = 30;
KmmtpC14AcylCoAMAT = 13.83;
KmmtpC16AcylCoAMAT = 13.83;
KmmtpC12AcylCoAMAT = 13.83;
KmmtpC10AcylCoAMAT = 13.83;
KmmtpC8AcylCoAMAT = 13.83;
KmmtpC6AcylCoAMAT = 13.83;
KmmtpNADHMAT = 50;
KmmtpAcetylCoAMAT = 30;
Keqmtp = 7138.4971/10^4;
Ksacesink = 6000000;
K1acesink = 30;
Ksfadhsink = 6000000;
K1fadhsink = 0.46;
Ksnadhsink = 6000000;
```

```
K1nadhsink = 16;
%C16AcylCoACYT = 25;
CarCYT = 400;
CoACYT = 140;
MalCoACYT = 0;
CarMAT = 950;
FADtMAT = 0.77;
NADtMAT = 250;
CoAMATt = 5000;
VCYT = 0.01;
VMAT = 1.8/10^6;

%%%%%%%%%%%%%%%%%%%%%%%%%%%%%%%%%%%%%%%%%%%%%%%
% REACTION KINETICS
%%%%%%%%%%%%%%%%%%%%%%%%%%%%%%%%%%%%%%%%%%%%%%%
CoAMAT = CoAMATt - C16AcylCoAMAT - C16EnoylCoAMAT - C16HydroxyacylCoAMAT -
C16KetoacylCoAMAT - C14AcylCoAMAT - C14EnoylCoAMAT - C14HydroxyacylCoAMAT -
C14KetoacylCoAMAT - C12AcylCoAMAT - C12EnoylCoAMAT - C12HydroxyacylCoAMAT -
C12KetoacylCoAMAT - C10AcylCoAMAT - C10EnoylCoAMAT - C10HydroxyacylCoAMAT -
C10KetoacylCoAMAT - C8AcylCoAMAT - C8EnoylCoAMAT - C8HydroxyacylCoAMAT -
C8KetoacylCoAMAT - C6AcylCoAMAT - C6EnoylCoAMAT - C6HydroxyacylCoAMAT -
C6KetoacylCoAMAT - C4AcylCoAMAT - C4EnoylCoAMAT - C4HydroxyacylCoAMAT -
C4AcetoacylCoAMAT - AcetylCoAMAT;

vcpt1C16 = CPT1([sfcpt1C16, Vcpt1, Kmcpt1C16AcylCoACYT, Kmcpt1CarCYT,
Kmcpt1C16AcylCarCYT, Kmcpt1CoACYT, Kicpt1MalCoACYT, Keqcpt1, C16AcylCoACYT,
CarCYT, C16AcylCarCYT, CoACYT, MalCoACYT, ncpt1]);
vcactC16 = CACT([Vfcact, Vrcact, KmcactC16AcylCarCYT, KmcactCarMAT,
KmcactC16AcylCarMAT, KmcactCarCYT, KicactC16AcylCarCYT, KicactCarCYT,
Keqcact, C16AcylCarCYT, CarMAT, C16AcylCarMAT, CarCYT]);
vcactC14 = CACT([Vfcact, Vrcact, KmcactC14AcylCarCYT, KmcactCarMAT,
KmcactC14AcylCarMAT, KmcactCarCYT, KicactC14AcylCarCYT, KicactCarCYT,
Keqcact, C14AcylCarCYT, CarMAT, C14AcylCarMAT, CarCYT]);
vcactC12 = CACT([Vfcact, Vrcact, KmcactC12AcylCarCYT, KmcactCarMAT,
KmcactC12AcylCarMAT, KmcactCarCYT, KicactC12AcylCarCYT, KicactCarCYT,
Keqcact, C12AcylCarCYT, CarMAT, C12AcylCarMAT, CarCYT]);
vcactC10 = CACT([Vfcact, Vrcact, KmcactC10AcylCarCYT, KmcactCarMAT,
KmcactC10AcylCarMAT, KmcactCarCYT, KicactC10AcylCarCYT, KicactCarCYT,
Keqcact, C10AcylCarCYT, CarMAT, C10AcylCarMAT, CarCYT]);
vcactC8 = CACT([Vfcact, Vrcact, KmcactC8AcylCarCYT, KmcactCarMAT,
KmcactC8AcylCarMAT, KmcactCarCYT, KicactC8AcylCarCYT, KicactCarCYT, Keqcact,
C8AcylCarCYT, CarMAT, C8AcylCarMAT, CarCYT]);
vcactC6 = CACT([Vfcact, Vrcact, KmcactC6AcylCarCYT, KmcactCarMAT,
KmcactC6AcylCarMAT, KmcactCarCYT, KicactC6AcylCarCYT, KicactCarCYT, Keqcact,
C6AcylCarCYT, CarMAT, C6AcylCarMAT, CarCYT]);
vcactC4 = CACT([Vfcact, Vrcact, KmcactC4AcylCarCYT, KmcactCarMAT,
KmcactC4AcylCarMAT, KmcactCarCYT, KicactC4AcylCarCYT, KicactCarCYT, Keqcact,
C4AcylCarCYT, CarMAT, C4AcylCarMAT, CarCYT]);
vcpt2C16 = CPT2([sfcpt2C16, Vcpt2, Kmcpt2C16AcylCarMAT, Kmcpt2C14AcylCarMAT,
Kmcpt2C12AcylCarMAT, Kmcpt2C10AcylCarMAT, Kmcpt2C8AcylCarMAT,
Kmcpt2C6AcylCarMAT, Kmcpt2C4AcylCarMAT, Kmcpt2CoAMAT, Kmcpt2C16AcylCoAMAT,
Kmcpt2C14AcylCoAMAT, Kmcpt2C12AcylCoAMAT, Kmcpt2C10AcylCoAMAT,
Kmcpt2C8AcylCoAMAT, Kmcpt2C6AcylCoAMAT, Kmcpt2C4AcylCoAMAT, Kmcpt2CarMAT,
Keqcpt2, C16AcylCarMAT, C14AcylCarMAT, C12AcylCarMAT, C10AcylCarMAT,
C8AcylCarMAT, C6AcylCarMAT, C4AcylCarMAT, CoAMAT, C16AcylCoAMAT,
```

```
C14AcylCoAMAT, C12AcylCoAMAT, C10AcylCoAMAT, C8AcylCoAMAT, C6AcylCoAMAT,
C4AcylCoAMAT, CarMAT]);
vcpt2C14 = CPT2([sfcpt2C14, Vcpt2, Kmcpt2C14AcylCarMAT, Kmcpt2C16AcylCarMAT,
Kmcpt2C12AcylCarMAT, Kmcpt2C10AcylCarMAT, Kmcpt2C8AcylCarMAT,
Kmcpt2C6AcylCarMAT, Kmcpt2C4AcylCarMAT, Kmcpt2CoAMAT, Kmcpt2C14AcylCoAMAT,
Kmcpt2C16AcylCoAMAT, Kmcpt2C12AcylCoAMAT, Kmcpt2C10AcylCoAMAT,
Kmcpt2C8AcylCoAMAT, Kmcpt2C6AcylCoAMAT, Kmcpt2C4AcylCoAMAT, Kmcpt2CarMAT,
Keqcpt2, C14AcylCarMAT, C16AcylCarMAT, C12AcylCarMAT, C10AcylCarMAT,
C8AcylCarMAT, C6AcylCarMAT, C4AcylCarMAT, CoAMAT, C14AcylCoAMAT,
C16AcylCoAMAT, C12AcylCoAMAT, C10AcylCoAMAT, C8AcylCoAMAT, C6AcylCoAMAT,
C4AcylCoAMAT, CarMAT]);
vcpt2C12 = CPT2([sfcpt2C12, Vcpt2, Kmcpt2C12AcylCarMAT, Kmcpt2C16AcylCarMAT,
Kmcpt2C14AcylCarMAT, Kmcpt2C10AcylCarMAT, Kmcpt2C8AcylCarMAT,
Kmcpt2C6AcylCarMAT, Kmcpt2C4AcylCarMAT, Kmcpt2CoAMAT, Kmcpt2C12AcylCoAMAT,
Kmcpt2C16AcylCoAMAT, Kmcpt2C14AcylCoAMAT, Kmcpt2C10AcylCoAMAT,
Kmcpt2C8AcylCoAMAT, Kmcpt2C6AcylCoAMAT, Kmcpt2C4AcylCoAMAT, Kmcpt2CarMAT,
Keqcpt2, C12AcylCarMAT, C16AcylCarMAT, C14AcylCarMAT, C10AcylCarMAT,
C8AcylCarMAT, C6AcylCarMAT, C4AcylCarMAT, CoAMAT, C12AcylCoAMAT,
C16AcylCoAMAT, C14AcylCoAMAT, C10AcylCoAMAT, C8AcylCoAMAT, C6AcylCoAMAT,
C4AcylCoAMAT, CarMAT]);
vcpt2C10 = CPT2([sfcpt2C10, Vcpt2, Kmcpt2C10AcylCarMAT, Kmcpt2C16AcylCarMAT,
Kmcpt2C14AcylCarMAT, Kmcpt2C12AcylCarMAT, Kmcpt2C8AcylCarMAT,
Kmcpt2C6AcylCarMAT, Kmcpt2C4AcylCarMAT, Kmcpt2CoAMAT, Kmcpt2C10AcylCoAMAT,
Kmcpt2C16AcylCoAMAT, Kmcpt2C14AcylCoAMAT, Kmcpt2C12AcylCoAMAT,
Kmcpt2C8AcylCoAMAT, Kmcpt2C6AcylCoAMAT, Kmcpt2C4AcylCoAMAT, Kmcpt2CarMAT,
Keqcpt2, C10AcylCarMAT, C16AcylCarMAT, C14AcylCarMAT, C12AcylCarMAT,
C8AcylCarMAT, C6AcylCarMAT, C4AcylCarMAT, CoAMAT, C10AcylCoAMAT,
C16AcylCoAMAT, C14AcylCoAMAT, C12AcylCoAMAT, C8AcylCoAMAT, C6AcylCoAMAT,
C4AcylCoAMAT, CarMAT]);
vcpt2C8 = CPT2([sfcpt2C8, Vcpt2, Kmcpt2C8AcylCarMAT, Kmcpt2C16AcylCarMAT,
Kmcpt2C14AcylCarMAT, Kmcpt2C12AcylCarMAT, Kmcpt2C10AcylCarMAT,
Kmcpt2C6AcylCarMAT, Kmcpt2C4AcylCarMAT, Kmcpt2CoAMAT, Kmcpt2C8AcylCoAMAT,
Kmcpt2C16AcylCoAMAT, Kmcpt2C14AcylCoAMAT, Kmcpt2C12AcylCoAMAT,
Kmcpt2C10AcylCoAMAT, Kmcpt2C6AcylCoAMAT, Kmcpt2C4AcylCoAMAT, Kmcpt2CarMAT,
Keqcpt2, C8AcylCarMAT, C16AcylCarMAT, C14AcylCarMAT, C12AcylCarMAT,
C10AcylCarMAT, C6AcylCarMAT, C4AcylCarMAT, CoAMAT, C8AcylCoAMAT,
C16AcylCoAMAT, C14AcylCoAMAT, C12AcylCoAMAT, C10AcylCoAMAT, C6AcylCoAMAT,
C4AcylCoAMAT, CarMAT]);
vcpt2C6 = CPT2([sfcpt2C6, Vcpt2, Kmcpt2C6AcylCarMAT, Kmcpt2C16AcylCarMAT,
Kmcpt2C14AcylCarMAT, Kmcpt2C12AcylCarMAT, Kmcpt2C10AcylCarMAT,
Kmcpt2C8AcylCarMAT, Kmcpt2C4AcylCarMAT, Kmcpt2CoAMAT, Kmcpt2C6AcylCoAMAT,
Kmcpt2C16AcylCoAMAT, Kmcpt2C14AcylCoAMAT, Kmcpt2C12AcylCoAMAT,
Kmcpt2C10AcylCoAMAT, Kmcpt2C8AcylCoAMAT, Kmcpt2C4AcylCoAMAT, Kmcpt2CarMAT,
Keqcpt2, C6AcylCarMAT, C16AcylCarMAT, C14AcylCarMAT, C12AcylCarMAT,
C10AcylCarMAT, C8AcylCarMAT, C4AcylCarMAT, CoAMAT, C6AcylCoAMAT,
C16AcylCoAMAT, C14AcylCoAMAT, C12AcylCoAMAT, C10AcylCoAMAT, C8AcylCoAMAT,
C4AcylCoAMAT, CarMAT]);
vcpt2C4 = CPT2([sfcpt2C4, Vcpt2, Kmcpt2C4AcylCarMAT, Kmcpt2C16AcylCarMAT,
Kmcpt2C14AcylCarMAT, Kmcpt2C12AcylCarMAT, Kmcpt2C10AcylCarMAT,
Kmcpt2C8AcylCarMAT, Kmcpt2C6AcylCarMAT, Kmcpt2CoAMAT, Kmcpt2C4AcylCoAMAT,
Kmcpt2C16AcylCoAMAT, Kmcpt2C14AcylCoAMAT, Kmcpt2C12AcylCoAMAT,
Kmcpt2C10AcylCoAMAT, Kmcpt2C8AcylCoAMAT, Kmcpt2C6AcylCoAMAT, Kmcpt2CarMAT,
Keqcpt2, C4AcylCarMAT, C16AcylCarMAT, C14AcylCarMAT, C12AcylCarMAT,
C10AcylCarMAT, C8AcylCarMAT, C6AcylCarMAT, CoAMAT, C4AcylCoAMAT,
C16AcylCoAMAT, C14AcylCoAMAT, C12AcylCoAMAT, C10AcylCoAMAT, C8AcylCoAMAT,
C6AcylCoAMAT, CarMAT]);
```

```
vvlcadC16 = VLCAD([sfvlcadC16, Vvlcad, KmvlcadC16AcylCoAMAT,
KmvlcadC14AcylCoAMAT, KmvlcadC12AcylCoAMAT, KmvlcadFAD,
KmvlcadC16EnoylCoAMAT, KmvlcadC14EnoylCoAMAT, KmvlcadC12EnoylCoAMAT,
KmvlcadFADH, Keqvlcad, C16AcylCoAMAT, C14AcylCoAMAT, C12AcylCoAMAT, FADtMAT,
C16EnoylCoAMAT, C14EnoylCoAMAT, C12EnoylCoAMAT, FADHMAT]);
vvlcadC14 = VLCAD([sfvlcadC14, Vvlcad, KmvlcadC14AcylCoAMAT,
KmvlcadC16AcylCoAMAT, KmvlcadC12AcylCoAMAT, KmvlcadFAD,
KmvlcadC14EnoylCoAMAT, KmvlcadC16EnoylCoAMAT, KmvlcadC12EnoylCoAMAT,
KmvlcadFADH, Keqvlcad, C14AcylCoAMAT, C16AcylCoAMAT, C12AcylCoAMAT, FADtMAT,
C14EnoylCoAMAT, C16EnoylCoAMAT, C12EnoylCoAMAT, FADHMAT]);
vvlcadC12 = VLCAD([sfvlcadC12, Vvlcad, KmvlcadC12AcylCoAMAT,
KmvlcadC16AcylCoAMAT, KmvlcadC14AcylCoAMAT, KmvlcadFAD,
KmvlcadC12EnoylCoAMAT, KmvlcadC16EnoylCoAMAT, KmvlcadC14EnoylCoAMAT,
KmvlcadFADH, Keqvlcad, C12AcylCoAMAT, C16AcylCoAMAT, C14AcylCoAMAT, FADtMAT,
C12EnoylCoAMAT, C16EnoylCoAMAT, C14EnoylCoAMAT, FADHMAT]);
vlcadC16 = LCAD([sflcadC16, Vlcad, KmlcadC16AcylCoAMAT, KmlcadC14AcylCoAMAT,
KmlcadC12AcylCoAMAT, KmlcadC10AcylCoAMAT, KmlcadC8AcylCoAMAT, KmlcadFAD,
KmlcadC16EnoylCoAMAT, KmlcadC14EnoylCoAMAT, KmlcadC12EnoylCoAMAT,
KmlcadC10EnoylCoAMAT, KmlcadC8EnoylCoAMAT, KmlcadFADH, Keqlcad,
C16AcylCoAMAT, C14AcylCoAMAT, C12AcylCoAMAT, C10AcylCoAMAT, C8AcylCoAMAT,
FADtMAT, C16EnoylCoAMAT, C14EnoylCoAMAT, C12EnoylCoAMAT, C10EnoylCoAMAT,
C8EnoylCoAMAT, FADHMAT]);
vlcadC14 = LCAD([sflcadC14, Vlcad, KmlcadC14AcylCoAMAT, KmlcadC16AcylCoAMAT,
KmlcadC12AcylCoAMAT, KmlcadC10AcylCoAMAT, KmlcadC8AcylCoAMAT, KmlcadFAD,
KmlcadC14EnoylCoAMAT, KmlcadC16EnoylCoAMAT, KmlcadC12EnoylCoAMAT,
KmlcadC10EnoylCoAMAT, KmlcadC8EnoylCoAMAT, KmlcadFADH, Keqlcad,
C14AcylCoAMAT, C16AcylCoAMAT, C12AcylCoAMAT, C10AcylCoAMAT, C8AcylCoAMAT,
FADtMAT, C14EnoylCoAMAT, C16EnoylCoAMAT, C12EnoylCoAMAT, C10EnoylCoAMAT,
C8EnoylCoAMAT, FADHMAT]);
vlcadC12 = LCAD([sflcadC12, Vlcad, KmlcadC12AcylCoAMAT, KmlcadC16AcylCoAMAT,
KmlcadC14AcylCoAMAT, KmlcadC10AcylCoAMAT, KmlcadC8AcylCoAMAT, KmlcadFAD,
KmlcadC12EnoylCoAMAT, KmlcadC16EnoylCoAMAT, KmlcadC14EnoylCoAMAT,
KmlcadC10EnoylCoAMAT, KmlcadC8EnoylCoAMAT, KmlcadFADH, Keqlcad,
C12AcylCoAMAT, C16AcylCoAMAT, C14AcylCoAMAT, C10AcylCoAMAT, C8AcylCoAMAT,
FADtMAT, C14EnoylCoAMAT, C16EnoylCoAMAT, C14EnoylCoAMAT, C10EnoylCoAMAT,
C8EnoylCoAMAT, FADHMAT]);
vlcadC10 = LCAD([sflcadC10, Vlcad, KmlcadC10AcylCoAMAT, KmlcadC16AcylCoAMAT,
KmlcadC14AcylCoAMAT, KmlcadC12AcylCoAMAT, KmlcadC8AcylCoAMAT, KmlcadFAD,
KmlcadC10EnoylCoAMAT, KmlcadC16EnoylCoAMAT, KmlcadC14EnoylCoAMAT,
KmlcadC12EnoylCoAMAT, KmlcadC8EnoylCoAMAT, KmlcadFADH, Keqlcad,
C10AcylCoAMAT, C16AcylCoAMAT, C14AcylCoAMAT, C12AcylCoAMAT, C8AcylCoAMAT,
FADtMAT, C10EnoylCoAMAT, C16EnoylCoAMAT, C14EnoylCoAMAT, C12EnoylCoAMAT,
C8EnoylCoAMAT, FADHMAT]);
vlcadC8  = LCAD([sflcadC8, Vlcad, KmlcadC8AcylCoAMAT, KmlcadC16AcylCoAMAT,
KmlcadC14AcylCoAMAT, KmlcadC12AcylCoAMAT, KmlcadC10AcylCoAMAT, KmlcadFAD,
KmlcadC8EnoylCoAMAT, KmlcadC16EnoylCoAMAT,   KmlcadC14EnoylCoAMAT,
KmlcadC12EnoylCoAMAT, KmlcadC10EnoylCoAMAT, KmlcadFADH, Keqlcad,
C8AcylCoAMAT, C16AcylCoAMAT, C14AcylCoAMAT, C12AcylCoAMAT, C10AcylCoAMAT,
FADtMAT, C8EnoylCoAMAT, C16EnoylCoAMAT, C14EnoylCoAMAT, C12EnoylCoAMAT,
C10EnoylCoAMAT, FADHMAT]);
vmcadC12 = MCAD([sfmcadC12, Vmcad, KmmcadC12AcylCoAMAT, KmmcadC10AcylCoAMAT,
KmmcadC8AcylCoAMAT, KmmcadC6AcylCoAMAT, KmmcadC4AcylCoAMAT, KmmcadFAD,
KmmcadC12EnoylCoAMAT, KmmcadC10EnoylCoAMAT, KmmcadC8EnoylCoAMAT,
KmmcadC6EnoylCoAMAT, KmmcadC4EnoylCoAMAT, KmmcadFADH, Keqmcad, C12AcylCoAMAT,
C10AcylCoAMAT, C8AcylCoAMAT, C6AcylCoAMAT, C4AcylCoAMAT, FADtMAT,
C12EnoylCoAMAT, C10EnoylCoAMAT, C8EnoylCoAMAT, C6EnoylCoAMAT, C4EnoylCoAMAT,
FADHMAT]);
```

```
vmcadC10 = MCAD([sfmcadC10, Vmcad, KmmcadC10AcylCoAMAT, KmmcadC12AcylCoAMAT,
KmmcadC8AcylCoAMAT, KmmcadC6AcylCoAMAT, KmmcadC4AcylCoAMAT, KmmcadFAD,
KmmcadC10EnoylCoAMAT, KmmcadC12EnoylCoAMAT, KmmcadC8EnoylCoAMAT,
KmmcadC6EnoylCoAMAT, KmmcadC4EnoylCoAMAT, KmmcadFADH, Keqmcad, C10AcylCoAMAT,
C12AcylCoAMAT, C8AcylCoAMAT, C6AcylCoAMAT, C4AcylCoAMAT, FADtMAT,
C10EnoylCoAMAT, C12EnoylCoAMAT, C8EnoylCoAMAT, C6EnoylCoAMAT, C4EnoylCoAMAT,
FADHMAT]);
vmcadC8  = MCAD([sfmcadC8, Vmcad, KmmcadC8AcylCoAMAT, KmmcadC12AcylCoAMAT,
KmmcadC10AcylCoAMAT, KmmcadC6AcylCoAMAT, KmmcadC4AcylCoAMAT, KmmcadFAD,
KmmcadC8EnoylCoAMAT, KmmcadC12EnoylCoAMAT, KmmcadC10EnoylCoAMAT,
KmmcadC6EnoylCoAMAT, KmmcadC4EnoylCoAMAT, KmmcadFADH, Keqmcad, C8AcylCoAMAT,
C12AcylCoAMAT, C10AcylCoAMAT, C6AcylCoAMAT, C4AcylCoAMAT, FADtMAT,
C8EnoylCoAMAT, C12EnoylCoAMAT, C10EnoylCoAMAT, C6EnoylCoAMAT, C4EnoylCoAMAT,
FADHMAT]);
vmcadC6 = MCAD([sfmcadC6, Vmcad, KmmcadC6AcylCoAMAT, KmmcadC12AcylCoAMAT,
KmmcadC10AcylCoAMAT, KmmcadC8AcylCoAMAT, KmmcadC4AcylCoAMAT, KmmcadFAD,
KmmcadC6EnoylCoAMAT, KmmcadC12EnoylCoAMAT, KmmcadC10EnoylCoAMAT,
KmmcadC8EnoylCoAMAT, KmmcadC4EnoylCoAMAT, KmmcadFADH, Keqmcad, C6AcylCoAMAT,
C12AcylCoAMAT, C10AcylCoAMAT, C8AcylCoAMAT, C4AcylCoAMAT, FADtMAT,
C6EnoylCoAMAT, C12EnoylCoAMAT, C10EnoylCoAMAT, C8EnoylCoAMAT, C4EnoylCoAMAT,
FADHMAT]);
vmcadC4 = MCAD([sfmcadC4, Vmcad, KmmcadC4AcylCoAMAT, KmmcadC12AcylCoAMAT,
KmmcadC10AcylCoAMAT, KmmcadC8AcylCoAMAT, KmmcadC6AcylCoAMAT, KmmcadFAD,
KmmcadC4EnoylCoAMAT, KmmcadC12EnoylCoAMAT, KmmcadC10EnoylCoAMAT,
KmmcadC8EnoylCoAMAT, KmmcadC6EnoylCoAMAT, KmmcadFADH, Keqmcad, C4AcylCoAMAT,
C12AcylCoAMAT, C10AcylCoAMAT, C8AcylCoAMAT, C6AcylCoAMAT, FADtMAT,
C4EnoylCoAMAT, C12EnoylCoAMAT, C10EnoylCoAMAT, C8EnoylCoAMAT, C6EnoylCoAMAT,
FADHMAT]);
vscadC6 = SCAD([sfscadC6, Vscad, KmscadC6AcylCoAMAT, KmscadC4AcylCoAMAT,
KmscadFAD, KmscadC6EnoylCoAMAT, KmscadC4EnoylCoAMAT, KmscadFADH, Keqscad,
C6AcylCoAMAT, C4AcylCoAMAT, FADtMAT, C6EnoylCoAMAT, C4EnoylCoAMAT, FADHMAT]);
vscadC4 = SCAD([sfscadC4, Vscad, KmscadC4AcylCoAMAT, KmscadC6AcylCoAMAT,
KmscadFAD, KmscadC4EnoylCoAMAT, KmscadC6EnoylCoAMAT, KmscadFADH, Keqscad,
C4AcylCoAMAT, C6AcylCoAMAT, FADtMAT, C4EnoylCoAMAT, C6EnoylCoAMAT, FADHMAT]);
vcrotC16 = CROT([sfcrotC16, Vcrot, KmcrotC16EnoylCoAMAT,
KmcrotC14EnoylCoAMAT, KmcrotC12EnoylCoAMAT, KmcrotC10EnoylCoAMAT,
KmcrotC8EnoylCoAMAT, KmcrotC6EnoylCoAMAT, KmcrotC4EnoylCoAMAT,
KmcrotC16HydroxyacylCoAMAT, KmcrotC14HydroxyacylCoAMAT,
KmcrotC12HydroxyacylCoAMAT, KmcrotC10HydroxyacylCoAMAT,
KmcrotC8HydroxyacylCoAMAT, KmcrotC6HydroxyacylCoAMAT,
KmcrotC4HydroxyacylCoAMAT, KicrotC4AcetoacylCoA, Keqcrot, C16EnoylCoAMAT,
C14EnoylCoAMAT, C12EnoylCoAMAT, C10EnoylCoAMAT, C8EnoylCoAMAT, C6EnoylCoAMAT,
C4EnoylCoAMAT, C16HydroxyacylCoAMAT, C14HydroxyacylCoAMAT,
C12HydroxyacylCoAMAT, C10HydroxyacylCoAMAT, C8HydroxyacylCoAMAT,
C6HydroxyacylCoAMAT, C4HydroxyacylCoAMAT, C4AcetoacylCoAMAT]);
vcrotC14 = CROT([sfcrotC14, Vcrot, KmcrotC14EnoylCoAMAT,
KmcrotC16EnoylCoAMAT, KmcrotC12EnoylCoAMAT, KmcrotC10EnoylCoAMAT,
KmcrotC8EnoylCoAMAT, KmcrotC6EnoylCoAMAT, KmcrotC4EnoylCoAMAT,
KmcrotC14HydroxyacylCoAMAT, KmcrotC16HydroxyacylCoAMAT,
KmcrotC12HydroxyacylCoAMAT, KmcrotC10HydroxyacylCoAMAT,
KmcrotC8HydroxyacylCoAMAT, KmcrotC6HydroxyacylCoAMAT,
KmcrotC4HydroxyacylCoAMAT, KicrotC4AcetoacylCoA, Keqcrot, C14EnoylCoAMAT,
C16EnoylCoAMAT, C12EnoylCoAMAT, C10EnoylCoAMAT, C8EnoylCoAMAT, C6EnoylCoAMAT,
C4EnoylCoAMAT, C14HydroxyacylCoAMAT, C16HydroxyacylCoAMAT,
C12HydroxyacylCoAMAT, C10HydroxyacylCoAMAT, C8HydroxyacylCoAMAT,
C6HydroxyacylCoAMAT, C4HydroxyacylCoAMAT, C4AcetoacylCoAMAT]);
```

```
vcrotC12 = CROT([sfcrotC12, Vcrot, KmcrotC12EnoylCoAMAT,
KmcrotC16EnoylCoAMAT, KmcrotC14EnoylCoAMAT, KmcrotC10EnoylCoAMAT,
KmcrotC8EnoylCoAMAT, KmcrotC6EnoylCoAMAT, KmcrotC4EnoylCoAMAT,
KmcrotC12HydroxyacylCoAMAT, KmcrotC16HydroxyacylCoAMAT,
KmcrotC14HydroxyacylCoAMAT, KmcrotC10HydroxyacylCoAMAT,
KmcrotC8HydroxyacylCoAMAT, KmcrotC6HydroxyacylCoAMAT,
KmcrotC4HydroxyacylCoAMAT, KicrotC4AcetoacylCoA, Keqcrot, C12EnoylCoAMAT,
C16EnoylCoAMAT, C14EnoylCoAMAT, C10EnoylCoAMAT, C8EnoylCoAMAT, C6EnoylCoAMAT,
C4EnoylCoAMAT, C12HydroxyacylCoAMAT, C16HydroxyacylCoAMAT,
C14HydroxyacylCoAMAT, C10HydroxyacylCoAMAT, C8HydroxyacylCoAMAT,
C6HydroxyacylCoAMAT, C4HydroxyacylCoAMAT, C4AcetoacylCoAMAT]);
vcrotC10 = CROT([sfcrotC10, Vcrot, KmcrotC10EnoylCoAMAT,
KmcrotC16EnoylCoAMAT, KmcrotC14EnoylCoAMAT, KmcrotC12EnoylCoAMAT,
KmcrotC8EnoylCoAMAT, KmcrotC6EnoylCoAMAT, KmcrotC4EnoylCoAMAT,
KmcrotC10HydroxyacylCoAMAT, KmcrotC16HydroxyacylCoAMAT,
KmcrotC14HydroxyacylCoAMAT, KmcrotC12HydroxyacylCoAMAT,
KmcrotC8HydroxyacylCoAMAT, KmcrotC6HydroxyacylCoAMAT,
KmcrotC4HydroxyacylCoAMAT, KicrotC4AcetoacylCoA, Keqcrot, C10EnoylCoAMAT,
C16EnoylCoAMAT, C14EnoylCoAMAT, C12EnoylCoAMAT, C8EnoylCoAMAT, C6EnoylCoAMAT,
C4EnoylCoAMAT, C10HydroxyacylCoAMAT, C16HydroxyacylCoAMAT,
C14HydroxyacylCoAMAT, C12HydroxyacylCoAMAT, C8HydroxyacylCoAMAT,
C6HydroxyacylCoAMAT, C4HydroxyacylCoAMAT, C4AcetoacylCoAMAT]);
vcrotC8 = CROT([sfcrotC8, Vcrot, KmcrotC8EnoylCoAMAT, KmcrotC16EnoylCoAMAT,
KmcrotC14EnoylCoAMAT, KmcrotC12EnoylCoAMAT, KmcrotC10EnoylCoAMAT,
KmcrotC6EnoylCoAMAT, KmcrotC4EnoylCoAMAT, KmcrotC8HydroxyacylCoAMAT,
KmcrotC16HydroxyacylCoAMAT, KmcrotC14HydroxyacylCoAMAT,
KmcrotC12HydroxyacylCoAMAT, KmcrotC10HydroxyacylCoAMAT,
KmcrotC6HydroxyacylCoAMAT, KmcrotC4HydroxyacylCoAMAT, KicrotC4AcetoacylCoA,
Keqcrot, C8EnoylCoAMAT, C16EnoylCoAMAT, C14EnoylCoAMAT, C12EnoylCoAMAT,
C10EnoylCoAMAT, C6EnoylCoAMAT, C4EnoylCoAMAT, C8HydroxyacylCoAMAT,
C16HydroxyacylCoAMAT, C14HydroxyacylCoAMAT, C12HydroxyacylCoAMAT,
C10HydroxyacylCoAMAT, C6HydroxyacylCoAMAT, C4HydroxyacylCoAMAT,
C4AcetoacylCoAMAT]);
vcrotC6 = CROT([sfcrotC6, Vcrot, KmcrotC6EnoylCoAMAT, KmcrotC16EnoylCoAMAT,
KmcrotC14EnoylCoAMAT, KmcrotC12EnoylCoAMAT, KmcrotC10EnoylCoAMAT,
KmcrotC8EnoylCoAMAT, KmcrotC4EnoylCoAMAT, KmcrotC6HydroxyacylCoAMAT,
KmcrotC16HydroxyacylCoAMAT, KmcrotC14HydroxyacylCoAMAT,
KmcrotC12HydroxyacylCoAMAT, KmcrotC10HydroxyacylCoAMAT,
KmcrotC8HydroxyacylCoAMAT, KmcrotC4HydroxyacylCoAMAT, KicrotC4AcetoacylCoA,
Keqcrot, C6EnoylCoAMAT, C16EnoylCoAMAT, C14EnoylCoAMAT, C12EnoylCoAMAT,
C10EnoylCoAMAT, C8EnoylCoAMAT, C4EnoylCoAMAT, C6HydroxyacylCoAMAT,
C16HydroxyacylCoAMAT, C14HydroxyacylCoAMAT, C12HydroxyacylCoAMAT,
C10HydroxyacylCoAMAT, C8HydroxyacylCoAMAT, C4HydroxyacylCoAMAT,
C4AcetoacylCoAMAT]);
vcrotC4 = CROT([sfcrotC4, Vcrot, KmcrotC4EnoylCoAMAT, KmcrotC16EnoylCoAMAT,
KmcrotC14EnoylCoAMAT, KmcrotC12EnoylCoAMAT, KmcrotC10EnoylCoAMAT,
KmcrotC8EnoylCoAMAT, KmcrotC6EnoylCoAMAT, KmcrotC4HydroxyacylCoAMAT,
KmcrotC16HydroxyacylCoAMAT, KmcrotC14HydroxyacylCoAMAT,
KmcrotC12HydroxyacylCoAMAT, KmcrotC10HydroxyacylCoAMAT,
KmcrotC8HydroxyacylCoAMAT, KmcrotC6HydroxyacylCoAMAT, KicrotC4AcetoacylCoA,
Keqcrot, C4EnoylCoAMAT, C16EnoylCoAMAT, C14EnoylCoAMAT, C12EnoylCoAMAT,
C10EnoylCoAMAT, C8EnoylCoAMAT, C6EnoylCoAMAT, C4HydroxyacylCoAMAT,
C16HydroxyacylCoAMAT, C14HydroxyacylCoAMAT, C12HydroxyacylCoAMAT,
C10HydroxyacylCoAMAT, C8HydroxyacylCoAMAT, C6HydroxyacylCoAMAT,
C4AcetoacylCoAMAT]);
vmschadC16 = MSCHAD([sfmschadC16, Vmschad, KmmschadC16HydroxyacylCoAMAT,
KmmschadC14HydroxyacylCoAMAT, KmmschadC12HydroxyacylCoAMAT,
```

```
KmmschadC10HydroxyacylCoAMAT, KmmschadC8HydroxyacylCoAMAT,
KmmschadC6HydroxyacylCoAMAT, KmmschadC4HydroxyacylCoAMAT, KmmschadNADMAT,
KmmschadC16KetoacylCoAMAT, KmmschadC14KetoacylCoAMAT,
KmmschadC12KetoacylCoAMAT, KmmschadC10KetoacylCoAMAT,
KmmschadC8KetoacylCoAMAT, KmmschadC6KetoacylCoAMAT,
KmmschadC4AcetoacylCoAMAT, KmmschadNADHMAT, Keqmschad, C16HydroxyacylCoAMAT,
C14HydroxyacylCoAMAT, C12HydroxyacylCoAMAT, C10HydroxyacylCoAMAT,
C8HydroxyacylCoAMAT, C6HydroxyacylCoAMAT, C4HydroxyacylCoAMAT, NADtMAT,
C16KetoacylCoAMAT, C14KetoacylCoAMAT, C12KetoacylCoAMAT, C10KetoacylCoAMAT,
C8KetoacylCoAMAT, C6KetoacylCoAMAT, C4AcetoacylCoAMAT, NADHMAT]);
vmschadC14 = MSCHAD([sfmschadC14, Vmschad, KmmschadC14HydroxyacylCoAMAT,
KmmschadC16HydroxyacylCoAMAT, KmmschadC12HydroxyacylCoAMAT,
KmmschadC10HydroxyacylCoAMAT, KmmschadC8HydroxyacylCoAMAT,
KmmschadC6HydroxyacylCoAMAT, KmmschadC4HydroxyacylCoAMAT, KmmschadNADMAT,
KmmschadC14KetoacylCoAMAT, KmmschadC16KetoacylCoAMAT,
KmmschadC12KetoacylCoAMAT, KmmschadC10KetoacylCoAMAT,
KmmschadC8KetoacylCoAMAT, KmmschadC6KetoacylCoAMAT,
KmmschadC4AcetoacylCoAMAT, KmmschadNADHMAT, Keqmschad, C14HydroxyacylCoAMAT,
C16HydroxyacylCoAMAT, C12HydroxyacylCoAMAT, C10HydroxyacylCoAMAT,
C8HydroxyacylCoAMAT, C6HydroxyacylCoAMAT, C4HydroxyacylCoAMAT, NADtMAT,
C14KetoacylCoAMAT, C16KetoacylCoAMAT, C12KetoacylCoAMAT, C10KetoacylCoAMAT,
C8KetoacylCoAMAT, C6KetoacylCoAMAT, C4AcetoacylCoAMAT, NADHMAT]);
vmschadC12 = MSCHAD([sfmschadC12, Vmschad, KmmschadC12HydroxyacylCoAMAT,
KmmschadC16HydroxyacylCoAMAT, KmmschadC14HydroxyacylCoAMAT,
KmmschadC10HydroxyacylCoAMAT, KmmschadC8HydroxyacylCoAMAT,
KmmschadC6HydroxyacylCoAMAT, KmmschadC4HydroxyacylCoAMAT, KmmschadNADMAT,
KmmschadC12KetoacylCoAMAT, KmmschadC16KetoacylCoAMAT,
KmmschadC14KetoacylCoAMAT, KmmschadC10KetoacylCoAMAT,
KmmschadC8KetoacylCoAMAT, KmmschadC6KetoacylCoAMAT,
KmmschadC4AcetoacylCoAMAT, KmmschadNADHMAT, Keqmschad, C12HydroxyacylCoAMAT,
C16HydroxyacylCoAMAT, C14HydroxyacylCoAMAT, C10HydroxyacylCoAMAT,
C8HydroxyacylCoAMAT, C6HydroxyacylCoAMAT, C4HydroxyacylCoAMAT, NADtMAT,
C12KetoacylCoAMAT, C16KetoacylCoAMAT, C14KetoacylCoAMAT, C10KetoacylCoAMAT,
C8KetoacylCoAMAT, C6KetoacylCoAMAT, C4AcetoacylCoAMAT, NADHMAT]);
vmschadC10 = MSCHAD([sfmschadC10, Vmschad, KmmschadC10HydroxyacylCoAMAT,
KmmschadC16HydroxyacylCoAMAT, KmmschadC14HydroxyacylCoAMAT,
KmmschadC12HydroxyacylCoAMAT, KmmschadC8HydroxyacylCoAMAT,
KmmschadC6HydroxyacylCoAMAT, KmmschadC4HydroxyacylCoAMAT, KmmschadNADMAT,
KmmschadC10KetoacylCoAMAT, KmmschadC16KetoacylCoAMAT,
KmmschadC14KetoacylCoAMAT, KmmschadC12KetoacylCoAMAT,
KmmschadC8KetoacylCoAMAT, KmmschadC6KetoacylCoAMAT,
KmmschadC4AcetoacylCoAMAT, KmmschadNADHMAT, Keqmschad, C10HydroxyacylCoAMAT,
C16HydroxyacylCoAMAT, C14HydroxyacylCoAMAT, C12HydroxyacylCoAMAT,
C8HydroxyacylCoAMAT, C6HydroxyacylCoAMAT, C4HydroxyacylCoAMAT, NADtMAT,
C10KetoacylCoAMAT, C16KetoacylCoAMAT, C14KetoacylCoAMAT, C12KetoacylCoAMAT,
C8KetoacylCoAMAT, C6KetoacylCoAMAT, C4AcetoacylCoAMAT, NADHMAT]);
vmschadC8 =  MSCHAD([sfmschadC8, Vmschad, KmmschadC8HydroxyacylCoAMAT,
KmmschadC16HydroxyacylCoAMAT, KmmschadC14HydroxyacylCoAMAT,
KmmschadC12HydroxyacylCoAMAT, KmmschadC10HydroxyacylCoAMAT,
KmmschadC6HydroxyacylCoAMAT, KmmschadC4HydroxyacylCoAMAT, KmmschadNADMAT,
KmmschadC8KetoacylCoAMAT, KmmschadC16KetoacylCoAMAT,
KmmschadC14KetoacylCoAMAT, KmmschadC12KetoacylCoAMAT,
KmmschadC10KetoacylCoAMAT, KmmschadC6KetoacylCoAMAT,
KmmschadC4AcetoacylCoAMAT, KmmschadNADHMAT, Keqmschad, C8HydroxyacylCoAMAT,
C16HydroxyacylCoAMAT, C14HydroxyacylCoAMAT, C12HydroxyacylCoAMAT,
C10HydroxyacylCoAMAT, C6HydroxyacylCoAMAT, C4HydroxyacylCoAMAT, NADtMAT,
```

```
C8KetoacylCoAMAT, C16KetoacylCoAMAT, C14KetoacylCoAMAT, C12KetoacylCoAMAT,
C10KetoacylCoAMAT, C6KetoacylCoAMAT, C4AcetoacylCoAMAT, NADHMAT]);
vmschadC6 =  MSCHAD([sfmschadC6, Vmschad, KmmschadC6HydroxyacylCoAMAT,
KmmschadC16HydroxyacylCoAMAT, KmmschadC14HydroxyacylCoAMAT,
KmmschadC12HydroxyacylCoAMAT, KmmschadC10HydroxyacylCoAMAT,
KmmschadC8HydroxyacylCoAMAT, KmmschadC4HydroxyacylCoAMAT, KmmschadNADMAT,
KmmschadC6KetoacylCoAMAT, KmmschadC16KetoacylCoAMAT,
KmmschadC14KetoacylCoAMAT, KmmschadC12KetoacylCoAMAT,
KmmschadC10KetoacylCoAMAT, KmmschadC8KetoacylCoAMAT,
KmmschadC4AcetoacylCoAMAT, KmmschadNADHMAT, Keqmschad, C6HydroxyacylCoAMAT,
C16HydroxyacylCoAMAT, C14HydroxyacylCoAMAT, C12HydroxyacylCoAMAT,
C10HydroxyacylCoAMAT, C8HydroxyacylCoAMAT, C4HydroxyacylCoAMAT, NADtMAT,
C6KetoacylCoAMAT, C16KetoacylCoAMAT, C14KetoacylCoAMAT, C12KetoacylCoAMAT,
C10KetoacylCoAMAT, C8KetoacylCoAMAT, C4AcetoacylCoAMAT, NADHMAT]);
vmschadC4 =  MSCHAD([sfmschadC4, Vmschad, KmmschadC4HydroxyacylCoAMAT,
KmmschadC16HydroxyacylCoAMAT, KmmschadC14HydroxyacylCoAMAT,
KmmschadC12HydroxyacylCoAMAT, KmmschadC10HydroxyacylCoAMAT,
KmmschadC8HydroxyacylCoAMAT, KmmschadC6HydroxyacylCoAMAT, KmmschadNADMAT,
KmmschadC4AcetoacylCoAMAT, KmmschadC16KetoacylCoAMAT,
KmmschadC14KetoacylCoAMAT, KmmschadC12KetoacylCoAMAT,
KmmschadC10KetoacylCoAMAT, KmmschadC8KetoacylCoAMAT,
KmmschadC6KetoacylCoAMAT, KmmschadNADHMAT, Keqmschad, C4HydroxyacylCoAMAT,
C16HydroxyacylCoAMAT, C14HydroxyacylCoAMAT, C12HydroxyacylCoAMAT,
C10HydroxyacylCoAMAT, C8HydroxyacylCoAMAT, C6HydroxyacylCoAMAT, NADtMAT,
C4AcetoacylCoAMAT, C16KetoacylCoAMAT, C14KetoacylCoAMAT, C12KetoacylCoAMAT,
C10KetoacylCoAMAT, C8KetoacylCoAMAT, C6KetoacylCoAMAT, NADHMAT]);
vmckatC16 = MCKATA([sfmckatC16, Vmckat, KmmckatC16KetoacylCoAMAT,
KmmckatC14KetoacylCoAMAT, KmmckatC12KetoacylCoAMAT, KmmckatC10KetoacylCoAMAT,
KmmckatC8KetoacylCoAMAT, KmmckatC6KetoacylCoAMAT, KmmckatC4AcetoacylCoAMAT,
KmmckatCoAMAT, KmmckatC14AcylCoAMAT, KmmckatC16AcylCoAMAT,
KmmckatC12AcylCoAMAT, KmmckatC10AcylCoAMAT, KmmckatC8AcylCoAMAT,
KmmckatC6AcylCoAMAT, KmmckatC4AcylCoAMAT, KmmckatAcetylCoAMAT, Keqmckat,
C16KetoacylCoAMAT, C14KetoacylCoAMAT, C12KetoacylCoAMAT, C10KetoacylCoAMAT,
C8KetoacylCoAMAT, C6KetoacylCoAMAT, C4AcetoacylCoAMAT, CoAMAT, C14AcylCoAMAT,
C16AcylCoAMAT, C12AcylCoAMAT, C10AcylCoAMAT, C8AcylCoAMAT, C6AcylCoAMAT,
C4AcylCoAMAT, AcetylCoAMAT]);
vmckatC14 = MCKATA([sfmckatC14, Vmckat, KmmckatC14KetoacylCoAMAT,
KmmckatC16KetoacylCoAMAT, KmmckatC12KetoacylCoAMAT, KmmckatC10KetoacylCoAMAT,
KmmckatC8KetoacylCoAMAT, KmmckatC6KetoacylCoAMAT, KmmckatC4AcetoacylCoAMAT,
KmmckatCoAMAT, KmmckatC12AcylCoAMAT, KmmckatC16AcylCoAMAT,
KmmckatC14AcylCoAMAT, KmmckatC10AcylCoAMAT, KmmckatC8AcylCoAMAT,
KmmckatC6AcylCoAMAT, KmmckatC4AcylCoAMAT, KmmckatAcetylCoAMAT, Keqmckat,
C14KetoacylCoAMAT, C16KetoacylCoAMAT, C12KetoacylCoAMAT, C10KetoacylCoAMAT,
C8KetoacylCoAMAT, C6KetoacylCoAMAT, C4AcetoacylCoAMAT, CoAMAT, C12AcylCoAMAT,
C16AcylCoAMAT, C14AcylCoAMAT, C10AcylCoAMAT, C8AcylCoAMAT, C6AcylCoAMAT,
C4AcylCoAMAT, AcetylCoAMAT]);
vmckatC12 = MCKATA([sfmckatC12, Vmckat, KmmckatC12KetoacylCoAMAT,
KmmckatC16KetoacylCoAMAT, KmmckatC14KetoacylCoAMAT, KmmckatC10KetoacylCoAMAT,
KmmckatC8KetoacylCoAMAT, KmmckatC6KetoacylCoAMAT, KmmckatC4AcetoacylCoAMAT,
KmmckatCoAMAT, KmmckatC10AcylCoAMAT, KmmckatC16AcylCoAMAT,
KmmckatC14AcylCoAMAT, KmmckatC12AcylCoAMAT, KmmckatC8AcylCoAMAT,
KmmckatC6AcylCoAMAT, KmmckatC4AcylCoAMAT, KmmckatAcetylCoAMAT, Keqmckat,
C12KetoacylCoAMAT, C16KetoacylCoAMAT, C14KetoacylCoAMAT, C10KetoacylCoAMAT,
C8KetoacylCoAMAT, C6KetoacylCoAMAT, C4AcetoacylCoAMAT, CoAMAT, C10AcylCoAMAT,
C16AcylCoAMAT, C14AcylCoAMAT, C12AcylCoAMAT, C8AcylCoAMAT, C6AcylCoAMAT,
C4AcylCoAMAT, AcetylCoAMAT]);
```

```
vmckatC10 = MCKATA([sfmckatC10, Vmckat, KmmckatC10KetoacylCoAMAT,
KmmckatC16KetoacylCoAMAT, KmmckatC14KetoacylCoAMAT, KmmckatC12KetoacylCoAMAT,
KmmckatC8KetoacylCoAMAT, KmmckatC6KetoacylCoAMAT, KmmckatC4AcetoacylCoAMAT,
KmmckatCoAMAT, KmmckatC8AcylCoAMAT, KmmckatC16AcylCoAMAT,
KmmckatC14AcylCoAMAT, KmmckatC12AcylCoAMAT, KmmckatC10AcylCoAMAT,
KmmckatC6AcylCoAMAT, KmmckatC4AcylCoAMAT, KmmckatAcetylCoAMAT, Keqmckat,
C10KetoacylCoAMAT, C16KetoacylCoAMAT, C14KetoacylCoAMAT, C12KetoacylCoAMAT,
C8KetoacylCoAMAT, C6KetoacylCoAMAT, C4AcetoacylCoAMAT, CoAMAT, C8AcylCoAMAT,
C16AcylCoAMAT, C14AcylCoAMAT, C12AcylCoAMAT, C10AcylCoAMAT, C6AcylCoAMAT,
C4AcylCoAMAT, AcetylCoAMAT]);
vmckatC8  = MCKATA([sfmckatC8, Vmckat, KmmckatC8KetoacylCoAMAT,
KmmckatC16KetoacylCoAMAT, KmmckatC14KetoacylCoAMAT, KmmckatC12KetoacylCoAMAT,
KmmckatC10KetoacylCoAMAT, KmmckatC6KetoacylCoAMAT, KmmckatC4AcetoacylCoAMAT,
KmmckatCoAMAT, KmmckatC6AcylCoAMAT, KmmckatC16AcylCoAMAT,
KmmckatC14AcylCoAMAT, KmmckatC12AcylCoAMAT, KmmckatC10AcylCoAMAT,
KmmckatC8AcylCoAMAT, KmmckatC4AcylCoAMAT, KmmckatAcetylCoAMAT, Keqmckat,
C8KetoacylCoAMAT, C16KetoacylCoAMAT, C14KetoacylCoAMAT, C12KetoacylCoAMAT,
C10KetoacylCoAMAT, C6KetoacylCoAMAT, C4AcetoacylCoAMAT, CoAMAT, C6AcylCoAMAT,
C16AcylCoAMAT, C14AcylCoAMAT, C12AcylCoAMAT, C10AcylCoAMAT, C8AcylCoAMAT,
C4AcylCoAMAT, AcetylCoAMAT]);
vmckatC6  = MCKATA([sfmckatC6, Vmckat, KmmckatC6KetoacylCoAMAT,
KmmckatC16KetoacylCoAMAT, KmmckatC14KetoacylCoAMAT, KmmckatC12KetoacylCoAMAT,
KmmckatC10KetoacylCoAMAT, KmmckatC8KetoacylCoAMAT, KmmckatC4AcetoacylCoAMAT,
KmmckatCoAMAT, KmmckatC4AcylCoAMAT, KmmckatC16AcylCoAMAT,
KmmckatC14AcylCoAMAT, KmmckatC12AcylCoAMAT, KmmckatC10AcylCoAMAT,
KmmckatC8AcylCoAMAT, KmmckatC6AcylCoAMAT, KmmckatAcetylCoAMAT, Keqmckat,
C6KetoacylCoAMAT, C16KetoacylCoAMAT, C14KetoacylCoAMAT, C12KetoacylCoAMAT,
C10KetoacylCoAMAT, C8KetoacylCoAMAT, C4AcetoacylCoAMAT, CoAMAT, C4AcylCoAMAT,
C16AcylCoAMAT, C14AcylCoAMAT, C12AcylCoAMAT, C10AcylCoAMAT, C8AcylCoAMAT,
C6AcylCoAMAT, AcetylCoAMAT]);
vmckatC4  = MCKATB([sfmckatC4, Vmckat, KmmckatC4AcetoacylCoAMAT,
KmmckatC16KetoacylCoAMAT, KmmckatC14KetoacylCoAMAT, KmmckatC12KetoacylCoAMAT,
KmmckatC10KetoacylCoAMAT, KmmckatC8KetoacylCoAMAT, KmmckatC6KetoacylCoAMAT,
KmmckatCoAMAT, KmmckatC4AcylCoAMAT, KmmckatC16AcylCoAMAT,
KmmckatC14AcylCoAMAT, KmmckatC12AcylCoAMAT, KmmckatC10AcylCoAMAT,
KmmckatC8AcylCoAMAT, KmmckatC6AcylCoAMAT, KmmckatAcetylCoAMAT, Keqmckat,
C4AcetoacylCoAMAT, C16KetoacylCoAMAT, C14KetoacylCoAMAT, C12KetoacylCoAMAT,
C10KetoacylCoAMAT, C8KetoacylCoAMAT, C6KetoacylCoAMAT, CoAMAT, C4AcylCoAMAT,
C16AcylCoAMAT, C14AcylCoAMAT, C12AcylCoAMAT, C10AcylCoAMAT, C8AcylCoAMAT,
C6AcylCoAMAT, AcetylCoAMAT]);
vmtpC16 = MTP([sfmtpC16, Vmtp, KmmtpC16EnoylCoAMAT, KmmtpC14EnoylCoAMAT,
KmmtpC12EnoylCoAMAT, KmmtpC10EnoylCoAMAT, KmmtpC8EnoylCoAMAT, KmmtpNADMAT,
KmmtpCoAMAT, KmmtpC14AcylCoAMAT, KmmtpC16AcylCoAMAT, KmmtpC12AcylCoAMAT,
KmmtpC10AcylCoAMAT, KmmtpC8AcylCoAMAT, KmmtpC6AcylCoAMAT, KmmtpNADHMAT,
KmmtpAcetylCoAMAT, Keqmtp, C16EnoylCoAMAT, C14EnoylCoAMAT, C12EnoylCoAMAT,
C10EnoylCoAMAT, C8EnoylCoAMAT, NADtMAT, CoAMAT, C14AcylCoAMAT, C16AcylCoAMAT,
C12AcylCoAMAT, C10AcylCoAMAT, C8AcylCoAMAT, C6AcylCoAMAT, NADHMAT,
AcetylCoAMAT]);
vmtpC14 = MTP([sfmtpC14, Vmtp, KmmtpC14EnoylCoAMAT, KmmtpC16EnoylCoAMAT,
KmmtpC12EnoylCoAMAT, KmmtpC10EnoylCoAMAT, KmmtpC8EnoylCoAMAT, KmmtpNADMAT,
KmmtpCoAMAT, KmmtpC12AcylCoAMAT, KmmtpC16AcylCoAMAT, KmmtpC14AcylCoAMAT,
KmmtpC10AcylCoAMAT, KmmtpC8AcylCoAMAT, KmmtpC6AcylCoAMAT, KmmtpNADHMAT,
KmmtpAcetylCoAMAT, Keqmtp, C14EnoylCoAMAT, C16EnoylCoAMAT, C12EnoylCoAMAT,
C10EnoylCoAMAT, C8EnoylCoAMAT, NADtMAT, CoAMAT, C12AcylCoAMAT, C16AcylCoAMAT,
C14AcylCoAMAT, C10AcylCoAMAT, C8AcylCoAMAT, C6AcylCoAMAT, NADHMAT,
AcetylCoAMAT]);
```

```
vmtpC12 = MTP([sfmtpC12, Vmtp, KmmtpC12EnoylCoAMAT, KmmtpC16EnoylCoAMAT,
KmmtpC14EnoylCoAMAT, KmmtpC10EnoylCoAMAT, KmmtpC8EnoylCoAMAT, KmmtpNADMAT,
KmmtpCoAMAT, KmmtpC10AcylCoAMAT, KmmtpC16AcylCoAMAT, KmmtpC14AcylCoAMAT,
KmmtpC12AcylCoAMAT, KmmtpC8AcylCoAMAT, KmmtpC6AcylCoAMAT, KmmtpNADHMAT,
KmmtpAcetylCoAMAT, Keqmtp, C12EnoylCoAMAT, C16EnoylCoAMAT, C14EnoylCoAMAT,
C10EnoylCoAMAT, C8EnoylCoAMAT, NADtMAT, CoAMAT, C10AcylCoAMAT, C16AcylCoAMAT,
C14AcylCoAMAT, C12AcylCoAMAT, C8AcylCoAMAT, C6AcylCoAMAT, NADHMAT,
AcetylCoAMAT]);
vmtpC10 = MTP([sfmtpC10, Vmtp, KmmtpC10EnoylCoAMAT, KmmtpC16EnoylCoAMAT,
KmmtpC14EnoylCoAMAT, KmmtpC12EnoylCoAMAT, KmmtpC8EnoylCoAMAT, KmmtpNADMAT,
KmmtpCoAMAT, KmmtpC8AcylCoAMAT, KmmtpC16AcylCoAMAT, KmmtpC14AcylCoAMAT,
KmmtpC12AcylCoAMAT, KmmtpC10AcylCoAMAT, KmmtpC6AcylCoAMAT, KmmtpNADHMAT,
KmmtpAcetylCoAMAT, Keqmtp, C10EnoylCoAMAT, C16EnoylCoAMAT, C14EnoylCoAMAT,
C12EnoylCoAMAT, C8EnoylCoAMAT, NADtMAT, CoAMAT, C8AcylCoAMAT, C16AcylCoAMAT,
C14AcylCoAMAT, C12AcylCoAMAT, C10AcylCoAMAT, C6AcylCoAMAT, NADHMAT,
AcetylCoAMAT]);
vmtpC8  = MTP([sfmtpC8, Vmtp, KmmtpC8EnoylCoAMAT, KmmtpC16EnoylCoAMAT,
KmmtpC14EnoylCoAMAT, KmmtpC12EnoylCoAMAT, KmmtpC10EnoylCoAMAT, KmmtpNADMAT,
KmmtpCoAMAT, KmmtpC6AcylCoAMAT, KmmtpC16AcylCoAMAT, KmmtpC14AcylCoAMAT,
KmmtpC12AcylCoAMAT, KmmtpC10AcylCoAMAT, KmmtpC8AcylCoAMAT, KmmtpNADHMAT,
KmmtpAcetylCoAMAT, Keqmtp, C8EnoylCoAMAT, C16EnoylCoAMAT, C14EnoylCoAMAT,
C12EnoylCoAMAT, C10EnoylCoAMAT, NADtMAT, CoAMAT, C6AcylCoAMAT, C16AcylCoAMAT,
C14AcylCoAMAT, C12AcylCoAMAT, C10AcylCoAMAT, C8AcylCoAMAT, NADHMAT,
AcetylCoAMAT]);
vacesink = RES([Ksacesink, AcetylCoAMAT, K1acesink]);
vfadhsink = RES([Ksfadhsink, FADHMAT, K1fadhsink]);
vnadhsink = RES([Ksnadhsink, NADHMAT, K1nadhsink]);

%%%%%%%%%%%%%%%%%%%%%%%%%%%%%%%%%%%%%%%%%%%%%%%
% DIFFERENTIAL EQUATIONS
%%%%%%%%%%%%%%%%%%%%%%%%%%%%%%%%%%%%%%%%%%%%%%%
output(1) = (vcpt1C16 - vcactC16)/VCYT;
output(2) = (vcactC16 - vcpt2C16)/VMAT;
output(3) = (vcpt2C16 - vvlcadC16 - vlcadC16)/VMAT;
output(4) = (vvlcadC16 + vlcadC16 - vcrotC16 - vmtpC16)/VMAT;
output(5) = (vcrotC16 - vmschadC16)/VMAT;
output(6) = (vmschadC16 - vmckatC16)/VMAT;
output(7) = - vcactC14/VCYT;
output(8) = (vcactC14 - vcpt2C14)/VMAT;
output(9) = (vcpt2C14 + vmtpC16 + vmckatC16 - vvlcadC14 - vlcadC14)/VMAT;
output(10) = (vvlcadC14 + vlcadC14 - vcrotC14 - vmtpC14)/VMAT;
output(11) = (vcrotC14 - vmschadC14)/VMAT;
output(12) = (vmschadC14 - vmckatC14)/VMAT;
output(13) = - vcactC12/VCYT;
output(14) = (vcactC12 - vcpt2C12)/VMAT;
output(15) = (vcpt2C12 + vmtpC14 + vmckatC14 - vvlcadC12 - vlcadC12 -
vmcadC12)/VMAT;
output(16) = (vvlcadC12 + vlcadC12 + vmcadC12 - vcrotC12 - vmtpC12)/VMAT;
output(17) = (vcrotC12 - vmschadC12)/VMAT;
output(18) = (vmschadC12 - vmckatC12)/VMAT;
output(19) = - vcactC10/VCYT;
output(20) = (vcactC10 - vcpt2C10)/VMAT;
output(21) = (vcpt2C10 + vmtpC12 + vmckatC12 - vlcadC10 - vmcadC10)/VMAT;
output(22) = (vlcadC10 + vmcadC10 - vcrotC10 - vmtpC10)/VMAT;
output(23) = (vcrotC10 - vmschadC10)/VMAT;
output(24) = (vmschadC10 - vmckatC10)/VMAT;
output(25) = - vcactC8/VCYT;
```

```
output(26) = (vcactC8 - vcpt2C8)/VMAT;
output(27) = (vcpt2C8 + vmtpC10 + vmckatC10 - vlcadC8 - vmcadC8)/VMAT;
output(28) = (vlcadC8 + vmcadC8 - vcrotC8 - vmtpC8)/VMAT;
output(29) = (vcrotC8 - vmschadC8)/VMAT;
output(30) = (vmschadC8 - vmckatC8)/VMAT;
output(31) = - vcactC6/VCYT;
output(32) = (vcactC6 - vcpt2C6)/VMAT;
output(33) = (vcpt2C6 + vmtpC8 + vmckatC8 - vmcadC6 - vscadC6)/VMAT;
output(34) = (vmcadC6 + vscadC6 - vcrotC6)/VMAT;
output(35) = (vcrotC6 - vmschadC6)/VMAT;
output(36) = (vmschadC6 - vmckatC6)/VMAT;
output(37) = - vcactC4/VCYT;
output(38) = (vcactC4 - vcpt2C4)/VMAT;
output(39) = (vcpt2C4 + vmckatC6 - vmcadC4 - vscadC4)/VMAT;
output(40) = (vmcadC4 + vscadC4 - vcrotC4)/VMAT;
output(41) = (vcrotC4 - vmschadC4)/VMAT;
output(42) = (vmschadC4 - vmckatC4)/VMAT;
output(43) = (1/VMAT)*(vmtpC16 + vmckatC16 + vmtpC14 + vmckatC14 + vmtpC12 +
vmckatC12 + vmtpC10 + vmckatC10 + vmtpC8 + vmckatC8 + vmckatC6 + 2*vmckatC4 -
vacesink);
output(44) = (1/VMAT)*(vvlcadC16 + vvlcadC14 + vvlcadC12 + vlcadC16 +
vlcadC14 + vlcadC12 + vlcadC10 + vlcadC8 + vmcadC12 + vmcadC10 + vmcadC8 +
vmcadC6 + vmcadC4 + vscadC6 + vscadC4 - vfadhsink);
output(45) = (1/VMAT)*(vmtpC16 + vmtpC14 + vmtpC12 + vmtpC10 + vmtpC8 +
vmschadC16 + vmschadC14 + vmschadC12 + vmschadC10 + vmschadC8 + vmschadC6 +
vmschadC4 - vnadhsink);
%%%%%%%%%%%%%%%%%%%%%%%%%%%%%%%%%%%%%%%%%%%%%%%
% RETURN VALUES
%%%%%%%%%%%%%%%%%%%%%%%%%%%%%%%%%%%%%%%%%%%%%%%
% return a column vector
output = output(:);
%end;

function output = CPT1(input)

sf = input(1);
V = input(2);
Kms1 = input(3);
Kms2 = input(4);
Kmp1 = input(5);
Kmp2 = input(6);
Ki1 = input(7);
Keq = input(8);
S1 = input(9);
S2 = input(10);
P1 = input(11);
P2 = input(12);
I1 = input(13);
n = input(14);

output = (sf*V*((S1*S2)/(Kms1*Kms2) - (P1*P2)/(Kms1*Kms2*Keq)))/((1 + S1/Kms1
+ P1/Kmp1 + (I1/Ki1)^n)*(1 + S2/Kms2 + P2/Kmp2));
%end;

function output = CACT(input)
```

```
Vf = input(1);
Vr = input(2);
Kms1 = input(3);
Kms2 = input(4);
Kmp1 = input(5);
Kmp2 = input(6);
Kis1 = input(7);
Kip2 = input(8);
Keq = input(9);
S1 = input(10);
S2 = input(11);
P1 = input(12);
P2 = input(13);

output = (Vf*(S1*S2 - (P1*P2)/Keq))/(S1*S2 + Kms2*S1 + Kms1*S2*(1 + P2/Kip2)
+ (Vf/(Vr*Keq))*(Kmp2*P1*(1 + S1/Kis1) + P2*(Kmp1 + P1)));
%end;

function output = CPT2(input)

sf = input(1);
V = input(2);
Kms1 = input(3);
Kms2 = input(4);
Kms3 = input(5);
Kms4 = input(6);
Kms5 = input(7);
Kms6 = input(8);
Kms7 = input(9);
Kms8 = input(10);
Kmp1 = input(11);
Kmp2 = input(12);
Kmp3 = input(13);
Kmp4 = input(14);
Kmp5 = input(15);
Kmp6 = input(16);
Kmp7 = input(17);
Kmp8 = input(18);
Keq = input(19);
S1 = input(20);
S2 = input(21);
S3 = input(22);
S4 = input(23);
S5 = input(24);
S6 = input(25);
S7 = input(26);
S8 = input(27);
P1 = input(28);
P2 = input(29);
P3 = input(30);
P4 = input(31);
P5 = input(32);
P6 = input(33);
P7 = input(34);
P8 = input(35);
```

```
output = (sf*V*((S1*S8)/(Kms1*Kms8) - (P1*P8)/(Kms1*Kms8*Keq)))/((1 + S1/Kms1
+ P1/Kmp1 + S2/Kms2 + P2/Kmp2 + S3/Kms3 + P3/Kmp3 + S4/Kms4 + P4/Kmp4 +
S5/Kms5 + P5/Kmp5 + S6/Kms6 + P6/Kmp6 + S7/Kms7 + P7/Kmp7)*(1 + S8/Kms8 +
P8/Kmp8));
%end;

function output = VLCAD(input)

sf = input(1);
V = input(2);
Kms1 = input(3);
Kms2 = input(4);
Kms3 = input(5);
Kms4 = input(6);
Kmp1 = input(7);
Kmp2 = input(8);
Kmp3 = input(9);
Kmp4 = input(10);
Keq = input(11);
S1 = input(12);
S2 = input(13);
S3 = input(14);
S4 = input(15);
P1 = input(16);
P2 = input(17);
P3 = input(18);
P4 = input(19);

output = (sf*V*((S1*(S4 - P4))/(Kms1*Kms4) - (P1*P4)/(Kms1*Kms4*Keq)))/((1 +
S1/Kms1 + P1/Kmp1 + S2/Kms2 + P2/Kmp2 + S3/Kms3 + P3/Kmp3)*(1 + (S4 -
P4)/Kms4 + P4/Kmp4));
%end;

function output = LCAD(input)

sf = input(1);
V = input(2);
Kms1 = input(3);
Kms2 = input(4);
Kms3 = input(5);
Kms4 = input(6);
Kms5 = input(7);
Kms6 = input(8);
Kmp1 = input(9);
Kmp2 = input(10);
Kmp3 = input(11);
Kmp4 = input(12);
Kmp5 = input(13);
Kmp6 = input(14);
Keq = input(15);
S1 = input(16);
S2 = input(17);
S3 = input(18);
S4 = input(19);
S5 = input(20);
S6 = input(21);
```

```
P1 = input(22);
P2 = input(23);
P3 = input(24);
P4 = input(25);
P5 = input(26);
P6 = input(27);

output = (sf*V*((S1*(S6 - P6))/(Kms1*Kms6) - (P1*P6)/(Kms1*Kms6*Keq)))/((1 +
S1/Kms1 + P1/Kmp1 + S2/Kms2 + P2/Kmp2 + S3/Kms3 + P3/Kmp3 + S4/Kms4 + P4/Kmp4
+ S5/Kms5 + P5/Kmp5)*(1 + (S6 - P6)/Kms6 + P6/Kmp6));
%end;

function output = MCAD(input)

sf = input(1);
V = input(2);
Kms1 = input(3);
Kms2 = input(4);
Kms3 = input(5);
Kms4 = input(6);
Kms5 = input(7);
Kms6 = input(8);
Kmp1 = input(9);
Kmp2 = input(10);
Kmp3 = input(11);
Kmp4 = input(12);
Kmp5 = input(13);
Kmp6 = input(14);
Keq = input(15);
S1 = input(16);
S2 = input(17);
S3 = input(18);
S4 = input(19);
S5 = input(20);
S6 = input(21);
P1 = input(22);
P2 = input(23);
P3 = input(24);
P4 = input(25);
P5 = input(26);
P6 = input(27);

output = (sf*V*((S1*(S6 - P6))/(Kms1*Kms6) - (P1*P6)/(Kms1*Kms6*Keq)))/((1 +
S1/Kms1 + P1/Kmp1 + S2/Kms2 + P2/Kmp2 + S3/Kms3 + P3/Kmp3 + S4/Kms4 + P4/Kmp4
+ S5/Kms5 + P5/Kmp5)*(1 + (S6 - P6)/Kms6 + P6/Kmp6));
%end;

function output = SCAD(input)

sf = input(1);
V = input(2);
Kms1 = input(3);
Kms2 = input(4);
Kms3 = input(5);
Kmp1 = input(6);
Kmp2 = input(7);
```

```
Kmp3 = input(8);
Keq = input(9);
S1 = input(10);
S2 = input(11);
S3 = input(12);
P1 = input(13);
P2 = input(14);
P3 = input(15);

output = (sf*V*((S1*(S3 - P3))/(Kms1*Kms3) - (P1*P3)/(Kms1*Kms3*Keq)))/((1 +
S1/Kms1 + P1/Kmp1 + S2/Kms2 + P2/Kmp2)*(1 + (S3 - P3)/Kms3 + P3/Kmp3));

%end;

function output = CROT(input)

sf = input(1);
V = input(2);
Kms1 = input(3);
Kms2 = input(4);
Kms3 = input(5);
Kms4 = input(6);
Kms5 = input(7);
Kms6 = input(8);
Kms7 = input(9);
Kmp1 = input(10);
Kmp2 = input(11);
Kmp3 = input(12);
Kmp4 = input(13);
Kmp5 = input(14);
Kmp6 = input(15);
Kmp7 = input(16);
Ki = input(17);
Keq = input(18);
S1 = input(19);
S2 = input(20);
S3 = input(21);
S4 = input(22);
S5 = input(23);
S6 = input(24);
S7 = input(25);
P1 = input(26);
P2 = input(27);
P3 = input(28);
P4 = input(29);
P5 = input(30);
P6 = input(31);
P7 = input(32);
I1 = input(33);

output = (sf*V*(S1/Kms1 - P1/(Kms1*Keq)))/(1 + S1/Kms1 + P1/Kmp1 + S2/Kms2 +
P2/Kmp2 + S3/Kms3 + P3/Kmp3 + S4/Kms4 + P4/Kmp4 + S5/Kms5 + P5/Kmp5 + S6/Kms6
+ P6/Kmp6 + S7/Kms7 + P7/Kmp7 + I1/Ki);
%end;

function output = MSCHAD(input)
```

```
sf = input(1);
V = input(2);
Kms1 = input(3);
Kms2 = input(4);
Kms3 = input(5);
Kms4 = input(6);
Kms5 = input(7);
Kms6 = input(8);
Kms7 = input(9);
Kms8 = input(10);
Kmp1 = input(11);
Kmp2 = input(12);
Kmp3 = input(13);
Kmp4 = input(14);
Kmp5 = input(15);
Kmp6 = input(16);
Kmp7 = input(17);
Kmp8 = input(18);
Keq = input(19);
S1 = input(20);
S2 = input(21);
S3 = input(22);
S4 = input(23);
S5 = input(24);
S6 = input(25);
S7 = input(26);
S8 = input(27);
P1 = input(28);
P2 = input(29);
P3 = input(30);
P4 = input(31);
P5 = input(32);
P6 = input(33);
P7 = input(34);
P8 = input(35);

output = (sf*V*((S1*(S8 - P8))/(Kms1*Kms8) - (P1*P8)/(Kms1*Kms8*Keq)))/((1 +
S1/Kms1 + P1/Kmp1 + S2/Kms2 + P2/Kmp2 + S3/Kms3 + P3/Kmp3 + S4/Kms4 + P4/Kmp4
+ S5/Kms5 + P5/Kmp5 + S6/Kms6 + P6/Kmp6 + S7/Kms7 + P7/Kmp7)*(1 + (S8 -
P8)/Kms8 + P8/Kmp8));

%end;

function output = MCKATA(input)

sf = input(1);
V = input(2);
Kms1 = input(3);
Kms2 = input(4);
Kms3 = input(5);
Kms4 = input(6);
Kms5 = input(7);
Kms6 = input(8);
Kms7 = input(9);
Kms8 = input(10);
```

```
Kmp1 = input(11);
Kmp2 = input(12);
Kmp3 = input(13);
Kmp4 = input(14);
Kmp5 = input(15);
Kmp6 = input(16);
Kmp7 = input(17);
Kmp8 = input(18);
Keq = input(19);
S1 = input(20);
S2 = input(21);
S3 = input(22);
S4 = input(23);
S5 = input(24);
S6 = input(25);
S7 = input(26);
S8 = input(27);
P1 = input(28);
P2 = input(29);
P3 = input(30);
P4 = input(31);
P5 = input(32);
P6 = input(33);
P7 = input(34);
P8 = input(35);

output = (sf*V*((S1*S8)/(Kms1*Kms8) - (P1*P8)/(Kms1*Kms8*Keq)))/((1 + S1/Kms1
+ P1/Kmp1 + S2/Kms2 + P2/Kmp2 + S3/Kms3 + P3/Kmp3 + S4/Kms4 + P4/Kmp4 +
S5/Kms5 + P5/Kmp5 + S6/Kms6 + P6/Kmp6 + S7/Kms7 + P7/Kmp7 + P8/Kmp8)*(1 +
S8/Kms8 + P8/Kmp8));
%end;

function output = MCKATB(input)

sf = input(1);
V = input(2);
Kms1 = input(3);
Kms2 = input(4);
Kms3 = input(5);
Kms4 = input(6);
Kms5 = input(7);
Kms6 = input(8);
Kms7 = input(9);
Kms8 = input(10);
Kmp1 = input(11);
Kmp2 = input(12);
Kmp3 = input(13);
Kmp4 = input(14);
Kmp5 = input(15);
Kmp6 = input(16);
Kmp7 = input(17);
Kmp8 = input(18);
Keq = input(19);
S1 = input(20);
S2 = input(21);
S3 = input(22);
```

```
S4 = input(23);
S5 = input(24);
S6 = input(25);
S7 = input(26);
S8 = input(27);
P1 = input(28);
P2 = input(29);
P3 = input(30);
P4 = input(31);
P5 = input(32);
P6 = input(33);
P7 = input(34);
P8 = input(35);

output = (sf*V*((S1*S8)/(Kms1*Kms8) - (P8*P8)/(Kms1*Kms8*Keq)))/((1 + S1/Kms1
+ P1/Kmp1 + S2/Kms2 + P2/Kmp2 + S3/Kms3 + P3/Kmp3 + S4/Kms4 + P4/Kmp4 +
S5/Kms5 + P5/Kmp5 + S6/Kms6 + P6/Kmp6 + S7/Kms7 + P7/Kmp7 + P8/Kmp8)*(1 +
S8/Kms8 + P8/Kmp8));
%end;

function output = MTP(input)
sf = input(1);
V = input(2);
Kms1 = input(3);
Kms2 = input(4);
Kms3 = input(5);
Kms4 = input(6);
Kms5 = input(7);
%Kms6 = input(8);
Kms7 = input(8);
Kms8 = input(9);
Kmp1 = input(10);
Kmp2 = input(11);
Kmp3 = input(12);
Kmp4 = input(13);
Kmp5 = input(14);
Kmp6 = input(15);
Kmp7 = input(16);
Kmp8 = input(17);
Keq = input(18);
S1 = input(19);
S2 = input(20);
S3 = input(21);
S4 = input(22);
S5 = input(23);
%S6 = input(25);
S7 = input(24);
S8 = input(25);
P1 = input(26);
P2 = input(27);
P3 = input(28);
P4 = input(29);
P5 = input(30);
P6 = input(31);
P7 = input(32);
P8 = input(33);
```

```
output = (sf*V*((S1*(S7 - P7)*S8)/(Kms1*Kms7*Kms8) -
(P1*P7*P8)/(Kms1*Kms7*Kms8*Keq)))/((1 + S1/Kms1 + P1/Kmp1 + S2/Kms2 + P2/Kmp2
+ S3/Kms3 + P3/Kmp3 + S4/Kms4 + P4/Kmp4 + S5/Kms5 + P5/Kmp5 + P6/Kmp6)*(1 +
(S7 - P7)/Kms7 + P7/Kmp7)*(1 + S8/Kms8 + P8/Kmp8));
%end;

function output = RES(input)

Ks = input(1);
S = input(2);
K1 = input(3);

output = Ks*(S - K1);
%end;
```
