# Supplementary material for: A model reduction method for biochemical reaction networks
Source: BMC Syst Biol. 2014 May 3;8:52. doi: 10.1186/1752-0509-8-52 (PMC4041147; doi:10.1186/1752-0509-8-52)
Supplement: Additional file 9 — Original model of rat liver beta oxidation. [file 1752-0509-8-52-S9.pdf]

Reduced Model of rat liver beta oxidation

```
function [output] = liver_Karen_red(varargin)
time = varargin{1};
statevector = varargin{2};

%%%%%%%%%%%%%%%%%%%%%%%%%%%%%%%%%%%%%%%%%%%%%%%
% STATES
%%%%%%%%%%%%%%%%%%%%%%%%%%%%%%%%%%%%%%%%%%%%%%%
C16AcylCarCYT = statevector(1);
C16AcylCarMAT = statevector(2);
C16AcylCoAMAT = statevector(3);
C16EnoylCoAMAT = statevector(4);
C14AcylCarCYT = statevector(5);
C14AcylCarMAT = statevector(6);
C14AcylCoAMAT = statevector(7);
C14EnoylCoAMAT = statevector(8);
C12AcylCarCYT = statevector(9);
C12AcylCarMAT = statevector(10);
C12AcylCoAMAT = statevector(11);
C12EnoylCoAMAT = statevector(12);
C10AcylCarCYT = statevector(13);
C10AcylCarMAT = statevector(14);
C10AcylCoAMAT = statevector(15);
C10EnoylCoAMAT = statevector(16);
C8AcylCarCYT = statevector(17);
C8AcylCarMAT = statevector(18);
C8AcylCoAMAT = statevector(19);
C8EnoylCoAMAT = statevector(20);
C6AcylCarCYT = statevector(21);
C6AcylCarMAT = statevector(22);
C6AcylCoAMAT = statevector(23);
C6EnoylCoAMAT = statevector(24);
C6KetoacylCoAMAT = statevector(25);
C4AcylCarCYT = statevector(26);
C4AcylCarMAT = statevector(27);
C4AcylCoAMAT = statevector(28);
C4EnoylCoAMAT = statevector(29);
C4AcetoacylCoAMAT = statevector(30);
AcetylCoAMAT = statevector(31);

%%%%%%%%%%%%%%%%%%%%%%%%%%%%%%%%%%%%%%%%%%%%%%%
% PARAMETERS (Note that we have removed all the redundant parameters)
%%%%%%%%%%%%%%%%%%%%%%%%%%%%%%%%%%%%%%%%%%%%%%%
C16AcylCoACYT = 26.8*exp(-0.18*time);
Vcpt1 = 0.012;
Kmcpt1C16AcylCoACYT = 13.8;
Kmcpt1CarCYT = 125;                              %
Kmcpt1C16AcylCarCYT = 136;
Kmcpt1CoACYT = 40.7;
Kicpt1MalCoACYT = 9.1;
Keqcpt1 = 0.45;
ncpt1 = 2.4799;
Vfcact = 0.42;
```

```
KmcactC16AcylCarCYT = 15;
KmcactCarMAT = 130;
KicactC16AcylCarCYT = 56;
KicactCarCYT = 200;
sfcpt2C16 = 0.85;
sfcpt2C14 = 1;
sfcpt2C12 = 0.95;
sfcpt2C8 = 0.35;
sfcpt2C6 = 0.15;
sfcpt2C4 = 0.01;
Vcpt2 = 0.391;
Kmcpt2C16AcylCarMAT = 51;
Kmcpt2CoAMAT = 30;
Kmcpt2C16AcylCoAMAT = 38;
Kmcpt2C6AcylCoAMAT = 1000;
Kmcpt2C4AcylCoAMAT = 1000000;
Kmcpt2CarMAT = 350;
Keqcpt2 = 2.22;
sfvlcadC16 = 1;
sfvlcadC14 = 0.42;
sfvlcadC12 = 0.11;
Vvlcad = 0.008;
KmvlcadC16AcylCoAMAT = 6.5;
KmvlcadC14AcylCoAMAT = 4;
KmvlcadC12AcylCoAMAT = 2.7;
KmvlcadFAD = 0.12;
KmcadEnoylCoAMAT = 1.08;
KmvlcadFADH = 24.2;
Keqcad = 6;
sflcadC16 = 0.9;
sflcadC14 = 1;
sflcadC12 = 0.9;
sflcadC10 = 0.75;
Vlcad = 0.01;
KmlcadC16AcylCoAMAT = 2.5;
KmlcadC14AcylCoAMAT = 7.4;
KmlcadC12AcylCoAMAT = 9;
KmlcadC10AcylCoAMAT = 24.3;
KmlcadC8AcylCoAMAT = 123;
KmlcadFAD =0.12;
KmlcadFADH = 24.2;
sfmcadC12 = 0.38;
sfmcadC10 = 0.8;
sfmcadC8 = 0.87;
sfmcadC6 = 1;
Vmcad = 0.081;
KmmcadC12AcylCoAMAT = 5.7;
KmmcadC10AcylCoAMAT = 5.4;
KmmcadC8AcylCoAMAT = 4;
KmmcadC6AcylCoAMAT = 9.4;
KmmcadC4AcylCoAMAT = 135;
KmmcadFAD = 0.12;
KmmcadFADH = 24.2;                          %
sfscadC4 = 1;
Vscad = 0.081;
KmscadC6AcylCoAMAT = 285;
KmscadC4AcylCoAMAT = 10.7;
```

```
KmscadFAD = 0.12;
KmscadFADH = 24.2;
sfcrotC6 = 0.83;
sfcrotC4 = 1;
Vcrot = 3.6;
KmcrotC6EnoylCoAMAT = 25;
KmcrotC4EnoylCoAMAT = 40;
KicrotC4AcetoacylCoA = 1.6;
Keqcrot = 3.13;
sfmschadC6 = 1;
sfmschadC4 = 0.67;
Vmschad = 1;
KmmschadC6HydroxyacylCoAMAT = 28.6;
KmmschadC4HydroxyacylCoAMAT = 69.9;
KmmschadNADMAT = 58.5;
KmmschadC6KetoacylCoAMAT = 5.8;
KmmschadC4AcetoacylCoAMAT = 16.9;
KmmschadNADHMAT = 5.4;
Keqmschad = 2.17/10^4;
sfmckatC6 = 1;
sfmckatC4 = 0.49;
Vmckat = 0.377;
KmmckatC6KetoacylCoAMAT = 6.7;
KmmckatC4AcetoacylCoAMAT = 12.4;
KmmckatCoAMAT = 26.6;
KmmckatC4AcylCoAMAT = 13.83;
KmmckatAcetylCoAMAT = 30;
Keqmckat = 1051;
sfmtpC16 = 1;
sfmtpC14 = 0.9;
sfmtpC12 = 0.81;
sfmtpC10 = 0.73;
sfmtpC8 = 0.34;
Vmtp = 2.84;
KmmtpC16EnoylCoAMAT = 25;
KmmtpNADMAT = 60;
KmmtpCoAMAT = 30;
KmmtpC16AcylCoAMAT = 13.83;
KmmtpNADHMAT = 50;
KmmtpAcetylCoAMAT = 30;
Keqmtp = 7138.4971/10^4;
CarCYT = 400;
CoACYT = 140;
MalCoACYT = 0;
CarMAT = 950;
FADtMAT = 0.77;
NADtMAT = 250;
CoAMATt = 5000;
VCYT = 0.01;
VMAT = 1.8/10^6;
Ksacesink = 6000000;
K1acesink = 30;
FADHMAT = 0.46;
NADHMAT = 16;
NAD = NADtMAT - NADHMAT;

%%%%%%%%%%%%%%%%%%%%%%%%%%%%%%%%%%%%%%%%%%%%%%%
```

```
% REACTION KINETICS
%%%%%%%%%%%%%%%%%%%%%%%%%%%%%%%%%%%%%%%%%%%%%%%
CoAMAT = CoAMATt - C16AcylCoAMAT - C16EnoylCoAMAT - C14AcylCoAMAT -
C14EnoylCoAMAT - C12AcylCoAMAT - C12EnoylCoAMAT - C10AcylCoAMAT -
C10EnoylCoAMAT - C8AcylCoAMAT - C8EnoylCoAMAT - C6AcylCoAMAT - C6EnoylCoAMAT
- C6KetoacylCoAMAT - C4AcylCoAMAT - C4EnoylCoAMAT - C4AcetoacylCoAMAT -
AcetylCoAMAT;

vcpt1C16 = CPT1([Vcpt1, Kmcpt1C16AcylCoACYT, Kmcpt1CarCYT,
Kmcpt1C16AcylCarCYT, Kmcpt1CoACYT, Kicpt1MalCoACYT, Keqcpt1, C16AcylCoACYT,
CarCYT, C16AcylCarCYT, CoACYT, MalCoACYT, ncpt1]);
vcactC16 = CACT([Vfcact, KmcactC16AcylCarCYT, KmcactCarMAT,
KicactC16AcylCarCYT, KicactCarCYT, C16AcylCarCYT, CarMAT, C16AcylCarMAT,
CarCYT]);
vcactC14 = CACT([Vfcact, KmcactC16AcylCarCYT, KmcactCarMAT,
KicactC16AcylCarCYT, KicactCarCYT, C14AcylCarCYT, CarMAT, C14AcylCarMAT,
CarCYT]);
vcactC12 = CACT([Vfcact, KmcactC16AcylCarCYT, KmcactCarMAT,
KicactC16AcylCarCYT, KicactCarCYT, C12AcylCarCYT, CarMAT, C12AcylCarMAT,
CarCYT]);
vcactC10 = CACT([Vfcact, KmcactC16AcylCarCYT, KmcactCarMAT,
KicactC16AcylCarCYT, KicactCarCYT, C10AcylCarCYT, CarMAT, C10AcylCarMAT,
CarCYT]);
vcactC8 = CACT([Vfcact, KmcactC16AcylCarCYT, KmcactCarMAT,
KicactC16AcylCarCYT, KicactCarCYT, C8AcylCarCYT, CarMAT, C8AcylCarMAT,
CarCYT]);
vcactC6 = CACT([Vfcact, KmcactC16AcylCarCYT, KmcactCarMAT,
KicactC16AcylCarCYT, KicactCarCYT, C6AcylCarCYT, CarMAT, C6AcylCarMAT,
CarCYT]);
vcactC4 = CACT([Vfcact, KmcactC16AcylCarCYT, KmcactCarMAT,
KicactC16AcylCarCYT, KicactCarCYT, C4AcylCarCYT, CarMAT, C4AcylCarMAT,
CarCYT]);
vcpt2C16 = CPT2([sfcpt2C16, Vcpt2, Kmcpt2C16AcylCarMAT, Kmcpt2CoAMAT,
Kmcpt2C16AcylCoAMAT, Kmcpt2C16AcylCoAMAT, Kmcpt2C6AcylCoAMAT,
Kmcpt2C4AcylCoAMAT, Kmcpt2CarMAT, Keqcpt2, C16AcylCarMAT, C14AcylCarMAT,
C12AcylCarMAT, C10AcylCarMAT, C8AcylCarMAT, C6AcylCarMAT, C4AcylCarMAT,
CoAMAT, C16AcylCoAMAT, C14AcylCoAMAT, C12AcylCoAMAT, C10AcylCoAMAT,
C8AcylCoAMAT, C6AcylCoAMAT, C4AcylCoAMAT, CarMAT]);
vcpt2C14 = CPT2([sfcpt2C14, Vcpt2, Kmcpt2C16AcylCarMAT, Kmcpt2CoAMAT,
Kmcpt2C16AcylCoAMAT, Kmcpt2C16AcylCoAMAT, Kmcpt2C6AcylCoAMAT,
Kmcpt2C4AcylCoAMAT, Kmcpt2CarMAT, Keqcpt2, C14AcylCarMAT, C16AcylCarMAT,
C12AcylCarMAT, C10AcylCarMAT, C8AcylCarMAT, C6AcylCarMAT, C4AcylCarMAT,
CoAMAT, C14AcylCoAMAT, C16AcylCoAMAT, C12AcylCoAMAT, C10AcylCoAMAT,
C8AcylCoAMAT, C6AcylCoAMAT, C4AcylCoAMAT, CarMAT]);
vcpt2C12 = CPT2([sfcpt2C12, Vcpt2, Kmcpt2C16AcylCarMAT, Kmcpt2CoAMAT,
Kmcpt2C16AcylCoAMAT, Kmcpt2C16AcylCoAMAT, Kmcpt2C6AcylCoAMAT,
Kmcpt2C4AcylCoAMAT, Kmcpt2CarMAT, Keqcpt2, C12AcylCarMAT, C16AcylCarMAT,
C14AcylCarMAT, C10AcylCarMAT, C8AcylCarMAT, C6AcylCarMAT, C4AcylCarMAT,
CoAMAT, C12AcylCoAMAT, C16AcylCoAMAT, C14AcylCoAMAT, C10AcylCoAMAT,
C8AcylCoAMAT, C6AcylCoAMAT, C4AcylCoAMAT, CarMAT]);
vcpt2C10 = CPT2([sfcpt2C12, Vcpt2, Kmcpt2C16AcylCarMAT, Kmcpt2CoAMAT,
Kmcpt2C16AcylCoAMAT, Kmcpt2C16AcylCoAMAT, Kmcpt2C6AcylCoAMAT,
Kmcpt2C4AcylCoAMAT, Kmcpt2CarMAT, Keqcpt2, C10AcylCarMAT, C16AcylCarMAT,
C14AcylCarMAT, C12AcylCarMAT, C8AcylCarMAT, C6AcylCarMAT, C4AcylCarMAT,
CoAMAT, C10AcylCoAMAT, C16AcylCoAMAT, C14AcylCoAMAT, C12AcylCoAMAT,
C8AcylCoAMAT, C6AcylCoAMAT, C4AcylCoAMAT, CarMAT]);
```

```
vcpt2C8 = CPT2([sfcpt2C8, Vcpt2, Kmcpt2C16AcylCarMAT, Kmcpt2CoAMAT,
Kmcpt2C16AcylCoAMAT, Kmcpt2C16AcylCoAMAT, Kmcpt2C6AcylCoAMAT,
Kmcpt2C4AcylCoAMAT, Kmcpt2CarMAT, Keqcpt2, C8AcylCarMAT, C16AcylCarMAT,
C14AcylCarMAT, C12AcylCarMAT, C10AcylCarMAT, C6AcylCarMAT, C4AcylCarMAT,
CoAMAT, C8AcylCoAMAT, C16AcylCoAMAT, C14AcylCoAMAT, C12AcylCoAMAT,
C10AcylCoAMAT, C6AcylCoAMAT, C4AcylCoAMAT, CarMAT]);
vcpt2C6 = CPT2([sfcpt2C6, Vcpt2, Kmcpt2C16AcylCarMAT, Kmcpt2CoAMAT,
Kmcpt2C6AcylCoAMAT, Kmcpt2C16AcylCoAMAT, Kmcpt2C16AcylCoAMAT,
Kmcpt2C4AcylCoAMAT, Kmcpt2CarMAT, Keqcpt2, C6AcylCarMAT, C16AcylCarMAT,
C14AcylCarMAT, C12AcylCarMAT, C10AcylCarMAT, C8AcylCarMAT, C4AcylCarMAT,
CoAMAT, C6AcylCoAMAT, C16AcylCoAMAT, C14AcylCoAMAT, C12AcylCoAMAT,
C10AcylCoAMAT, C8AcylCoAMAT, C4AcylCoAMAT, CarMAT]);
vcpt2C4 = CPT2([sfcpt2C4, Vcpt2, Kmcpt2C16AcylCarMAT, Kmcpt2CoAMAT,
Kmcpt2C4AcylCoAMAT, Kmcpt2C16AcylCoAMAT, Kmcpt2C16AcylCoAMAT,
Kmcpt2C6AcylCoAMAT, Kmcpt2CarMAT, Keqcpt2, C4AcylCarMAT, C16AcylCarMAT,
C14AcylCarMAT, C12AcylCarMAT, C10AcylCarMAT, C8AcylCarMAT, C6AcylCarMAT,
CoAMAT, C4AcylCoAMAT, C16AcylCoAMAT, C14AcylCoAMAT, C12AcylCoAMAT,
C10AcylCoAMAT, C8AcylCoAMAT, C6AcylCoAMAT, CarMAT]);
vvlcadC16 = VLCAD([sfvlcadC16, Vvlcad, KmvlcadC16AcylCoAMAT,
KmvlcadC14AcylCoAMAT, KmvlcadC12AcylCoAMAT, KmvlcadFAD, KmcadEnoylCoAMAT,
KmvlcadFADH, Keqcad, C16AcylCoAMAT, C14AcylCoAMAT, C12AcylCoAMAT, FADtMAT,
C16EnoylCoAMAT, C14EnoylCoAMAT, C12EnoylCoAMAT, FADHMAT]);
vvlcadC14 = VLCAD([sfvlcadC14, Vvlcad, KmvlcadC14AcylCoAMAT,
KmvlcadC16AcylCoAMAT, KmvlcadC12AcylCoAMAT, KmvlcadFAD, KmcadEnoylCoAMAT,
KmvlcadFADH, Keqcad, C14AcylCoAMAT, C16AcylCoAMAT, C12AcylCoAMAT, FADtMAT,
C14EnoylCoAMAT, C16EnoylCoAMAT, C12EnoylCoAMAT, FADHMAT]);
vvlcadC12 = VLCAD([sfvlcadC12, Vvlcad, KmvlcadC12AcylCoAMAT,
KmvlcadC16AcylCoAMAT, KmvlcadC14AcylCoAMAT, KmvlcadFAD, KmcadEnoylCoAMAT,
KmvlcadFADH, Keqcad, C12AcylCoAMAT, C16AcylCoAMAT, C14AcylCoAMAT, FADtMAT,
C12EnoylCoAMAT, C16EnoylCoAMAT, C14EnoylCoAMAT, FADHMAT]);
vlcadC16 = LCAD([sflcadC16, Vlcad, KmlcadC16AcylCoAMAT, KmlcadC14AcylCoAMAT,
KmlcadC12AcylCoAMAT, KmlcadC10AcylCoAMAT, KmlcadC8AcylCoAMAT, KmlcadFAD,
KmcadEnoylCoAMAT, KmlcadFADH, Keqcad, C16AcylCoAMAT, C14AcylCoAMAT,
C12AcylCoAMAT, C10AcylCoAMAT, C8AcylCoAMAT, FADtMAT, C16EnoylCoAMAT,
C14EnoylCoAMAT, C12EnoylCoAMAT, C10EnoylCoAMAT, C8EnoylCoAMAT, FADHMAT]);
vlcadC14 = LCAD([sflcadC14, Vlcad, KmlcadC14AcylCoAMAT, KmlcadC16AcylCoAMAT,
KmlcadC12AcylCoAMAT, KmlcadC10AcylCoAMAT, KmlcadC8AcylCoAMAT, KmlcadFAD,
KmcadEnoylCoAMAT, KmlcadFADH, Keqcad, C14AcylCoAMAT, C16AcylCoAMAT,
C12AcylCoAMAT, C10AcylCoAMAT, C8AcylCoAMAT, FADtMAT, C14EnoylCoAMAT,
C16EnoylCoAMAT, C12EnoylCoAMAT, C10EnoylCoAMAT, C8EnoylCoAMAT, FADHMAT]);
vlcadC12 = LCAD([sflcadC12, Vlcad, KmlcadC12AcylCoAMAT, KmlcadC16AcylCoAMAT,
KmlcadC14AcylCoAMAT, KmlcadC10AcylCoAMAT, KmlcadC8AcylCoAMAT, KmlcadFAD,
KmcadEnoylCoAMAT, KmlcadFADH, Keqcad, C12AcylCoAMAT, C16AcylCoAMAT,
C14AcylCoAMAT, C10AcylCoAMAT, C8AcylCoAMAT, FADtMAT, C14EnoylCoAMAT,
C16EnoylCoAMAT, C14EnoylCoAMAT, C10EnoylCoAMAT, C8EnoylCoAMAT, FADHMAT]);
vlcadC10 = LCAD([sflcadC10, Vlcad, KmlcadC10AcylCoAMAT, KmlcadC16AcylCoAMAT,
KmlcadC14AcylCoAMAT, KmlcadC12AcylCoAMAT, KmlcadC8AcylCoAMAT, KmlcadFAD,
KmcadEnoylCoAMAT, KmlcadFADH, Keqcad, C10AcylCoAMAT, C16AcylCoAMAT,
C14AcylCoAMAT, C12AcylCoAMAT, C8AcylCoAMAT, FADtMAT, C10EnoylCoAMAT,
C16EnoylCoAMAT, C14EnoylCoAMAT, C12EnoylCoAMAT, C8EnoylCoAMAT, FADHMAT]);
vlcadC8  = 0;
vmcadC12 = MCAD([sfmcadC12, Vmcad, KmmcadC12AcylCoAMAT, KmmcadC10AcylCoAMAT,
KmmcadC8AcylCoAMAT, KmmcadC6AcylCoAMAT, KmmcadC4AcylCoAMAT, KmmcadFAD,
KmcadEnoylCoAMAT, KmmcadFADH, Keqcad, C12AcylCoAMAT, C10AcylCoAMAT,
C8AcylCoAMAT, C6AcylCoAMAT, C4AcylCoAMAT, FADtMAT, C12EnoylCoAMAT,
C10EnoylCoAMAT, C8EnoylCoAMAT, C6EnoylCoAMAT, C4EnoylCoAMAT, FADHMAT]);
```

```
vmcadC10 = MCAD([sfmcadC10, Vmcad, KmmcadC10AcylCoAMAT, KmmcadC12AcylCoAMAT,
KmmcadC8AcylCoAMAT, KmmcadC6AcylCoAMAT, KmmcadC4AcylCoAMAT, KmmcadFAD,
KmcadEnoylCoAMAT, KmmcadFADH, Keqcad, C10AcylCoAMAT, C12AcylCoAMAT,
C8AcylCoAMAT, C6AcylCoAMAT, C4AcylCoAMAT, FADtMAT, C10EnoylCoAMAT,
C12EnoylCoAMAT, C8EnoylCoAMAT, C6EnoylCoAMAT, C4EnoylCoAMAT, FADHMAT]);
vmcadC8 = MCAD([sfmcadC8, Vmcad, KmmcadC8AcylCoAMAT, KmmcadC12AcylCoAMAT,
KmmcadC10AcylCoAMAT, KmmcadC6AcylCoAMAT, KmmcadC4AcylCoAMAT, KmmcadFAD,
KmcadEnoylCoAMAT, KmmcadFADH, Keqcad, C8AcylCoAMAT, C12AcylCoAMAT,
C10AcylCoAMAT, C6AcylCoAMAT, C4AcylCoAMAT, FADtMAT, C8EnoylCoAMAT,
C12EnoylCoAMAT, C10EnoylCoAMAT, C6EnoylCoAMAT, C4EnoylCoAMAT, FADHMAT]);
vmcadC6 = MCAD([sfmcadC6, Vmcad, KmmcadC6AcylCoAMAT, KmmcadC12AcylCoAMAT,
KmmcadC10AcylCoAMAT, KmmcadC8AcylCoAMAT, KmmcadC4AcylCoAMAT, KmmcadFAD,
KmcadEnoylCoAMAT, KmmcadFADH, Keqcad, C6AcylCoAMAT, C12AcylCoAMAT,
C10AcylCoAMAT, C8AcylCoAMAT, C4AcylCoAMAT, FADtMAT, C6EnoylCoAMAT,
C12EnoylCoAMAT, C10EnoylCoAMAT, C8EnoylCoAMAT, C4EnoylCoAMAT, FADHMAT]);
vscadC4 = SCAD([sfscadC4, Vscad, KmscadC4AcylCoAMAT, KmscadC6AcylCoAMAT,
KmscadFAD, KmcadEnoylCoAMAT, KmscadFADH, Keqcad, C4AcylCoAMAT, C6AcylCoAMAT,
FADtMAT, C4EnoylCoAMAT, C6EnoylCoAMAT, FADHMAT]);
vcrmsC6 = CRMS([sfcrotC6, Vcrot, KmcrotC6EnoylCoAMAT, KmcrotC4EnoylCoAMAT,
KicrotC4AcetoacylCoA, Keqcrot, C6EnoylCoAMAT, C4EnoylCoAMAT, sfmschadC6,
Vmschad, KmmschadC6HydroxyacylCoAMAT, KmmschadNADMAT,
KmmschadC6KetoacylCoAMAT, KmmschadC4AcetoacylCoAMAT, KmmschadNADHMAT,
Keqmschad, NAD, C6KetoacylCoAMAT, C4AcetoacylCoAMAT, NADHMAT,
C4AcetoacylCoAMAT]);
vcrmsC4 = CRMS([sfcrotC4, Vcrot, KmcrotC4EnoylCoAMAT, KmcrotC6EnoylCoAMAT,
KicrotC4AcetoacylCoA, Keqcrot, C4EnoylCoAMAT, C6EnoylCoAMAT, sfmschadC4,
Vmschad, KmmschadC4HydroxyacylCoAMAT, KmmschadNADMAT,
KmmschadC4AcetoacylCoAMAT, KmmschadC6KetoacylCoAMAT, KmmschadNADHMAT,
Keqmschad, NAD, C4AcetoacylCoAMAT, C6KetoacylCoAMAT, NADHMAT,
C4AcetoacylCoAMAT]);
vmckatC6  = MCKATA([sfmckatC6, Vmckat, KmmckatC6KetoacylCoAMAT,
KmmckatC4AcetoacylCoAMAT, KmmckatCoAMAT, KmmckatC4AcylCoAMAT,
KmmckatAcetylCoAMAT, Keqmckat, C6KetoacylCoAMAT, C4AcetoacylCoAMAT, CoAMAT,
C4AcylCoAMAT, C16AcylCoAMAT, C14AcylCoAMAT, C12AcylCoAMAT, C10AcylCoAMAT,
C8AcylCoAMAT, C6AcylCoAMAT, AcetylCoAMAT]);
vmckatC4  = MCKATB([sfmckatC4, Vmckat, KmmckatC4AcetoacylCoAMAT,
KmmckatC6KetoacylCoAMAT, KmmckatCoAMAT, KmmckatC4AcylCoAMAT,
KmmckatAcetylCoAMAT, Keqmckat, C4AcetoacylCoAMAT, C6KetoacylCoAMAT, CoAMAT,
C4AcylCoAMAT, C16AcylCoAMAT, C14AcylCoAMAT, C12AcylCoAMAT, C10AcylCoAMAT,
C8AcylCoAMAT, C6AcylCoAMAT, AcetylCoAMAT]);
vmtpC16 = MTP([sfmtpC16, Vmtp, KmmtpC16EnoylCoAMAT, KmmtpNADMAT, KmmtpCoAMAT,
KmmtpC16AcylCoAMAT, KmmtpNADHMAT, KmmtpAcetylCoAMAT, Keqmtp, C16EnoylCoAMAT,
C14EnoylCoAMAT, C12EnoylCoAMAT, C10EnoylCoAMAT, C8EnoylCoAMAT, NADtMAT,
CoAMAT, C14AcylCoAMAT, C16AcylCoAMAT, C12AcylCoAMAT, C10AcylCoAMAT,
C8AcylCoAMAT, C6AcylCoAMAT, NADHMAT, AcetylCoAMAT]);
vmtpC14 = MTP([sfmtpC14, Vmtp, KmmtpC16EnoylCoAMAT, KmmtpNADMAT, KmmtpCoAMAT,
KmmtpC16AcylCoAMAT, KmmtpNADHMAT, KmmtpAcetylCoAMAT, Keqmtp, C14EnoylCoAMAT,
C16EnoylCoAMAT, C12EnoylCoAMAT, C10EnoylCoAMAT, C8EnoylCoAMAT, NADtMAT,
CoAMAT, C12AcylCoAMAT, C16AcylCoAMAT, C14AcylCoAMAT, C10AcylCoAMAT,
C8AcylCoAMAT, C6AcylCoAMAT, NADHMAT, AcetylCoAMAT]);
vmtpC12 = MTP([sfmtpC12, Vmtp, KmmtpC16EnoylCoAMAT, KmmtpNADMAT, KmmtpCoAMAT,
KmmtpC16AcylCoAMAT, KmmtpNADHMAT, KmmtpAcetylCoAMAT, Keqmtp, C12EnoylCoAMAT,
C16EnoylCoAMAT, C14EnoylCoAMAT, C10EnoylCoAMAT, C8EnoylCoAMAT, NADtMAT,
CoAMAT, C10AcylCoAMAT, C16AcylCoAMAT, C14AcylCoAMAT, C12AcylCoAMAT,
C8AcylCoAMAT, C6AcylCoAMAT, NADHMAT, AcetylCoAMAT]);
vmtpC10 = MTP([sfmtpC10, Vmtp, KmmtpC16EnoylCoAMAT, KmmtpNADMAT, KmmtpCoAMAT,
KmmtpC16AcylCoAMAT, KmmtpNADHMAT, KmmtpAcetylCoAMAT, Keqmtp, C10EnoylCoAMAT,
```

```
C16EnoylCoAMAT, C14EnoylCoAMAT, C12EnoylCoAMAT, C8EnoylCoAMAT, NADtMAT,
CoAMAT, C8AcylCoAMAT, C16AcylCoAMAT, C14AcylCoAMAT, C12AcylCoAMAT,
C10AcylCoAMAT, C6AcylCoAMAT, NADHMAT, AcetylCoAMAT]);
vmtpC8  = MTP([sfmtpC8, Vmtp, KmmtpC16EnoylCoAMAT, KmmtpNADMAT, KmmtpCoAMAT,
KmmtpC16AcylCoAMAT, KmmtpNADHMAT, KmmtpAcetylCoAMAT, Keqmtp, C8EnoylCoAMAT,
C16EnoylCoAMAT, C14EnoylCoAMAT, C12EnoylCoAMAT, C10EnoylCoAMAT, NADtMAT,
CoAMAT, C6AcylCoAMAT, C16AcylCoAMAT, C14AcylCoAMAT, C12AcylCoAMAT,
C10AcylCoAMAT, C8AcylCoAMAT, NADHMAT, AcetylCoAMAT]);
vacesink = RES([Ksacesink, AcetylCoAMAT, K1acesink]);

%%%%%%%%%%%%%%%%%%%%%%%%%%%%%%%%%%%%%%%%%%%%%%%
% DIFFERENTIAL EQUATIONS
%%%%%%%%%%%%%%%%%%%%%%%%%%%%%%%%%%%%%%%%%%%%%%%
output(1) = (vcpt1C16 - vcactC16)/VCYT;
output(2) = (vcactC16 - vcpt2C16)/VMAT;
output(3) = (vcpt2C16 - vvlcadC16 - vlcadC16)/VMAT;
output(4) = (vvlcadC16 + vlcadC16 - vmtpC16)/VMAT;
output(5) = - vcactC14/VCYT;
output(6) = (vcactC14 - vcpt2C14)/VMAT;
output(7) = (vcpt2C14 + vmtpC16 - vvlcadC14 - vlcadC14)/VMAT;
output(8) = (vvlcadC14 + vlcadC14 - vmtpC14)/VMAT;
output(9) = - vcactC12/VCYT;
output(10) = (vcactC12 - vcpt2C12)/VMAT;
output(11) = (vcpt2C12 + vmtpC14 - vvlcadC12 - vlcadC12 - vmcadC12)/VMAT;
output(12) = (vvlcadC12 + vlcadC12 + vmcadC12 - vmtpC12)/VMAT;
output(13) = - vcactC10/VCYT;
output(14) = (vcactC10 - vcpt2C10)/VMAT;
output(15) = (vcpt2C10 + vmtpC12 - vlcadC10 - vmcadC10)/VMAT;
output(16) = (vlcadC10 + vmcadC10 - vmtpC10)/VMAT;
output(17) = - vcactC8/VCYT;
output(18) = (vcactC8 - vcpt2C8)/VMAT;
output(19) = (vcpt2C8 + vmtpC10 - vlcadC8 - vmcadC8)/VMAT;
output(20) = (vlcadC8 + vmcadC8 - vmtpC8)/VMAT;
output(21) = - vcactC6/VCYT;
output(22) = (vcactC6 - vcpt2C6)/VMAT;
output(23) = (vcpt2C6 + vmtpC8 - vmcadC6)/VMAT;
output(24) = (vmcadC6 - vcrmsC6)/VMAT;
output(25) = (vcrmsC6 - vmckatC6)/VMAT;
output(26) = - vcactC4/VCYT;
output(27) = (vcactC4 - vcpt2C4)/VMAT;
output(28) = (vcpt2C4 + vmckatC6 - vscadC4)/VMAT;
output(29) = (vscadC4 - vcrmsC4)/VMAT;
output(30) = (vcrmsC4 - vmckatC4)/VMAT;
output(31) = (1/VMAT)*(vmtpC16 + vmtpC14 + vmtpC12 + vmtpC10 + vmtpC8 +
vmckatC6 + 2*vmckatC4 - vacesink);

%%%%%%%%%%%%%%%%%%%%%%%%%%%%%%%%%%%%%%%%%%%%%%%
% RETURN VALUES
%%%%%%%%%%%%%%%%%%%%%%%%%%%%%%%%%%%%%%%%%%%%%%%
% return a column vector
output = output(:);
%end;

function output = CPT1(input)

V = input(1);
```

```
Kms1 = input(2);
Kms2 = input(3);
Kmp1 = input(4);
Kmp2 = input(5);
Ki1 = input(6);
Keq = input(7);
S1 = input(8);
S2 = input(9);
P1 = input(10);
P2 = input(11);
I1 = input(12);
n = input(13);

output = (V*((S1*S2)/(Kms1*Kms2) - (P1*P2)/(Kms1*Kms2*Keq)))/((1 + S1/Kms1 +
P1/Kmp1 + (I1/Ki1)^n)*(1 + S2/Kms2 + P2/Kmp2));
%end;

function output = CACT(input)

Vf = input(1);
Kms1 = input(2);
Kms2 = input(3);
Kis1 = input(4);
Kip2 = input(5);
S1 = input(6);
S2 = input(7);
P1 = input(8);
P2 = input(9);

output = Vf*(S1*S2 - P1*P2)/(S1*S2 + Kms2*S1 + Kms1*S2*(1 + P2/Kip2) +
Kms2*P1*(1 + S1/Kis1) + P2*(Kms1 + P1));
%end;

function output = CPT2(input)

sf = input(1);
V = input(2);
Kms1 = input(3);
Kms8 = input(4);
Kmp1 = input(5);
Kmp2 = input(6);
Kmp6 = input(7);
Kmp7 = input(8);
Kmp8 = input(9);
Keq = input(10);
S1 = input(11);
S2 = input(12);
S3 = input(13);
S4 = input(14);
S5 = input(15);
S6 = input(16);
S7 = input(17);
S8 = input(18);
P1 = input(19);
P2 = input(20);
P3 = input(21);
```

```
P4 = input(22);
P5 = input(23);
P6 = input(24);
P7 = input(25);
P8 = input(26);
output = (sf*V*((S1*S8)/(Kms1*Kms8) - (P1*P8)/(Kms1*Kms8*Keq)))/((1 + S1/Kms1
+ P1/Kmp1 + S2/Kms1 + P2/Kmp2 + S3/Kms1 + P3/Kmp2 + S4/Kms1 + P4/Kmp2 +
S5/Kms1 + P5/Kmp2 + S6/Kms1 + P6/Kmp6 + S7/Kms1 + P7/Kmp7)*(1 + S8/Kms8 +
P8/Kmp8));
%end;

function output = VLCAD(input)

sf = input(1);
V = input(2);
Kms1 = input(3);
Kms2 = input(4);
Kms3 = input(5);
Kms4 = input(6);
Kmp1 = input(7);
Kmp4 = input(8);
Keq = input(9);
S1 = input(10);
S2 = input(11);
S3 = input(12);
S4 = input(13);
P1 = input(14);
P2 = input(15);
P3 = input(16);
P4 = input(17);

output = (sf*V*((S1*(S4 - P4))/(Kms1*Kms4) - (P1*P4)/(Kms1*Kms4*Keq)))/((1 +
S1/Kms1 + P1/Kmp1 + S2/Kms2 + P2/Kmp1 + S3/Kms3 + P3/Kmp1)*(1 + (S4 -
P4)/Kms4 + P4/Kmp4));
%end;

function output = LCAD(input)

sf = input(1);
V = input(2);
Kms1 = input(3);
Kms2 = input(4);
Kms3 = input(5);
Kms4 = input(6);
Kms5 = input(7);
Kms6 = input(8);
Kmp1 = input(9);
Kmp6 = input(10);
Keq = input(11);
S1 = input(12);
S2 = input(13);
S3 = input(14);
S4 = input(15);
S5 = input(16);
S6 = input(17);
P1 = input(18);
```

```
P2 = input(19);
P3 = input(20);
P4 = input(21);
P5 = input(22);
P6 = input(23);

output = (sf*V*((S1*(S6 - P6))/(Kms1*Kms6) - (P1*P6)/(Kms1*Kms6*Keq)))/((1 +
S1/Kms1 + P1/Kmp1 + S2/Kms2 + P2/Kmp1 + S3/Kms3 + P3/Kmp1 + S4/Kms4 + P4/Kmp1
+ S5/Kms5 + P5/Kmp1)*(1 + (S6 - P6)/Kms6 + P6/Kmp6));
%end;

function output = MCAD(input)

sf = input(1);
V = input(2);
Kms1 = input(3);
Kms2 = input(4);
Kms3 = input(5);
Kms4 = input(6);
Kms5 = input(7);
Kms6 = input(8);
Kmp1 = input(9);
Kmp6 = input(10);
Keq = input(11);
S1 = input(12);
S2 = input(13);
S3 = input(14);
S4 = input(15);
S5 = input(16);
S6 = input(17);
P1 = input(18);
P2 = input(19);
P3 = input(20);
P4 = input(21);
P5 = input(22);
P6 = input(23);

output = (sf*V*((S1*(S6 - P6))/(Kms1*Kms6) - (P1*P6)/(Kms1*Kms6*Keq)))/((1 +
S1/Kms1 + P1/Kmp1 + S2/Kms2 + P2/Kmp1 + S3/Kms3 + P3/Kmp1 + S4/Kms4 + P4/Kmp1
+ S5/Kms5 + P5/Kmp1)*(1 + (S6 - P6)/Kms6 + P6/Kmp6));
%end;

function output = SCAD(input)

sf = input(1);
V = input(2);
Kms1 = input(3);
Kms2 = input(4);
Kms3 = input(5);
Kmp1 = input(6);
Kmp3 = input(7);
Keq = input(8);
S1 = input(9);
S2 = input(10);
S3 = input(11);
P1 = input(12);
```

```
P2 = input(13);
P3 = input(14);

output = (sf*V*((S1*(S3 - P3))/(Kms1*Kms3) - (P1*P3)/(Kms1*Kms3*Keq)))/((1 +
S1/Kms1 + P1/Kmp1 + S2/Kms2 + P2/Kmp1)*(1 + (S3 - P3)/Kms3 + P3/Kmp3));

%end;

function output = CRMS(input)

sfc = input(1);
Vc = input(2);
Kms1 = input(3);
Kms2 = input(4);
Ki = input(5);
Keqc = input(6);
S1 = input(7);
S2 = input(8);
sfm = input(9);
Vm = input(10);
Kmms1 = input(11);
Kmp0 = input(12);
Kmp1 = input(13);
Kmp2 = input(14);
Kmp3 = input(15);
Keqm = input(16);
P0 = input(17);
P1 = input(18);
P2 = input(19);
P3 = input(20);
I1 = input(21);

output = (sfc*Vc*sfm*Vm*((S1*P0)/(Kms1*Kmms1*Kmp0) -
(P1*P3)/(Kms1*Kmms1*Kmp0*Keqc*Keqm)))/(sfc*Vc*(1 + P1/Kmp1 + P2/Kmp2)*(1 +
P0/Kmp0 + P3/Kmp3)/(Kms1*Keqc)+sfm*Vm*P0*(1 + S1/Kms1 + S2/Kms2 +
I1/Ki)/(Kmms1*Kmp0));
%end;

function output = MCKATA(input)

sf = input(1);
V = input(2);
Kms1 = input(3);
Kms2 = input(4);
Kms8 = input(5);
Kmp1 = input(6);
Kmp8 = input(7);
Keq = input(8);
S1 = input(9);
S2 = input(10);
S8 = input(11);
P1 = input(12);
P2 = input(13);
P3 = input(14);
P4 = input(15);
```

```
P5 = input(16);
P6 = input(17);
P7 = input(18);
P8 = input(19);

output = (sf*V*((S1*S8)/(Kms1*Kms8) - (P1*P8)/(Kms1*Kms8*Keq)))/((1 + S1/Kms1
+ P1/Kmp1 + S2/Kms2 + P2/Kmp1 + P3/Kmp1 + P4/Kmp1 + P5/Kmp1 + P6/Kmp1 +
P7/Kmp1 + P8/Kmp8)*(1 + S8/Kms8 + P8/Kmp8));
%end;

function output = MCKATB(input)

sf = input(1);
V = input(2);
Kms1 = input(3);
Kms2 = input(4);
Kms8 = input(5);
Kmp1 = input(6);
Kmp8 = input(7);
Keq = input(8);
S1 = input(9);
S2 = input(10);
S8 = input(11);
P1 = input(12);
P2 = input(13);
P3 = input(14);
P4 = input(15);
P5 = input(16);
P6 = input(17);
P7 = input(18);
P8 = input(19);

output = (sf*V*((S1*S8)/(Kms1*Kms8) - (P8*P8)/(Kms1*Kms8*Keq)))/((1 + S1/Kms1
+ P1/Kmp1 + S2/Kms2 + P2/Kmp1 + P3/Kmp1 + P4/Kmp1 + P5/Kmp1 + P6/Kmp1 +
P7/Kmp1 + P8/Kmp8)*(1 + S8/Kms8 + P8/Kmp8));
%end;

function output = MTP(input)
sf = input(1);
V = input(2);
Kms1 = input(3);
Kms7 = input(4);
Kms8 = input(5);
Kmp1 = input(6);
Kmp7 = input(7);
Kmp8 = input(8);
Keq = input(9);
S1 = input(10);
S2 = input(11);
S3 = input(12);
S4 = input(13);
S5 = input(14);
S7 = input(15);
S8 = input(16);
P1 = input(17);
P2 = input(18);
```

```
P3 = input(19);
P4 = input(20);
P5 = input(21);
P6 = input(22);
P7 = input(23);
P8 = input(24);

output = (sf*V*((S1*(S7 - P7)*S8)/(Kms1*Kms7*Kms8) -
(P1*P7*P8)/(Kms1*Kms7*Kms8*Keq)))/((1 + S1/Kms1 + P1/Kmp1 + S2/Kms1 + P2/Kmp1
+ S3/Kms1 + P3/Kmp1 + S4/Kms1 + P4/Kmp1 + S5/Kms1 + P5/Kmp1 + P6/Kmp1)*(1 +
(S7 - P7)/Kms7 + P7/Kmp7)*(1 + S8/Kms8 + P8/Kmp8));
%end;

function output = RES(input)

Ks = input(1);
S = input(2);
K1 = input(3);

output = Ks*(S - K1);
%end;
```
